# The flavonoid procyanidin C1 has senotherapeutic activity and increases lifespan in mice

**DOI:** 10.1038/s42255-021-00491-8

**Published:** 2021-12-06

**Authors:** Qixia Xu, Qiang Fu, Zi Li, Hanxin Liu, Ying Wang, Xu Lin, Ruikun He, Xuguang Zhang, Zhenyu Ju, Judith Campisi, James L. Kirkland, Yu Sun

**Affiliations:** 1 CAS Key Laboratory of Tissue Microenvironment and Tumour, Shanghai Institute of Nutrition and Health, University of Chinese Academy of Sciences, Chinese Academy of Sciences, Shanghai, China; 2 Institute of Health Sciences, Shanghai Jiao Tong University School of Medicine & Shanghai Institutes for Biological Sciences, Chinese Academy of Sciences, Shanghai, China; 3 Department of Pharmacology, Institute of Aging Medicine, Binzhou Medical University, Yantai, China; 4 Shanghai Institute of Nutrition and Health, Chinese Academy of Sciences, Shanghai, China; 5 Science & Technology Centre, By-Health Corp. Ltd., Guangzhou, China; 6 Key Laboratory of Regenerative Medicine of Ministry of Education, Guangzhou Regenerative Medicine and Health Guangdong Laboratory, Institute of Aging and Regenerative Medicine, Jinan University, Guangzhou, China; 7 Buck Institute for Research on Aging, Novato, CA USA; 8 Life Sciences Division, Lawrence Berkeley National Laboratory, Berkeley, CA USA; 9 Robert and Arlene Kogod Center on Aging, Mayo Clinic, Rochester, MN USA; 10 Department of Medicine and VAPSHCS, University of Washington, Seattle, WA USA

**Keywords:** Metabolism, Senescence, Drug development, Ageing

## Abstract

Ageing-associated functional decline of organs and increased risk for age-related chronic pathologies is driven in part by the accumulation of senescent cells, which develop the senescence-associated secretory phenotype (SASP). Here we show that procyanidin C1 (PCC1), a polyphenolic component of grape seed extract (GSE), increases the healthspan and lifespan of mice through its action on senescent cells. By screening a library of natural products, we find that GSE, and PCC1 as one of its active components, have specific effects on senescent cells. At low concentrations, PCC1 appears to inhibit SASP formation, whereas it selectively kills senescent cells at higher concentrations, possibly by promoting production of reactive oxygen species and mitochondrial dysfunction. In rodent models, PCC1 depletes senescent cells in a treatment-damaged tumour microenvironment and enhances therapeutic efficacy when co-administered with chemotherapy. Intermittent administration of PCC1 to either irradiated, senescent cell-implanted or naturally aged old mice alleviates physical dysfunction and prolongs survival. We identify PCC1 as a natural senotherapeutic agent with in vivo activity and high potential for further development as a clinical intervention to delay, alleviate or prevent age-related pathologies.

## Abstract (web summary)

The polyphenol procyanidin C1, a compound found in grape seeds, possesses senomorphic or senolytic activity and is shown to extend the healthspan and survival of old mice and in various models of senescence-associated disability.

## Main

Ageing is one of the biggest risk factor for chronic disorders, including cardiovascular diseases, metabolic disorders, neurodegenerative pathologies and diverse malignancies, which together account for the bulk of morbidity, mortality and health costs globally^1^. Considerable progress has been made over recent years to develop specific agents to treat individual age-related conditions, such as type 2 diabetes, osteoporosis, skeletal fragility and vascular dysfunction. However, the combined effect of these drugs in controlling morbidity and mortality of chronic diseases has been modest, and these diseases tend to occur in synchrony as multimorbidities, with prevalence increasing exponentially after 70 years of age^2^.

Several major factors affecting healthspan and lifespan have been identified through studies across a range of species and defined as ageing mechanisms that can be categorized into nine hallmarks^3^. Of these fundamental ageing mechanisms, cellular senescence has received substantial attention, as it represents a druggable process that prevents or delays multiple ageing comorbidities^4^. First reported in the 1960s, cellular senescence refers to a cellular state involving essentially irreversible replicative arrest, profound chromatin changes, apoptosis resistance and increased protein synthesis, frequently culminating in overproduction of pro-inflammatory cytokines, a feature termed the SASP, which is thought to drive ageing phenotypes and various age-related pathologies^5^. Ablation of senescent cells positive for the senescence marker p16^INK4A^ mitigates tissue degeneration and extends animal healthspan, supporting the contention that senescent cells play a causative role in organismal ageing^6,7^.

Success in preclinical studies has inspired the initiation of proof-of-concept clinical trials involving senolytics for several human diseases with the potential to decrease the burden of in vivo senescent cells through selective pharmacological elimination^8–10^. Since the first discovery in 2015 (ref. ^11^), a handful of synthetic or small-molecule senolytic agents are now known. Targeting strategies are mainly based on the resistance mechanism of senescent cells to apoptosis, which appears to depend on senescence-associated anti-apoptotic pathways that allow senescent cell survival for extended periods^12,13^. Intermittent administration of senolytics holds the potential to reduce the risk of patients developing adverse conditions, minimize off-target effects of drugs and prevent development of drug resistance of senescent cells, which do not divide, a characteristic that sets them apart from cancer cells, as cancer cells frequently acquire advantageous mutations providing resistance against anticancer therapies. However, most reported senolytics are dependent on cell lineage or cell type or, alternatively, exhibit substantial cytotoxicity in vivo, thus limiting their potential use for clinical purposes.

In this study, we screened a natural product medicinal library composed of anti-ageing agents and identified several candidates including GSE. Further analysis revealed that PCC1, a B type trimer epicatechin component of GSE flavonoids, plays a major role in inhibiting SASP expression at low concentrations and killing senescent cells at higher concentrations, the latter through inducing apoptosis. Preclinical data suggested that, in combination with classic chemotherapy, PCC1 can significantly reduce tumour size and prolong survival in several mouse models. Thus, PCC1 represents a new class of phytochemical senolytics isolated from natural sources that delay ageing and ameliorate age-related disorders and warrants further exploration as a potential geroprotective agent in clinical medicine.

## Results

### Low concentrations of GSE restrain SASP expression

**Extended Data Fig. 1:**
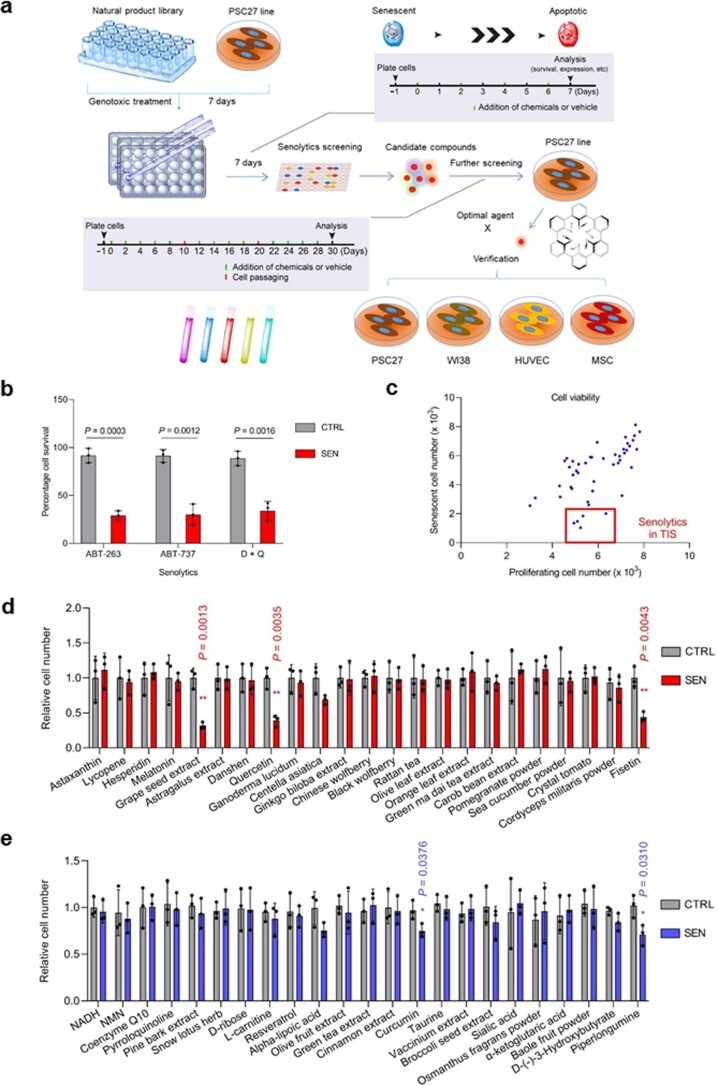
*In vitro* screening of a natural product library with human stromal cells. (**a**) Schematic workflow of cell-based screening strategy for a natural library of medicinal agents. (**b**) Quantification of cell survival of control (CTRL) and senescent (SEN) PSC27 after treatment with ABT-263 (1.25 μM), ABT-737 (10 μM) or Dasatinib (250 nM) combined with Quercetin (50 μM) for 3 d. (**c**) Screening results after pharmacological assays. Library agents were assessed at a concentration of 3 μg/ml with 5.0 × 10^3^ cells for 3 d. Hits were selected based on their ability to specifically kill senescent cells. Each blue dot represents a single agent as the mean of three replicates. Red rectangular region denotes potential candidates of senolytics in the case of TIS. (**d**) Appraisal of the effects of randomly selected agents (23 representative candidates out of 46 in the library, each applied at 1 μg/ml) on the survival of CTRL and SEN cells. (**e**) Assessment of the effects of remaining agents (23 candidates out of 46 in the library, each applied at 1 μg/ml) on the survival of CTRL and SEN cells. For all datasets, samples were collected for analyses 3 days after treatment with individual agents in culture condition. TIS, therapy-induced senescence. Data in **b**, **d** and **e** are shown as mean ± SD and representative of 3 independent biological replicates, with *P* values calculated by a two-sided *t*-test. *, *P* < 0.05; **, *P* < 0.01. Source data

In an effort to identify new compounds that can effectively modulate senescent cells, unbiased agent screening was performed with a phytochemical library composed of 46 plant-derived medicinal agents (PDMA library). We employed a primary normal human prostate stromal cell line, PSC27, as a cell-based model for this purpose. Composed mainly of fibroblasts but with a minor percentage of non-fibroblast cell lineages including endothelial cells and smooth muscle cells, PSC27 is a primary cell line per se and develops a typical SASP after exposure to stressors such as genotoxic chemotherapy or ionizing radiation^14–17^. We treated these cells with a pre-optimized sublethal dose of bleomycin (50 μg ml^−1^) and observed increased senescence-associated β-galactosidase (SA-β-Gal) staining, decreased 5-bromodeoxyuridine incorporation and elevated DNA-damage repair (DDR) foci 7–10 d after (Supplementary Fig. 1a–c). We set up a screening strategy to compare the effects that individual medicinal products had on the survival and expression profile of senescent cells (Extended Data Fig. 1a).

One promising advantage of senolytic agents is to selectively induce programmed death of senescent cells, such as ABT-263, ABT-737 and the combined use of dasatinib and quercetin^11,18,19^. We first tested the efficacy of these geroprotective drugs against senescent PSC27 cells to demonstrate its potential as an experimental cell model for drug screening. Our preliminary data suggested that each of these compounds significantly depleted senescent cells but not proliferating cells, thus confirming the feasibility of using this stromal line for further studies (Extended Data Fig. 1b). Upon large-scale screening of the PDMA library, we identified several compounds with the potential to selectively kill senescent cells in culture (Extended Data Fig. 1c–e).

**Extended Data Fig. 2:**
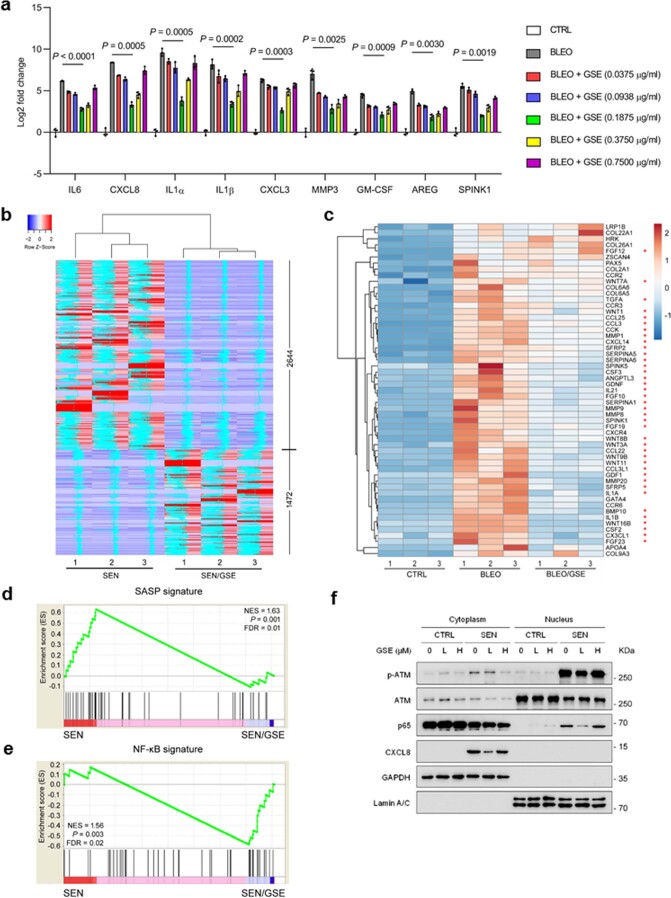
Bioinformatics profiling of the effect of GSE on genome-wide expression of senescent cells. (**a**) Quantitative analysis of SASP factor expression at transcription level upon treatment of BLEO-induced senescent cells with different concentrations of GSE in culture. *P* values were calculated by one-way ANOVA with Tukey’s multiple-comparison test. (**b**) Heatmap depicting the expression landscape of senescent cells and their counterparts exposed to GSE (0.1875 μg/ml). Note there were 2644 and 1472 genes, expression of which was significantly downregulated and upregulated by GSE, respectively. (**c**) Heatmap depicting the expression profiles of human genes in CTRL cells, SEN cells or SEN cells treated by GSE. According to the fold change of transcript expression, the top 50 genes are displayed. (**d**) GSEA plot of significant gene set indicative of a typical SASP. (**e**) GSEA plot of significant gene set associated with the activation of NF-κB signaling. For **d** and **e**, *P* values were calculated by one-way ANOVA with Tukey’s *post hoc* comparison. (**f**) Immunoblot analysis of DNA damage repair signaling, p65 nuclear translocation and CXCL8 expression in PSC27 cells (CTRL or SEN) exposed to GSE treatment. L, a representative low concentration (0.1875 μg/ml). H, a representative high concentration (3.7500 μg/ml). Cellular senescence was induced by the genotoxic chemical bleomycin (BLEO), which causes a typical TIS. For all datasets, cells were collected for analyses 3 days after GSE treatment in culture condition. Data in a and **f** are representative of 3 independent biological replicates. Source data

Among the agents showing preliminary anti-senescence effects were GSE, quercetin, fisetin, curcumin and piperlongumine (Extended Data Fig. 1d,e). Quercetin and fisetin share similar chemical structures, exert similar medicinal effects and are both known senolytics^11,20,21^. Curcumin and piperlongumine are also natural compounds with recently discovered senolytic potential^22,23^. We chose to focus on GSE, which remained a largely underexplored source. Under in vitro conditions, GSE suppressed the SASP with maximal efficiency at 0.1875 μg ml^−1^ (Extended Data Fig. 2a), which fits with the property of senomorphics^24^. Lower or higher concentrations of GSE were less efficacious, perhaps due to the induction of cellular stress responses as a result of increased cytotoxicity (Extended Data Fig. 2a). Using RNA-seq, we found that treatment with GSE significantly altered the expression profile of senescence cells, with 2,644 genes downregulated and 1,472 genes upregulated at a fold change of 2.0 per gene (*P* < 0.01) (Extended Data Fig. 2b). Although expression of a few genes unrelated to the SASP showed a similar tendency as that of typical SASP factors (Extended Data Fig. 2c), data from our gene set enrichment analysis (GSEA) supported reduced expression of molecular signatures of the SASP or activation of the nuclear factor (NF)-κB complex, which is a key mediator of the pro-inflammatory phenotype (Extended Data Fig. 2d,e).

Nuclear translocation of p65, one of the major subunits of the NF-κB complex, was observed in senescent cells, consistent with its functional engagement in SASP expression^14^ (Extended Data Fig. 2f). Of note, this tendency was substantially antagonized by GSE at low concentrations (such as 0.1875 μg ml^−1^). Conversely, activation of NF-κB signalling was not suppressed but rather appeared enhanced when GSE was used at higher concentrations (such as 3.7500 μg ml^−1^), suggesting differential responses of senescent cells in these treatment conditions. Activation of DDR signalling, as evidenced by phosphorylation of ATM kinase in nuclear fractions, and expression of the C–X–C motif chemokine ligand (CXCL)8, one of the SASP hallmark factors, as observed in cytoplasmic fractions, were consistent with NF-κB activation in these settings (Extended Data Fig. 2f).

**Extended Data Fig. 3:**
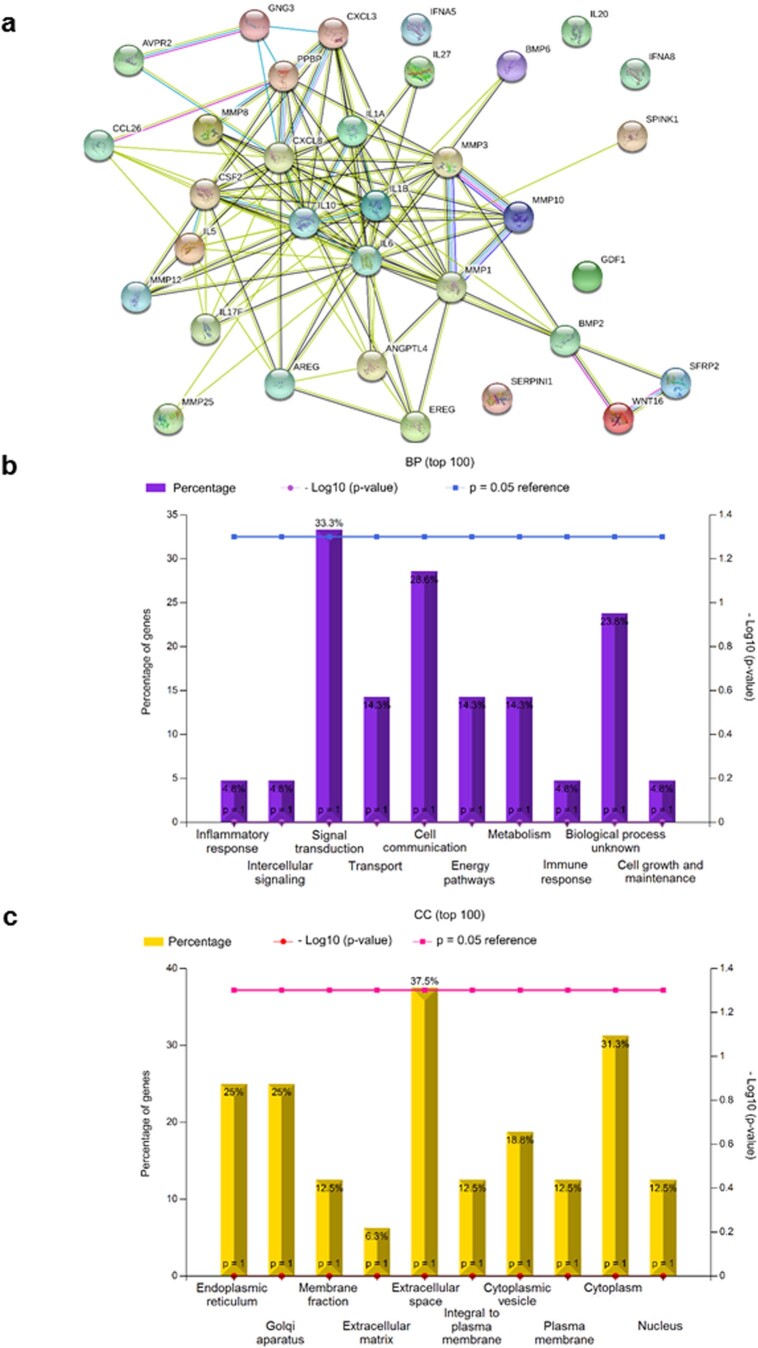
Bioinformatics profiling of the effect of GSE on the intracellular signaling network of senescent cells. (**a**) Mapping of protein-protein interaction (PPI) for BLEO-upregulated but GSE-downregulated genes by STRING program. (**b**-**c**) Column charts depicting the biological processes (BP) (**b**) and cellular components (CC) (**c**) pronouncedly associated with transcripts upregulated by BLEO but downregulated by GSE as revealed by GO analysis.

Protein–protein interaction profiling revealed a highly active network involving multiple factors significantly upregulated upon cellular senescence but downregulated once cells were exposed to GSE (Extended Data Fig. 3a). Gene ontology profiling revealed that these molecules are functionally engaged in biological processes and associated with cellular components generally consistent with the secreted nature of the SASP (Extended Data Fig. 3b,c). Thus, GSE is a natural product that holds the potential to control the pro-inflammatory profile of senescent cells, the SASP, when used within a specific concentration range. Although GSE was not the only natural product with senolytic efficacy in our cell-based assays (Extended Data Fig. 1d,e), our subsequent study largely focused on GSE, as its geroprotective capacity appeared particularly striking.

### GSE has senolytic activity at high concentrations

**Fig. 1:**
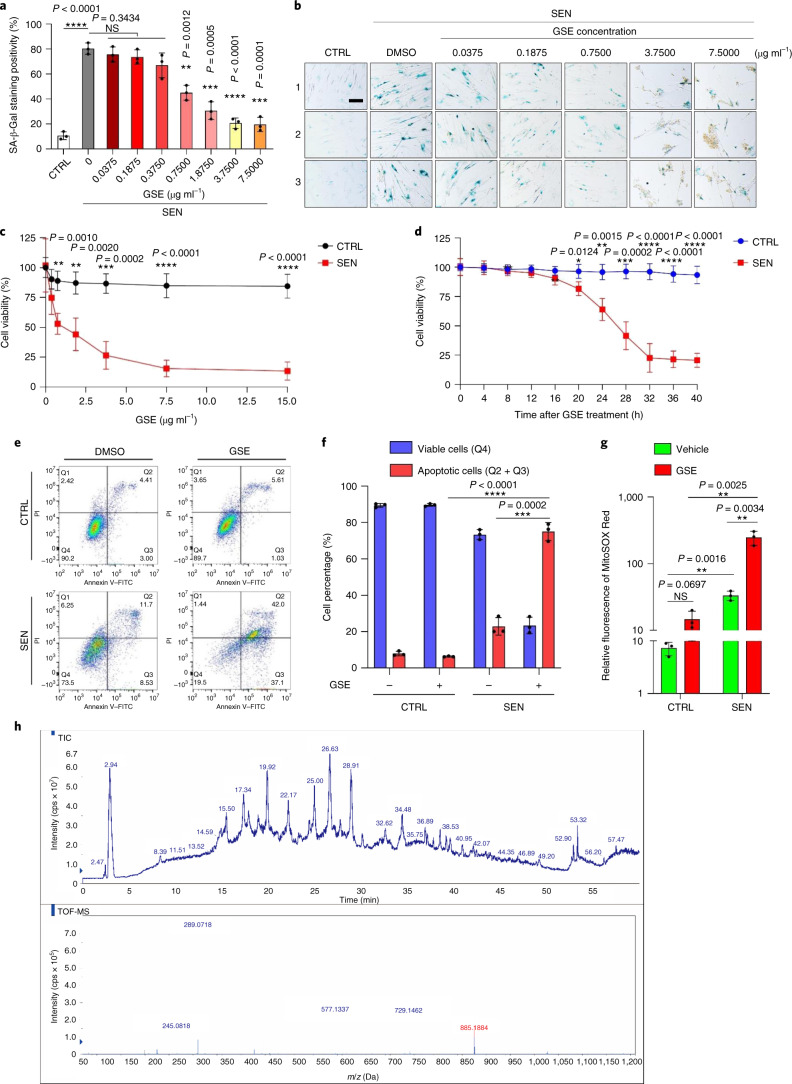
Characterisation of the capacity of GSE to eliminate senescent cells. **a**, Quantification of senescent PSC27 cell survival by SA-β-Gal positivity. GSE was applied to the medium at increasing concentrations. CTRL, control (proliferating) cells; SEN, senescent cells; NS, not significant. *P* values were calculated by one-way ANOVA with Tukey’s multiple-comparison test. **b**, Representative images displaying SA-β-Gal staining after treatment of PSC27 cells with different concentrations of GSE. Scale bar, 20 μm. Data are representative of three independent experiments. DMSO, dimethylsulphoxide. **c**, Survival analysis of control and senescent PSC27 cells upon treatment with GSE (at concentrations of 0.3750, 0.7500, 1.8750, 3.7500, 7.5000 and 15.0000 μg ml^−1^, respectively). Data are shown as mean ± s.d. and were derived from three biological replicates (*n* = 3 independent assays). *P* values were calculated by two-sided *t*-tests. **d**, Time course measurement of in vitro viability upon treatment of control and senescent PSC27 cells with GSE (3.75 μg ml^−1^). Data are shown as mean ± s.d. and were derived from three biological replicates (*n* = 3 independent experiments). *P* values were calculated by two-sided *t*-tests. **e**, Flow cytometry measurement of control and senescent PSC27 cells after processing with an annexin V–FITC and propidium iodide (PI) kit and 4,6-diamidino-2-phenylindole (DAPI) staining to determine the extent of apoptosis. Q1–Q4, quartiles 1–4. **f**, Comparative quantification of the percentage of viable (Q4, PI^−^annexin V^−^) and apoptotic (Q2 and Q3, PI^+^annexin V^+^ and PI^−^annexin V^+^, respectively) cells in control or senescent populations treated with vehicle or GSE for 3 d (*n* = 3 biologically independent assays). *P* values were calculated by two-sided *t*-tests. **g**, Measurement of the fluorescence signal of MitoSOX Red, a mitochondrial superoxide indicator, in PSC27 cells under different conditions. *P* values were calculated by two-sided *t*-tests. **h**, High-resolution mass spectra showing the total ion chromatogram (TIC) and base peak chromatogram of GSE after performing HPLC–ESI-QTOF-MS. Unless otherwise indicated, cells were subjected to relevant analyses 3 d after GSE treatment in the culture condition. cps, counts per second. Data in bar graphs and regression curves are shown as mean ± s.d. and are representative of three biological replicates. NS, *P* > 0.05; *P < 0.05; **P < 0.01; ***P < 0.001; ****P < 0.0001. Source data

Given the efficacy of GSE in reducing the SASP as a senomorphic agent, we next interrogated the potential of this natural product in killing senescent cells at higher concentrations by acting as a senolytic. SA-β-Gal staining indicated that senescent cells were eliminated at a GSE concentration of 0.75 μg ml^−1^ (Fig. 1a,b). At 3.75 μg ml^−1^ GSE, a plateau of 20% senescent cell survival was reached (Fig. 1a,b).

Cell viability assays showed that GSE induced senescent cell death but not proliferating cell death starting from a concentration of 0.75 μg ml^−1^ (Fig. 1c). At a concentration of 7.50 μg ml^−1^, the percentage of surviving senescent cells declined to approximately 10%, whereas the viability of proliferating cells was not affected even at 15.00 μg ml^−1^ GSE (Fig. 1c), the highest concentration used in our cell assays, suggesting prominent selectivity and specificity of GSE against senescent cells, which are major traits of senolytics.

We next measured the capacity of GSE to differentially target senescent cells in a time course. Upon treatment with GSE at a concentration of 3.75 μg ml^−1^, the viability of senescent cells did not significantly decrease until after 20 h. The difference in viability between senescent cells and the control (proliferating cells) reached a maximum after 32 h, implying heterogeneity of intrinsic resistance to senolytics in senescent cell populations (Fig. 1d).

As GSE generates distinct effects against senescent cells, we analysed the efficacy of GSE in inducing cell apoptosis. Flow cytometry demonstrated significantly reduced viability, while apoptosis of senescent cells but not that of proliferating cells was elevated (Fig. 1e,f and Supplementary Fig. 2a). Mitochondrial dysfunction and metabolic changes are among the hallmarks of senescent cells and organismal ageing, events causing oxidative stress and production of reactive oxygen species (ROS) such as superoxide^3,25^. We used MitoSOX Red, a mitochondrial superoxide indicator^26^, to probe intercellular changes and found that GSE promoted the generation of mitochondrial ROS in senescent cells but not in proliferating cells (Fig. 1g). Thus, our data are consistent with a model in which GSE kills senescent cells through induction of apoptosis and exacerbation of mitochondrial stress in vitro.

Grape seeds amount to 38–52%, on a dry matter basis, of grapes and constitute a copious source of antioxidants^27^. We applied high pressure liquid chromatography (HPLC) coupled to quadrupole time-of-flight mass spectrometry (QTOF-MS) equipped with an electrospray ionisation (ESI) interface to identify major components of GSE. We found three major categories of phytochemicals, including phenolic acids, flavonoids (such as flavan-3-ol, procyanidins) and other compounds (Fig. 1h and Supplementary Table 1). Among them, a few components were identified as procyanidins and their derivatives, which were reported to target mitochondrial proteins and alleviate multiple chronic diseases^28^. However, the major component(s) mediating the senolytic function of GSE remains largely unclear.

### The PCC1 component of GSE has senolytic activity

Reported biological activities of grape seed procyanidins include reduction of oxidative damage, suppression of inflammation and induction of cancer cell apoptosis^29–32^. Among individual compounds found in GSE, PCC1 warrants special attention, as it was shown to induce DNA damage, cause cell cycle arrest and increase expression of checkpoint kinases^33^. Data from preliminary analysis (total ion chromatogram) of GSE, a mixture of phytochemical agents per se, by HPLC–QTOF-MS suggested the presence of PCC1, as the profile of GSE at specific MS peaks matched with the chromatogram profile of chemically pure PCC1 acquired from a commercial source (Fig. 1h and Supplementary Fig. 2b).

**Fig. 2:**
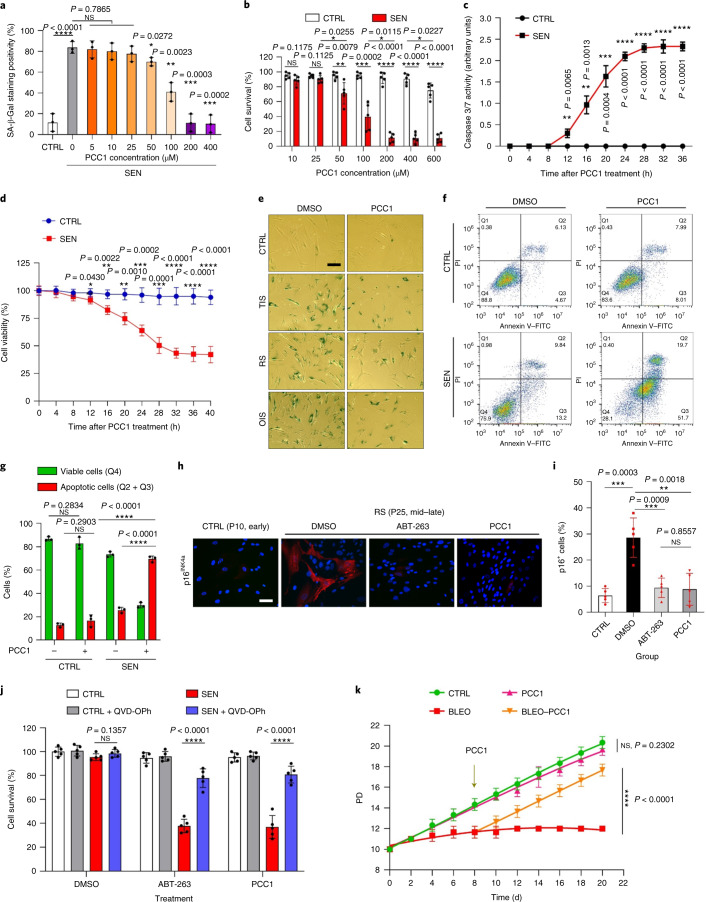
Characterisation of the senolytic potential of PCC1. **a**, Measurement of senescent PSC27 cell survival by SA-β-Gal staining. PCC1 was applied at increasing concentrations. *P* values were calculated by one-way ANOVA with Tukey’s multiple-comparison test. **b**, Survival of senescent PSC27 cells induced by bleomycin at increasing PCC1 concentrations. **c**, Apoptotic assay for caspase 3/7 activity. **d**, Time course survival curves to assess PSC27 cell viability after PCC1 treatment. **e**, Images of SA-β-Gal staining. TIS, therapy-induced senescence (by bleomycin). Scale bar, 20 μm. Data are representative of three independent experiments. **f**, Flow cytometry after processing with an annexin V–FITC and PI kit and DAPI staining to determine apoptosis levels. **g**, Quantification of the percentage of viable (Q4, PI^−^annexin V^−^) and apoptotic (Q2 and Q3, PI^+^annexin V^+^ and PI^−^annexin V^+^, respectively) cells after treatment with vehicle or PCC1 for 3 d (*n* = 3 biologically independent assays). **h**, Immunofluorescence staining of PSC27 cells. RS was induced by serial passaging before PCC1 treatment. Red, p16^INK4a^. Cells at an early passage (P10) were used as a negative control. ABT-263 (1.25 μM) was tested as a positive control. Scale bar, 20 μm. **i**, Statistics of immunofluorescence staining. **j**, PCC1-induced senolytic activity after pan-caspase inhibition (20 μM QVD-OPh). **k**, PD assay of human MSCs. PCC1 was applied on the 8th day after the beginning of experiments as indicated. BLEO, bleomycin. For **c**,**d**,**k**, data are shown as mean ± s.d. and were derived from three biological replicates (*n* = 3 independent assays). For data in **b**–**d**,**g**,**i**,**j**, *P* values were calculated by two-sided *t*-tests. In experiments for **c**–**k**, PCC1 was used at 100 μM. Unless otherwise indicated, samples were collected for analyses 3 d after PCC1 treatment. Data in bar graphs are shown as mean ± s.d. and are representative of three biological replicates. NS, *P* > 0.05; *P < 0.05; **P < 0.01; ***P < 0.001; ****P < 0.0001. Source data

**Extended Data Fig. 4:**
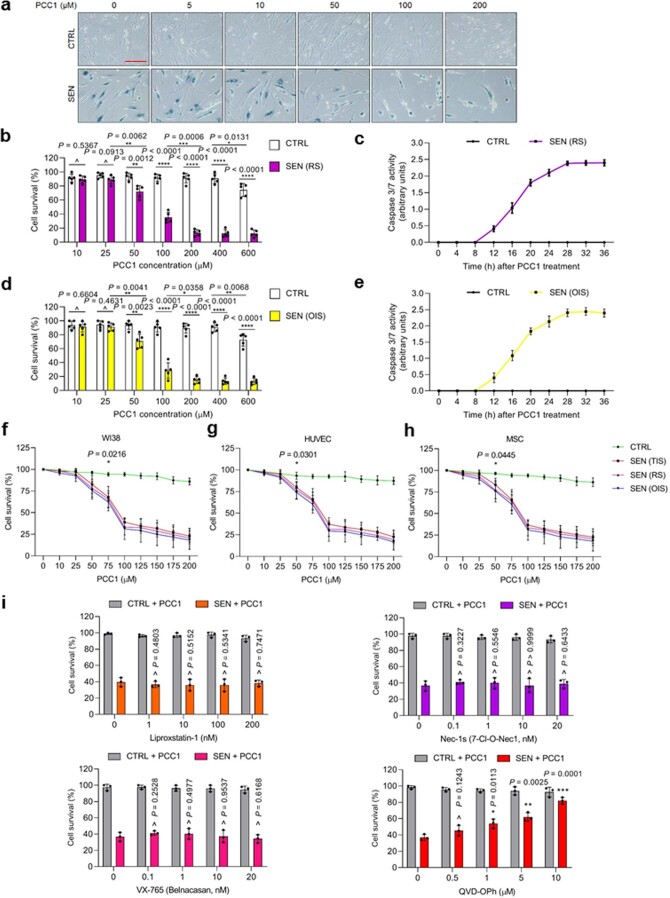
*In vitro* cell assays identify PCC1 as a broad-spectrum senolytic. (**a**) Representative images displaying SA-β-Gal staining results after treatment of PSC27 cells with increasing concentrations of PCC1. Scale bar, 30 μm. Data are representative of 3 independent experiments. (**b**) Senolytic activity appraisal by measuring the percentage of surviving senescent PSC27 cells induced by replicative exhaustion (RS) at increasing PCC1 concentrations. (**c**) Caspase 3/7 activity-based apoptotic evaluation of CTRL and RS cells treated by PCC1. (**d**) Senolytic activity assessment by determining the percentage of surviving senescent cells induced by oncogenic HRas^G12V^ (OIS) at increasing PCC1 concentrations. For **b** and **d**, *P* values were calculated by two-sided *t*-test. (**e**) Caspase 3/7 activity-based apoptotic appraisal of CTRL and OIS cells treated by PCC1. (**f**-**h**) Quantification of the viability of cells in CTRL, TIS, RS and OIS groups treated by 100 μM PCC1 for 3 d. (**f**), WI38. (**g**), HUVEC. (**h**), MSC. The significance of *P* values for the first concentration of PCC1 that differentiating the survival of CTRL and SEN cells was marked *per* cell line-based assay. For **f**, **g** and **h**, *P* values were calculated by one-way ANOVA with Tukey’s multiple-comparison test. (**i**) Quantification of cell survival in CTRL and SEN populations after combined treatment of 100 μM PCC1 with a ferroptosis inhibitor (liproxstatin-1), necroptosis inhibitor (Nec-1s), caspase-1 inhibitor (VX-765), or a pan-caspase inhibitor (QVD-O-Ph). Cell survival was presented as comparative data of different concentrations of chemicals relative to cells treated by the vehicle. *P* values were calculated by two-sided *t*-test. All data are shown as mean ± SD, and are representative of 3 biological replicates. ^, *P* > 0.05. *, *P* < 0.05. **, *P* < 0.01. ***, *P* < 0.001. ****, *P* < 0.0001. Source data

PCC1 was shown to decrease the level of BCL-2 but increase expression of the regulator BAX and activities of caspases 3 and 9 in cultured cancer cells, thus potentially generating anticancer effects through induction of apoptosis^33^. Hence, we next assessed the capacity and selectivity of PCC1 to eliminate senescent cells in culture. The data suggest that PCC1 is senolytic for senescent stromal cells starting at a concentration of 50 μM, at which proliferating cells remain largely unaffected (Fig. 2a,b and Supplementary Table 2). Although higher concentrations caused a lower survival rate of senescent cells, with a threshold approximately at 200 μM, PCC1 only exhibited toxicity toward control cells when used at 600 μM or higher (Fig. 2b). A time course of caspase 3/7 activity indicated that PCC1 exerted apoptotic effects within 12 h, reaching a plateau at 24 h (Fig. 2c). This finding was largely consistent with viability measurements (Fig. 2d). The senolytic nature of PCC1 was confirmed by cells that entered senescence due to replicative exhaustion or senescence (RS) or oncogene (HRAS^G12V^) overexpression (OIS), which generates stressful insults similar to those of therapy-induced senescence (Fig. 2e, Extended Data Fig. 4b–e and Supplementary Table 2). Together, the results suggest that PCC1 selectively clears senescent human stromal cells induced by different stimuli in a dose-dependent manner but without a significant effect on nonsenescent cells when used at appropriate concentrations.

To experimentally expand and establish PCC1 efficacy across cell lineages, we treated human foetal lung fibroblasts (WI38), primary human umbilical vein endothelial cells (HUVECs) and human mesenchymal stem cells (MSCs) with PCC1 and found that senescent cells of all these lineages exhibited similar susceptibility for selective ablation by PCC1, whereas their nonsenescent counterparts remained viable (Extended Data Fig. 4f–h and Supplementary Table 3). We further confirmed the induction of apoptosis in senescent cells in response to PCC1 by flow cytometry, whereas proliferating cells remained largely unaffected by PCC1 (Fig. 2f,g). In sum, our data show that PCC1 selectively eliminates senescent cells across various cell types and arising from different triggers of senescence.

To visualize the depletion of senescent cells by PCC1, we examined expression of p16^INK4a^, a widely used marker of senescence, in stromal cells that experienced RS. PCC1 effectively removed p16-positive senescent cells, which only appeared in late-passage PSC27 populations, with an efficacy largely resembling that of ABT-263 (1.25 μM), a well-established synthetic senolytic agent^18,21^ (Fig. 2h,i).

To substantiate that PCC1-mediated elimination of senescent cells occurs mainly through induction of apoptosis, rather than through other forms of programmed cell death, we treated cells with the pan-caspase apoptosis inhibitor quinolyl-valyl-*O*-methylaspartyl-(-2,6-difluorophenoxy)-methylketone (QVD-OPh). The ability of PCC1 to kill senescent cells was reversed by QVD-OPh. PCC1 thus shares its caspase-dependent induction of apoptosis as a senolytic feature with ABT-263 (Fig. 2j). Further analysis with chemical inhibitors excluded PCC1-induced cell death through ferroptosis, pyroptosis or necroptosis (Extended Data Fig. 4i).

To assess the potential of cell population doubling (PD) after treatment with genotoxic drugs, we employed MSCs, which can self-renew and resume colony-based proliferation even after exposure to environmental stresses^34^, likely due to the heterogeneity of damage, with cells experiencing less damage presumably able to retain the potential to self-recover and re-enter the cell cycle^24,35^. Unlike bleomycin-damaged cells, which rapidly entered growth arrest after treatment, post-senescence treatment with PCC1 significantly enhanced the PD capacity of MSCs, especially after removal of senescent cells developing the SASP and holding the potential to induce paracrine senescence within cell populations (Fig. 2k). However, treatment with PCC1 did not affect the PD of proliferating cells, further indicative of the selectivity of PCC1 for senescent cells compared with their normal counterparts.

As GSE is a complex phytochemical mixture, with many of its components having reported antioxidant and anti-inflammatory activities^27,36^, we investigated whether PCC1 was the principal constituent of GSE involved in depleting senescent cells or whether alternative phytochemicals in GSE could contribute to its overall senolytic effect. To this end, we examined the influence of individual phytochemical molecules on the survival of senescent PSC27 cells. Most GSE components failed to display senolytic activity in the dose range of PCC1 and did not cause significant death of proliferating cells (Supplementary Figs. 3 and 4). Although the flavonoid quercetin showed senolytic activity as in our previous studies, a property shared with natural flavones^11,21^, ‘reconstituted GSE’, consisting of the major components mixed according to their mass percentage as revealed by our HPLC–QTOF-MS data (Supplementary Table 1, note that quercetin accounts for only 0.9%) but purposefully excluding PCC1, did not show similar results as those observed for PCC1 in both assays (Supplementary Figs. 3 and 4). Although we cannot conclude whether other components have a contribution, our data clearly suggest that PCC1 is a primary mediator of the senolytic effect of GSE.

### PCC1 induces mitochondrial dysfunction in senescent cells

**Fig. 3:**
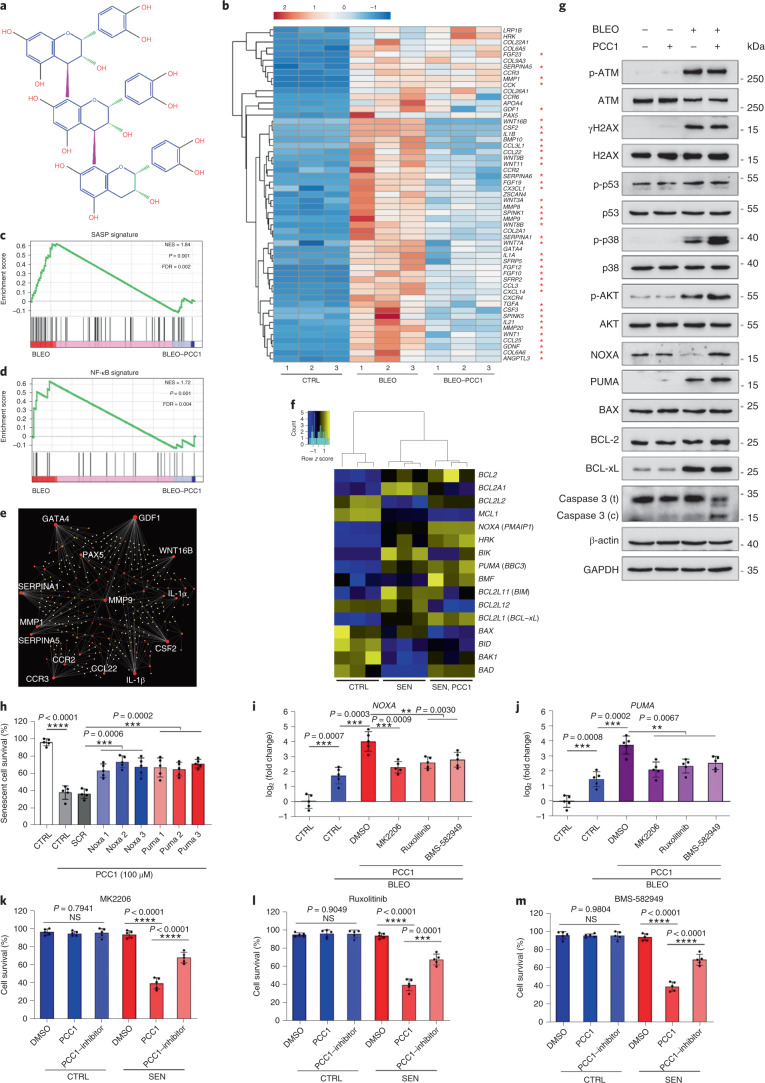
PCC1 induces senescent cell apoptosis by engaging pro-apoptotic pathways. **a**, Chemical structure of the trimeric epicatechin PCC1. **b**, Heatmap depicting top genes (50) significantly upregulated in senescent PSC27 cells but downregulated upon PCC1 treatment (50 μM). Red stars, SASP factors. **c**, GSEA plot of a significant gene set in the SASP spectrum. FDR, false discovery rate; NES, normalized enrichment score. **d**, GSEA plot of a significant gene set associated with NF-κB-mediated signalling. **e**, NetworkAnalyst map of protein–protein interactions of typical SASP-associated factors significantly upregulated in senescent cells but downregulated by PCC1 treatment. **f**, Heatmap showing the differential expression of BCL-2 family genes in control, senescent and PCC1-treated senescent cells. **g**, Immunoblot of PSC27 cells exposed to different agents. Expression of pro-apoptotic and anti-apoptotic factors and DDR signalling-associated molecules was examined. Caspase 3 (t), total caspase 3; caspase 3 (c), cleaved caspase 3; p, phosphorylated. β-actin and GAPDH, loading controls. Data are representative of three independent experiments. **h**, Cells were infected with three different short hairpin RNA species targeting *NOXA* or *PUMA* before being exposed to bleomycin to induce senescence. Seven days later, cells were treated with PCC1 (100 μM) for a 3-d period to induce apoptosis. SCR, scramble. **i**, *NOXA* expression was determined using quantitative PCR with reverse transcription (RT–qPCR). Cells were treated with bleomycin to induce senescence before exposure to 100 μM PCC1 for 3 d in the absence or presence of 10 μM MK2206, 10 μM ruxolitinib or 20 nM BMS-582949 to inhibit the activity of AKT, JAK1 and/or JAK2 or p38 MAPK, respectively. **j**, A similar set of RT–qPCR expression assays for *PUMA* using conditions described in **i**. **k**–**m**, Measurement of cell viability after PCC1 treatment in the absence or presence of MK2206 (**k**), ruxolitinib (**l**) or BMS-582949 (**m**), included to inhibit the enzymatic activity of AKT, JAK1 and/or JAK2 or p38 MAPK, respectively. For data in **c**,**d**, *P* values were calculated by one-way ANOVA with Tukey’s post hoc comparison. Statistical significance in **h**–**m** was calculated using two-sided *t*-tests or one-way ANOVA (Dunnett’s test). Data in all bar graphs are shown as mean ± s.d. and represent three biological replicates. NS, *P* > 0.05; *P < 0.05; **P < 0.01; ***P < 0.001; ****P < 0.0001. Source data

Given the prominent efficacy of PCC1 in selectively inducing senescent cell death, we interrogated the underlying mechanism(s). PCC1 belongs to the superfamily of flavonoids, which can scavenge free radicals, chelate metals and reduce hydroperoxide formation, antioxidant properties attributable to the functional ‘-OH’ group in the structure and its position on the ring of the flavonoid molecule^27^. The antioxidant capacity of procyanidins is, in part, governed by their degree of polymerisation, while PCC1 is a procyanidin epicatechin trimer by nature (Fig. 3a).

We first analysed the impact of PCC1 on the transcriptome-wide expression of senescent cells. Bioinformatics showed that 4,406 genes were significantly upregulated and 2,766 genes were downregulated in stromal cells after PCC1 treatment (Supplementary Fig. 5a). We observed a large array of SASP factors, expression of which was markedly upregulated during cellular senescence but substantially downregulated when senescent cells were exposed to PCC1 (Fig. 3b). GSEA profiling showed that both the SASP and NF-κB signatures were remarkably suppressed by PCC1 treatment (Fig. 3c,d). We further noticed multiple mutual interactions or functional connections between these factors upregulated during senescence and downregulated after PCC1 treatment appearing in the list of top differentially expressed genes, most of which were typically secreted factors (Fig. 3e).

To understand the selectivity of PCC1 for senescent cells, we further assessed the transcriptomic expression profile and noticed that PCC1 induced expression changes in a few BCL-2 superfamily members (Fig. 3f). Although DDR signalling remained largely unaffected, PCC1-dependent upregulation or activation of p38 mitogen-activated protein kinase (MAPK) was observed, with caspase 3 cleavage occurring in these cells (Fig. 3g). Although BCL-xL expression was elevated in senescent cells relative to that in their proliferating controls, PCC1 treatment did not further enhance its protein level. Levels of the other two BCL-2 factors, namely, BCL-2 and BAX, remained largely unchanged. While NOXA and PUMA (two members of the BCL-2 homology domain 3 (BH3)-only pro-apoptotic subfamily) exhibited different expression patterns during cellular senescence, PCC1 treatment resulted in the upregulation of both factors (Fig. 3g).

**Extended Data Fig. 5:**
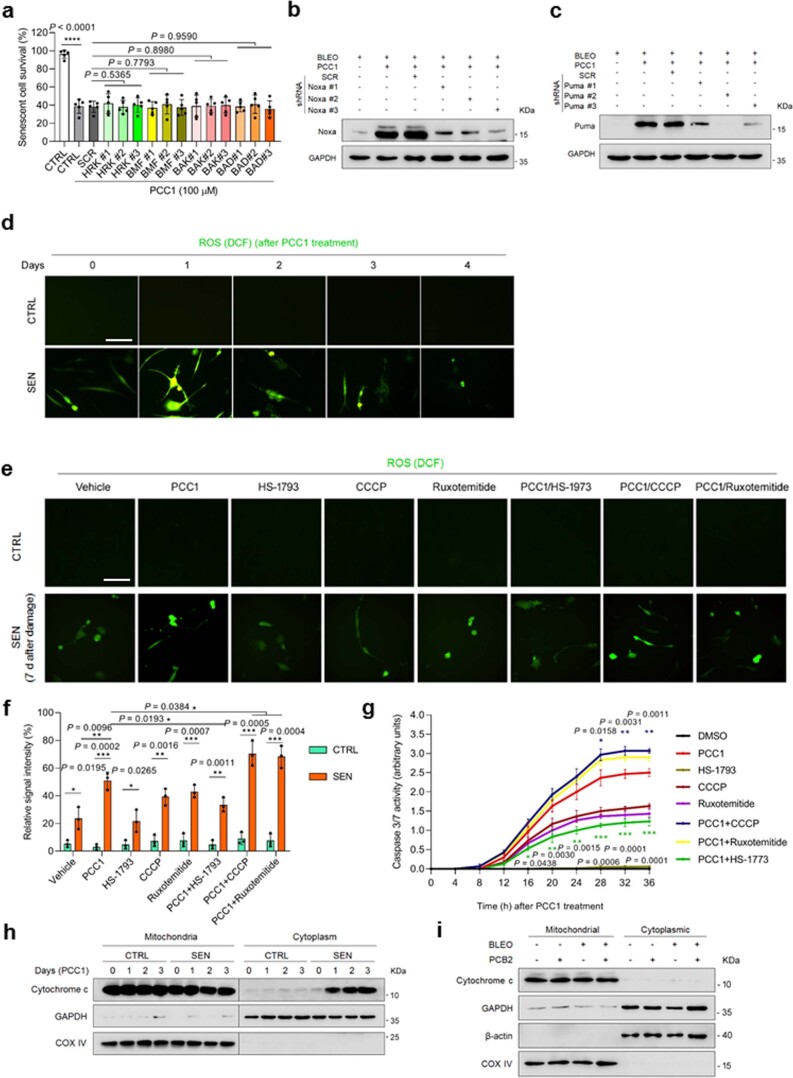
Characterization of PCC1-induced apoptosis of senescent cells. (**a**) Survival assays of PSC27 cells infected with lentivirus encoding shRNAs against indicated factors. Cells were exposed to PCC1 (100 μM) 7 days after BLEO-caused senescence, for a 3-d period to induce apoptosis. SCR, scramble. *P* values were calculated by one-way ANOVA with Tukey’s multiple-comparison test. (**b**) Immunoblot profiling Noxa expression in PSC27 cells infected with lentivirus encoding shRNAs specific for Noxa before treatment with different agents. (**c**) Immunoblot profiling Puma expression in PSC27 cells, in a workflow resembling that of Noxa. (**d**) Staining of ROS in senescent PSC27 cells with 2’-7’-dichlorodihydrofluorescein diacetate (DCFH-DA), a cell permeable fluorescent probe indicative of changes in redox state. Signals were measured in a 4-d time course starting from PCC treatment. Scale bar, 20 μm. (**e**) Examination of ROS by DCFH-DA-staining. Cells were exposed to different agents 7 days after BLEO challenge, for a 3-day period. Staining with DCFH-DA was performed 1 day after addition of individual agents. Scale bar, 20 μm. (**f**) Statistical comparison of ROS signal intensity measured for cells described in (**e**). (**g**) Apoptotic assay of PSC27 cells exposed to *in vitro* treatments described in (**e**) by examination of caspase 3/7 activity. Signals from DMSO treatment as a baseline, with indicated *P* values derived from comparison against PCC1-treated samples. Data are shown as mean ± SD and derive from 3 biological replicates (*n* = 3 independent assays). *P* values were calculated by two-sided *t*-test. (**h**) Immunoblot of cytochrome c in cells exposed to PCC1. (**i**) Immunoblot of cytochrome c in cells exposed to PCB2. For (**h**) and (**i**), distribution of cytochrome c between mitochondria and cytoplasm was profiled by isolating mitochondria from cytosol supernatants at specialized timepoints (day 0-3 for **h**, and 3 for **i**) after procyanidin treatment. COX IV, mitochondrial marker. Data of **b**-**d**, **h**-**i** are representative of 3 independent experiments. Data of bar graphs (a and **f**) are shown as mean ± SD and represent 3 biological replicates. ^, *P* > 0.05. *, *P* < 0.05. **, *P* < 0.01. ***, *P* < 0.001. ****, *P* < 0.0001. Source data

Knockdown of BCL-2 pro-apoptotic factors suggested that NOXA and PUMA partially mediated the senolytic actions of PCC1 (Fig. 3h and Extended Data Fig. 5a–c). Treatment with chemical inhibitors of AKT kinase, Janus kinase (JAK)1, JAK2 and p38 MAPK signalling also suggested involvement of these signalling pathways in expression of *PMAIP1* (*NOXA*) and *BBC3* (*PUMA*) and senescent cell apoptosis after PCC1 treatment (Fig. 3i–m).

**Fig. 4:**
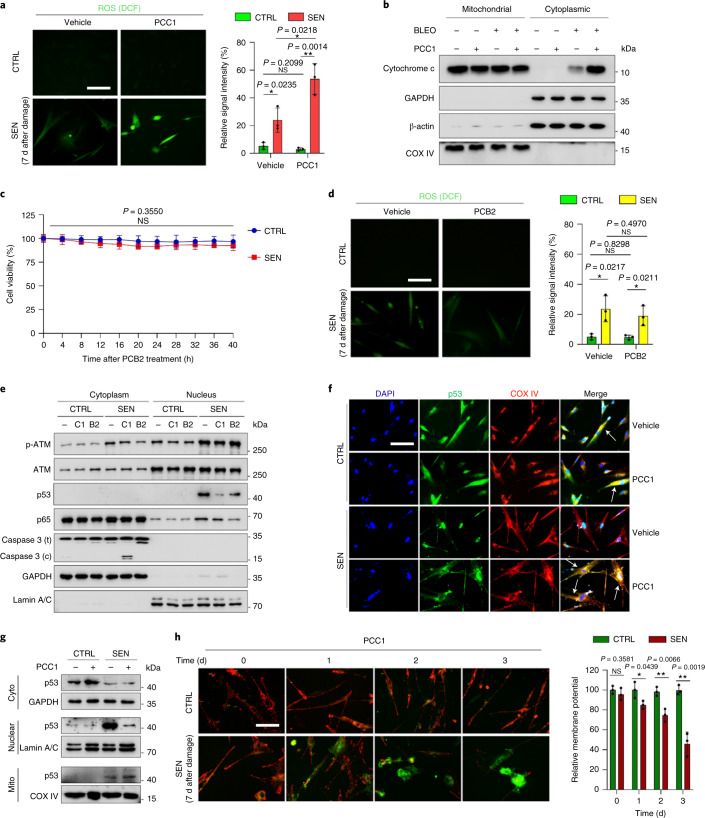
PCC1-induced apoptosis is partially mediated through mitochondrial dysfunction. **a**, Measurement of ROS levels with DCFH-DA, a cell-permeable fluorescent probe sensitive to changes in cellular redox state. Experiments were performed 1 d after PCC1 treatment. Left, representative images. Scale bar, 10 μm. Right, statistics. DCF, dichlorodihydrofluorescein. **b**, Immunoblot after exposure of cells to different treatments. Cytochrome c distribution between mitochondria and the cytoplasm was profiled by isolating mitochondria from cytosol supernatants 3 d after PCC1 treatment. COX IV is the terminal enzyme of the mitochondrial respiratory chain and a mitochondrial marker. **c**, Time course survival curves to assess PSC27 cell viability upon treatment with PCB2, another member of the natural procyanidin family. Data are shown as mean ± s.d. and were derived from three biological replicates (*n* = 3 independent assays). **d**, ROS-production assay performed in a similar manner as described in **a**, except that cells were exposed to PCB2. Scale bar, 10 μm. **e**, Immunoblot of the expression and distribution of ATM, p53 and caspase 3 between the cytoplasm and the nucleus. GAPDH and lamin A/C, loading controls for cytoplasm and nuclei, respectively. C1, PCC1; B2, PCB2. **f**, Confocal microscopy of immunofluorescence staining after treatment of cells with vehicle (DMSO) or PCC1. Primary antibodies specific to p53 or COX IV were applied. Scale bar, 10 μm. **g**, Immunoblot analysis of PSC27 cells exposed to different agents. Cyto, cytoplasmic; mito, mitochondrial. **h**, Analysis of JC-1 staining, a fluorescent probe indicative of Δψm. Signals were measured over 3 d. Green fluorescence indicates JC-1 monomers (they appear in the cytosol after mitochondrial membrane depolarisation and indicate early-stage apoptosis). Red fluorescence indicates JC-1 aggregation (resides on intact mitochondria). Left, representative images. Right, statistics. Both PCC1 and PCB2 were used at 100 μM in relevant assays. Data in **b**,**e**–**g** are representative of three independent experiments. Statistical significance in **a** (right), **d** (right) and **h** (right) was calculated using two-sided *t*-tests, and that in **c** was calculated with one-way ANOVA (Dunnett’s test). Data in all bar graphs are shown as mean ± s.d. and are representative of three biological replicates. NS, *P* > 0.05; *P < 0.05; **P < 0.01. Source data

As knocking down *NOXA* and *PUMA* only partially inhibited the senolytic effect of PCC1 (Fig. 3h,k–m), we investigated other possible mechanism(s) leading to senescent cell death. Because procyanidins usually increase cell viability, decrease ROS production and restrain oxidative stress in mammalian cells^37,38^, we next asked whether similar or antioxidant effects could be observed in senescent cells exposed to PCC1. Surprisingly, we found that the opposite was the case, as senescent PSC27 cells displayed elevated ROS levels when treated with PCC1, in contrast to their proliferating counterparts (Fig. 4a and Extended Data Fig. 5d, note signals from the 2′-7′-dichlorodihydrofluorescein diacetate (DCFH-DA) probe). Treatment with HS-1793, a stable resveratrol analogue that has free radical-scavenging activity^39^, effectively blocked ROS production in senescent cells treated with PCC1 (Extended Data Fig. 5e,f), whereas ROS levels were further increased after exposure of PCC1-treated senescent cells to CCCP, a protonophore mitochondrial uncoupler^40^, or ruxotemitide (LTX-315), an amphipathic cationic peptide that induces permeabilisation of the outer mitochondrial membrane^41^, each applied at concentrations that were not cytotoxic to control cells (Extended Data Fig. 5e,f). Although treatment with either CCCP or ruxotemitide per se also caused enhanced ROS production, the effects were generally smaller than those induced by PCC1, suggesting that PCC1 triggers mitochondrial dysfunction in senescent cells. By measuring the apoptotic index of senescent cells (caspase 3/7 activity), we found that the PCC1-induced effect could be further enhanced upon combination of PCC1 with each mitochondrial disruptor but suppressed upon co-treatment with HS-1793 (Extended Data Fig. 5g).

Cytochrome c release and mitochondrial membrane disruption are intracellular events associated with apoptosis and often act as direct apoptotic drivers^42^. Our data suggest that PCC1 treatment enhanced cytochrome c release from mitochondria to the surrounding cytoplasmic space (Fig. 4b and Extended Data Fig. 5h). The release of cytochrome c from mitochondria is largely consistent with biochemical reactions such as caspase activation in PCC1-treated senescent cells (Fig. 3g).

Members of the procyanidin family exhibit a broad spectrum of pharmacological properties including anti-oxidation and anti-inflammation, which are the opposite of what we observed when treating senescent cells with PCC1. The current data prompted us to reason whether the effects of PCC1 are reproduced by other procyanidins. Procyanidin B2 (PCB2) is a representative flavonoid that exists as a dimer and reduces ROS levels during oxidative stress in cultured cells^43^. PCB2 failed to eliminate senescent cells (Fig. 4c and Supplementary Figs. 3b and 4b) and neither enhanced ROS production nor induced mitochondrial release of cytochrome c in senescent cells (Fig. 4d and Extended Data Fig. 5i). A substantial amount of p65 (RelA), one of the major subunit of the NF-κB complex, translocated to the nucleus of senescent cells (Fig. 4e). Although PCB2 treatment counteracted p65 nuclear translocation, which is consistent with its anti-inflammatory capacity, this effect was not reproduced by PCC1 (Fig. 4e). Senescent cells exposed to PCC1 exhibited remarkable caspase 3 cleavage, whereas those treated with PCB2 did not, further differentiating the biological activity of these two procyanidin molecules (Fig. 4e).

As a factor that functionally governs cell fate, p53 can induce apoptosis either by transactivating pro-apoptotic genes or in a transcription-independent manner by translocating to mitochondria^44^. We observed increased nuclear translocation of p53 upon cellular senescence, a pattern markedly reduced by PCC1, but much less so than that by PCB2 (Fig. 4e,f). As nuclear exclusion of p53 is a critical step in the induction of senescent cell apoptosis^45^, we further assessed the distribution of p53. Immunofluorescence staining indicated substantially increased overlap of p53 with cytochrome c oxidase subunit IV (COX IV) (a transmembrane protein complex in the mitochondrial respiratory electron chain, often used as a mitochondrial resident protein marker) in PCC1-treated senescent cells, suggesting enhanced translocation of p53 into the mitochondrial matrix. Although we observed some p53 in mitochondria of proliferating cells, PCC1 did not induce a remarkable or comprehensive influx of p53 protein into the mitochondrial matrix of proliferating cells (Fig. 4f). However, in senescent cells, p53 levels were decreased in the nuclei but increased in mitochondria upon exposure to PCC1 (Fig. 4g).

Mitochondrial membrane potential (Δψm) decline is an event that can trigger apoptosis through the mitochondrial-mediated intrinsic pathway^46^. We found that Δψm was significantly reduced in senescent cells, while proliferating cells remained basically unaffected in the presence of PCC1, as indicated by the profile of JC-1 probe signals (Fig. 4h). Thus, PCC1 promotes ROS generation, triggers cytochrome c release and causes Δψm disturbance in senescent cells, events inherently associated with mitochondrial disability and functionally driving cell apoptosis.

Together, our experimental data suggest that senescent cells are subject to PCC1-induced apoptosis, a process partially mediated by *NOXA* and *PUMA* upregulation and associated with enhanced ROS production and mitochondrial dysfunction.

### PCC1 promotes tumour regression and reduces chemoresistance

Given the capacity and selectivity of PCC1 for eliminating senescent cells in vitro, we next interrogated whether this agent could be exploited to intervene against age-related pathologies in vivo. In clinical oncology, drug resistance limits the efficacy of most anticancer treatments, while senescent cells frequently contribute to therapeutic resistance through development of an in vivo SASP in the drug-damaged tumour microenvironment (TME)^15,16,47^. Pharmacological elimination of therapy-induced senescent cells minimizes side effects of chemotherapy and prevents cancer recurrence in animals^48^. However, the feasibility of PCC1-mediated depletion of senescent cells from primary tumours to enhance the efficacy of anticancer treatments remains largely unknown.

**Extended Data Fig. 6:**
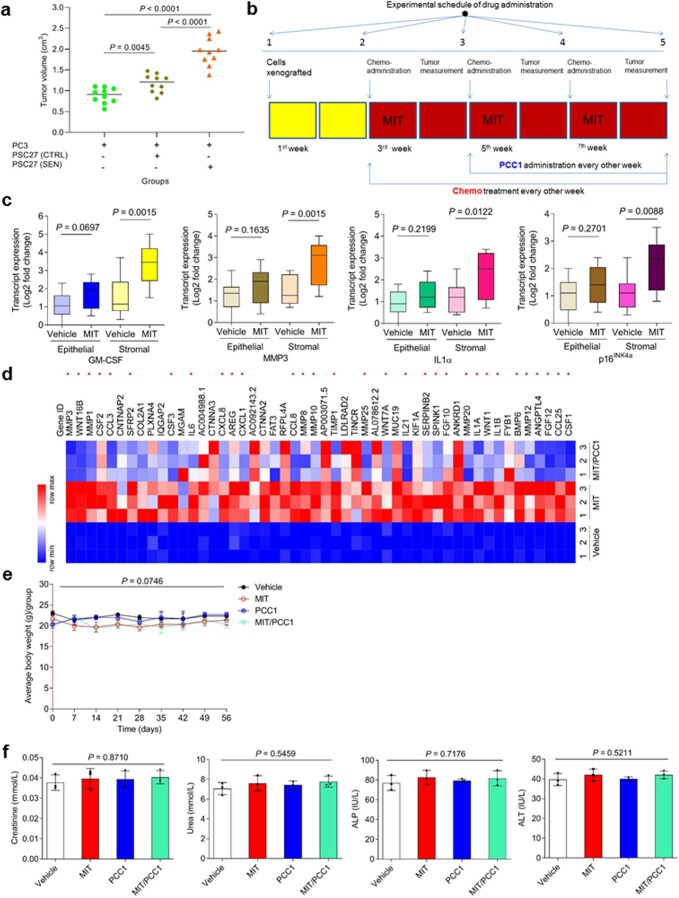
Schematic design of preclinical trial, expression analysis of the SASP and pathophysiological appraisal of treatments. (**a**) Statistics of tumor end volumes. PC3 cells were xenografted alone or together with PSC27 to the hind flank of animals, with tumor volumes measured at the end of an 8-week period. (**b**) Cancer cells (PC3) alone or alongside stromal cells (PSC27) were inoculated subcutaneously to NOD/SCID males 2 weeks before chemotherapy. MIT was delivered via intravenous injection on the 1^st^ day of each week starting from the 3^rd^ week, then given every other week, totally 3 doses. PCC1 administered via *i.p*. starting from beginning of the 5^th^ week, then once *per* 2 weeks. At the end of 8 weeks mice were sacrificed, tumor volume measured and tissues assessed. (**c**) Transcript analysis of canonical SASP factors (GM-CSF/MMP3/IL1α) and p16^INK4a^ expressed in epithelial and stromal cells, respectively. Individual cell types isolated from tumor tissues via LCM. Data are representative of 3 biological replicates (*n* = 10 animals per group). Datasets are displayed as box-and-whisker plots, where a box extends from the 25th to 75th percentile with the median shown as a line in the middle, with whiskers indicating smallest and largest values. *P* values were calculated by two-sided *t*-test. (**d**) Heatmap depicting top genes (50) significantly upregulated in PSC27 xenografted subcutaneously to mice and induced senescent by MIT. Gene expression in animals experiencing MIT/PCC1 co-treatment also profiled, with transcript signals normalized to vehicle-treated group. Red stars, SASP factors. (**e**) Body weight determination performed on a weekly basis for immunodeficient mice. (**f**) Serum measurement of creatinine, urea, alkaline phosphatase (ALP), and alanine aminotransferase (ALT) with terminal bleeds (cardiac punctures) taken at the end of therapeutic regimens. For a and **c**, *P* values were calculated by two-sided *t*-test. For **e**-**f**, *P* values were calculated by one-way ANOVA with Tukey’s multiple-comparison test. Data in **a**, **e** and **f** are shown as mean ± SD and representative of 3 independent experiments. MIT, mitoxantrone. For each dataset, *n* = 10 *per* treatment arm. Source data

First, we chose to build tissue recombinants by admixing PSC27 cells with PC3 cells, which are a typical prostate cancer cell line of high malignancy, at a pre-optimized ratio (1:4)^14^. The cells were then subcutaneously implanted into the hind flank of mice with non-obese diabetes and severe combined immunodeficiency (NOD–SCID). Tumours of animals were measured at the end of an 8-week period, and tissues were acquired for pathological appraisal. Compared to tumours comprising PC3 cancer cells and naive PSC27 stromal cells, xenografts composed of PC3 cells and senescent PSC27 cells exhibited significantly increased volume, confirming the tumour growth-promoting effects of senescent cells (Extended Data Fig. 6a).

**Fig. 5:**
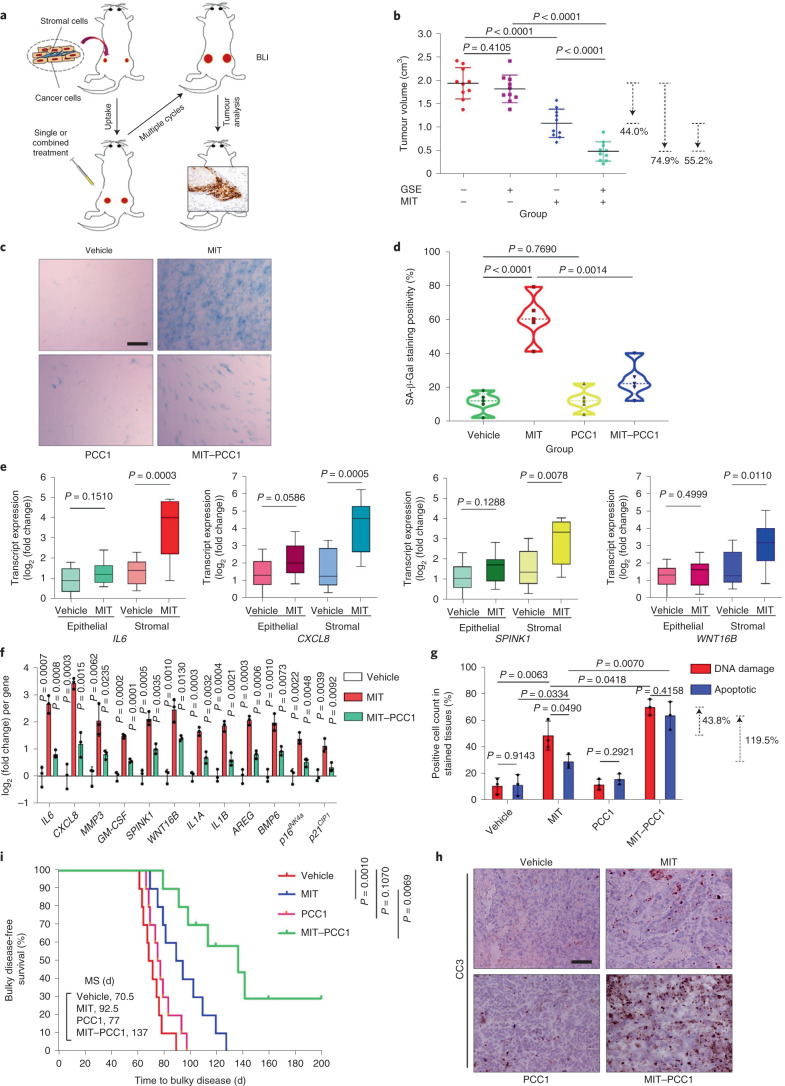
Senolysis by PCC1 in the damaged TME diminishes SASP-conferred cancer resistance. **a**, Illustrative diagram of a preclinical regimen. Two weeks after subcutaneous implantation and in vivo uptake of tissue recombinants, NOD–SCID male mice received either single (mono) or combined (dual) agents in a metronomic schedule composed of several cycles. BLI, bioluminescence imaging. **b**, Statistical profiling of tumour end volumes. PC3 cells were xenografted alone or together with PSC27 cells into the hind flank of animals. **c**, Comparative evaluation of in vivo senescence by SA-β-Gal staining. Tumours were freshly dissected after killing animals and processed as frozen sections for histological staining. Scale bars, 200 μm. **d**, Violin plots depicting comparative statistics of SA-β-Gal staining in tumour tissues. **e**, Transcript assay for in vivo expression of several canonical SASP factors in stromal cells isolated from tumours. Tissues from animals xenografted with both stromal and cancer cells were subjected to laser capture microdissection-supported isolation and subsequent processes. Data are representative of three biological replicates (*n* = 10 animals per group). Datasets are displayed as box-and-whisker plots, in which a box extends from the 25th to the 75th percentile with the median shown as a line in the middle and whiskers indicating smallest and largest values. **f**, Profiling of SASP transcripts in stromal cells. Signals corresponding to each factor were normalized to those from the vehicle-treated group. Note *p16*^*INK4a*^ is also known as *CDKN2A* and *p21*^*CIP1*^ is also known as *CDKN1A*. **g**, Statistical measurement of cells with DNA damage and apoptotic cells in biospecimens collected as described in **a**,**b**. Values are presented as the percentage of cells positively stained by immunohistochemistry (IHC) with antibodies specific to histone γH2AX or caspase 3 (cleaved). For **b**,**d**–**g**, *P* values were calculated by two-sided *t*-tests. **h**, Representative IHC images of caspase 3 (cleaved, CC3) at the end of the therapeutic regimes. Scale bars, 100 μm. **i**, Comparative survival of mice killed after the development of advanced bulky diseases. Survival duration was calculated from the time of recombinant tissue injection until animal death. MS, median survival. *P* values were calculated by two-sided log-rank (Mantel–Cox) tests. Data in **c**,**h** are representative of three independent experiments. Data in all bar graphs are shown as mean ± s.d. and are representative of three biological replicates. Source data

To mimic clinical conditions, we experimentally designed a preclinical regimen incorporating genotoxic therapeutics and/or senolytics (Fig. 5a). Two weeks after subcutaneous implantation, when stable uptake of tumours in vivo was observed, a single dose of mitoxantrone (MIT, a chemotherapeutic drug) or placebo was delivered to animals on the 1st day of the 3rd, 5th and 7th weeks until the end of the 8-week regimen (Extended Data Fig. 6b). In contrast to the placebo-treated group, MIT administration remarkably delayed tumour growth, validating the efficacy of MIT as a chemotherapeutic agent (44.0% reduction in tumour size) (Fig. 5b). Notably, although administration of PCC1 itself did not cause tumour shrinkage, treatment with MIT followed by PCC1 delivery (at 20 mg per kg via intraperitoneal (i.p.) injection 2 weeks after the first MIT dose and then delivered biweekly) remarkably enhanced tumour regression (55.2% reduction in tumour size compared with MIT alone; 74.9% reduction in tumour volume compared with the placebo treatment) (Fig. 5b).

We next tested whether cellular senescence occurred in the tumour foci of these animals. Unsurprisingly, MIT administration induced the appearance of a large number of senescent cells in tumour tissue. However, delivery of PCC1 to these chemotherapy-treated animals depleted the majority of senescent cells (Fig. 5c,d). Laser capture microdissection followed by transcript assays indicated significantly increased expression of SASP factors including *IL6*, *CXCL8*, *SPINK1*, *WNT16B* (also known as *WNT16*), *GM-CSF* (also known as *CSF2*), *MMP3* and *IL1A*, a tendency accompanied by upregulation of the gene encoding the senescence marker p16^INK4a^ in chemotherapy-treated animals (Fig. 5e and Extended Data Fig. 6c). These changes were mainly observed in stromal cells, rather than in neighbouring cancer cells, implying the possibility of repopulation of residual cancer cells, which frequently develop acquired resistance in the treatment-damaged TME. However, upon administration of PCC1, SASP-associated changes were largely reversed, as suggested by transcript assays and RNA-seq (Fig. 5f and Extended Data Fig. 6d).

To investigate the mechanisms underlying SASP expression in MIT-treated mice, we dissected tumours from animals treated with these two agents 7 d after the first dose of GSE delivery, a time point before the development of resistant colonies. In contrast to placebo treatment, MIT administration increased DNA damage and apoptosis, whereas treatment with PCC1 alone did not (Fig. 5g). However, when MIT-treated animals were co-administered PCC1, DNA damage and apoptosis were significantly augmented, implying enhanced cytotoxicity in animals receiving both chemotherapy and senolytics. As supporting evidence, we observed elevated caspase 3 cleavage, a typical hallmark of cellular apoptosis, when PCC1 was administered alongside MIT (Fig. 5h).

We next evaluated the consequences of tumour progression by comparing the survival of different animal groups over time. In this preclinical cohort, animals were monitored for tumour growth, with bulky disease considered to have arisen once the tumour burden was prominent (size ≥ 2,000 mm^3^), an approach employed in former studies^14,49^. Mice receiving the MIT–PCC1 combinatorial treatment showed the most prolonged median survival, surviving at least 48.1% longer than the group treated with MIT alone (Fig. 5i, green versus blue). However, PCC1 treatment alone only marginally extended survival. Our data suggest that PCC1 administration alone neither changes tumour growth nor promotes animal survival, whereas co-administration of PCC1 with MIT has significant synergistic effects.

Of note, treatments performed in these studies appeared to be well tolerated by animals, as no significant perturbations in urea, creatinine or liver enzyme levels or body weight were observed (Extended Data Fig. 6e,f). More importantly, chemotherapeutic and geroprotective agents administered at doses optimized in this study did not significantly interfere with the integrity of the immune system or tissue homoeostasis of critical organs, even in immunocompetent mice (Supplementary Fig. 6a–c). These results support the rationale that anti-ageing agents combined with conventional chemotherapy have the potential to enhance tumour response without causing severe systemic toxicity.

### Senescent cell removal as a result of PCC1 treatment alleviates physical dysfunction

**Fig. 6:**
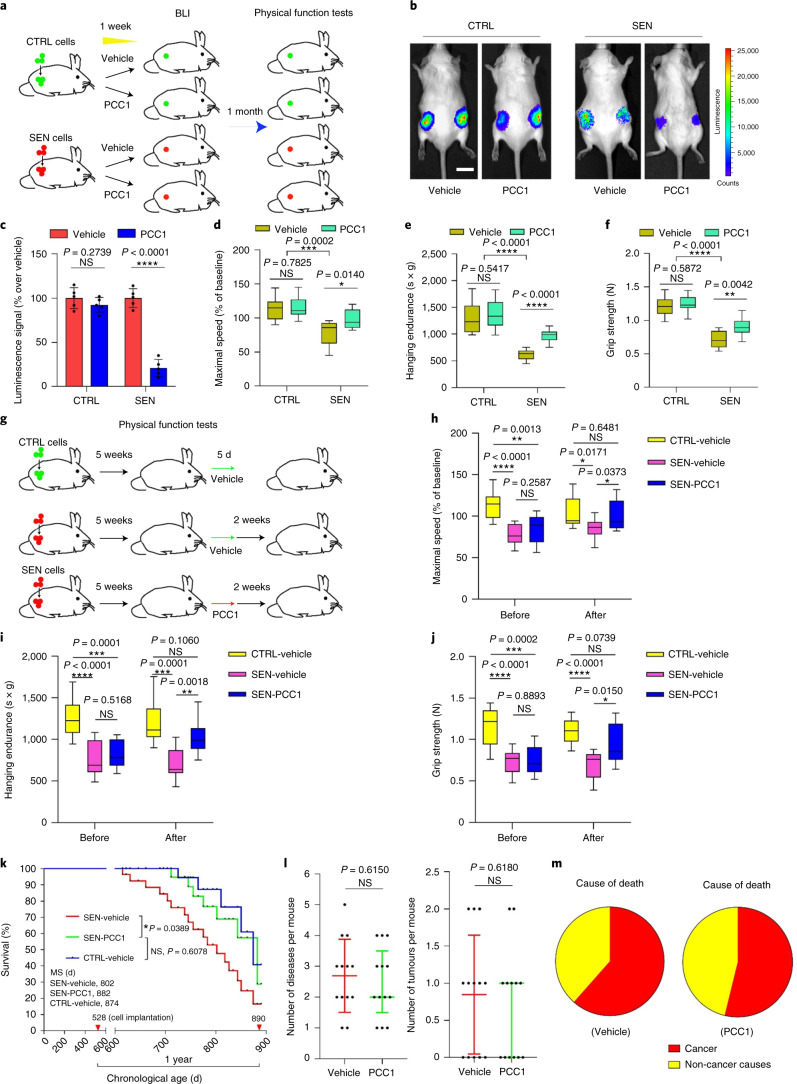
PCC1-mediated senolysis prevents physical dysfunction and alleviates pathological symptoms. **a**, Schematic of experimental procedures for cell transplantation and physical function tests in 5-month-old C57BL/6J male mice. **b**, Representative images showing in vivo luciferase activity 2 d after the last treatment of mice. Scale bars, 20 mm. **c**, Luminescence of transplanted cells as a percentage relative to the average signals in vehicle-treated animals. **d**–**f**, Measurement of maximal walking speed (relative to baseline) (**d**), hanging endurance (**e**) and grip strength (**f**) in 5-month-old C57BL/6J male mice, with tests performed 1 month after the last treatment. **g**, Schematic of the experimental design for transplantation and physical function measurements. **h**–**j**, Measurement of maximal walking speed (relative to baseline) (**h**), hanging endurance (**i**) and grip strength (**j**) in 28-week-old C57BL/6J male mice (2 weeks after the last treatment). **k**, One-year survival curves of 17-month-old animals implanted with 0.5 × 10^6^ control MEF cells and treated with vehicle (CTRL-vehicle) and mice implanted with 0.5 × 10^6^ senescent MEF cells treated either with vehicle (SEN-vehicle) or PCC1 (SEN-PCC1). Red arrowheads, cell implantation (on the 528th day of age) or the end of survival measurement (890th day of age). *P* values were calculated by two-sided log-rank (Mantel–Cox) tests. **l**, Comparative quantification of disease burden (left) and tumour burden (right) (shown as median with interquartile range) after implantation of senescent cells and treatment with vehicle or PCC1. **m**, Cause of death in animals that received implanted cells and were treated with vehicle or PCC1. For **d**–**f**,**h**–**j**, data are shown as box-and-whisker plots, in which boxes extend from the 25th to the 75th percentile with the median shown as a line in the middle, and whiskers indicate smallest and largest values. For **c**–**f**,**h**–**j**, *P* values were calculated by two-sided *t*-tests. Number of animals, *n* = 5 per group for **c**, *n* = 10 per group for **d**–**j**, *n* = 27 for **k** and *n* = 13 for **l**,**m**. NS, *P* > 0.05; *P < 0.05; **P < 0.01; ***P < 0.001; ****P < 0.0001. Source data

Even a small number of senescent cells can induce physical dysfunction in young animals^50^. We asked whether PCC1 selectively kills senescent cells in vivo and can thereby prevent physical dysfunction. To address this question, we performed parallel implantation of control and senescent mouse embryonic fibroblasts (MEFs, 0.5 × 10^6^ cells per side) constitutively expressing luciferase (LUC^+^) subcutaneously into syngeneic wild-type (WT) mice. Immediately after implantation, animals were treated with PCC1 (at 20 mg per kg via i.p. injection) or vehicle (ethanol–polyethylene glycol 400–Phosal 50 propylene glycol (PG) at 10:30:60) for 7 d (Fig. 6a). We found that luminescence signal intensities were significantly lower in mice implanted with senescent cells and treated with PCC1 than those in vehicle-treated littermates, although no difference was observed following treatment of mice transplanted with LUC^+^ control cells (Fig. 6b,c), substantiating the senolytic efficacy of PCC1 in vivo.

**Extended Data Fig. 7:**
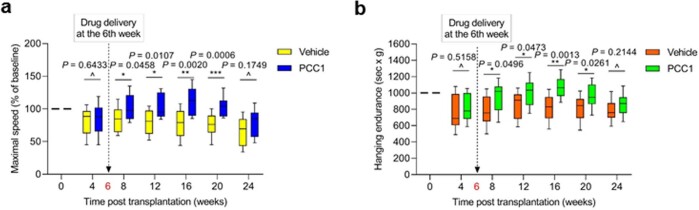
PCC1 alleviates physical dysfunction of animals transplanted with senescent cells. (**a**) Maximal walking speed (relative to the baseline) of 5-month-old C57BL/6 J males at different time points post transplantation of 1 ×10^6^ SEN stromal cells and treatment with PCC1 or vehicle (V). (**b**) Hanging endurance (measured as sec x g) of 5-month-old C57BL/6 J males at different time points as described in (**a**). Data are representative of 3 biological replicates (*n* = 10 animals *per* group). Datasets are displayed as box-and-whisker plots, where a box extends from the 25th to 75th percentile with the median shown as a line in the middle, with whiskers indicating smallest and largest values. For **a**-**b**, *P* values were calculated by two-sided *t*-test. ^, *P* > 0.05. *, *P* < 0.05. **, *P* < 0.01. ***, *P* < 0.001. Source data

We next investigated whether killing implanted senescent cells using PCC1 could attenuate pathological events, specifically physical dysfunction. Treating young animals with PCC1 after senescent cell implantation for 1 week prevented declines in maximal walking speed (RotaRod), hanging endurance (hanging test) and grip strength (grip metre), changes observed within 1 month after vehicle treatment of another group of mice carrying senescent cells, consistent with the potential of PCC1 to reduce physical dysfunction (Fig. 6d–f). PCC1 administration also prevented the physical dysfunction that occurred in animals 5 weeks following senescent cell implantation (Fig. 6g). In mice harbouring senescent cells, a single 5-d course of PCC1 treatment improved physical function compared to vehicle treatment (Fig. 6h–j). Of note, the improvement was detectable 2 weeks after PCC1 treatment and even lasted for several months (Extended Data Fig. 7a,b). At these two time points of PCC1 administration (immediately versus 5 weeks after senescent cell implantation), the beneficial effects of PCC1 seemed to be comparable. The data suggest that the timeline of PCC1 administration may be flexible, indicative of its potential clinical feasibility. As plant seed-derived procyanidins usually have elimination half-lives of <12 h^51,52^, such a sustained improvement in physical function following a single course of PCC1 treatment circumvents the need for continuous treatment with the senolytic agent, further implying that the activity of PCC1 is sufficient to avert senescent cell-induced physical dysfunction.

We next sought to evaluate the impact of senescent cells or the benefit of their elimination in middle-aged animals. For this purpose, we employed 17-month-old C57BL/6J mice, which were implanted with control or senescent MEFs. Notably, survival of animals carrying senescent cells and receiving vehicle treatment in the following year was significantly lower than that of counterparts receiving PCC1 treatment, with a 2.4-fold higher risk of death (hazard ratio, *P* = 0.0172) (Fig. 6k). However, disease burden, tumour burden at death and causes of death were not significantly different between mice treated with vehicle and those treated with PCC1 (Fig. 6l,m). These data suggest that a small number of senescent cells might affect survival through a general process, such as accelerating the progression of ageing, rather than by causing any specific pathology or a few individual conditions. Augmenting the senescent cell burden results in physical dysfunction, a tendency that is associated with mid-age mortality but can be postponed by administration of senolytics such as PCC1.

### PCC1 sustains physical function and prolongs survival of aged mice

**Fig. 7:**
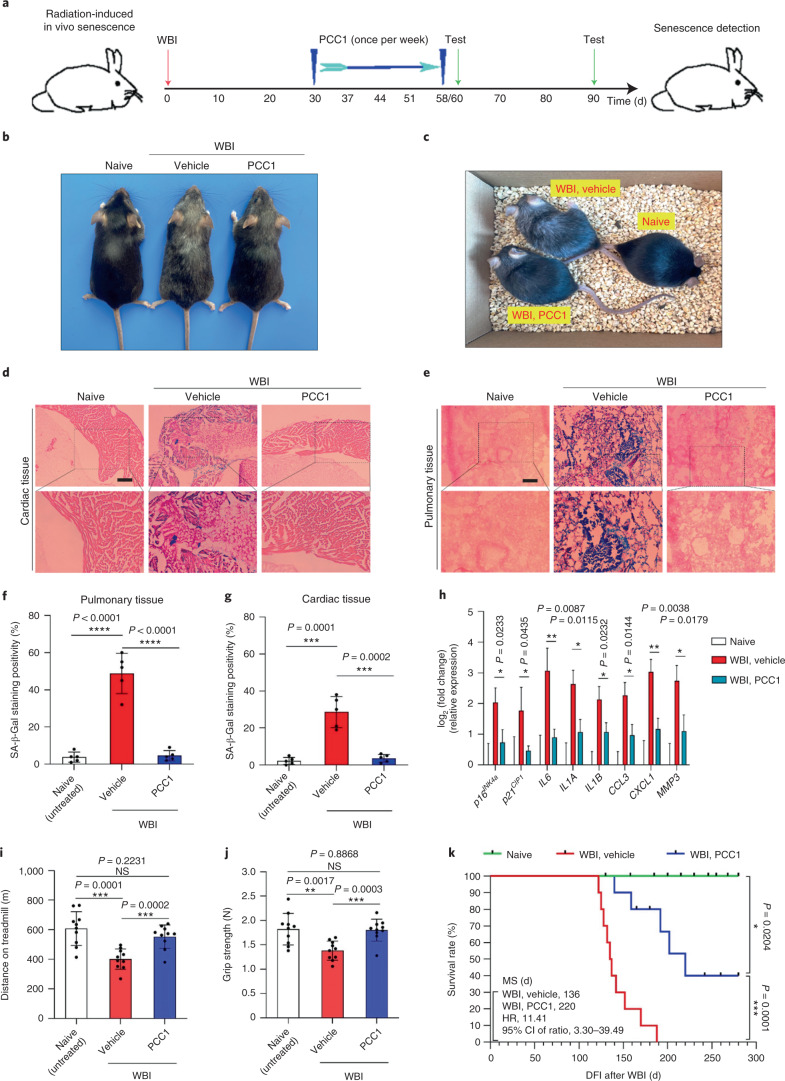
PCC1 treatment alleviates physical dysfunction of animals exposed to WBI. **a**, Schematic of the experimental procedure for mice experiencing WBI and physical function tests. **b**, Whole-body snapshot comparison of C57BL/6J male mice that were naive, exposed to WBI followed by vehicle treatment or exposed to WBI and treated with PCC1, respectively. **c**, An in-cage picture of animals described in **a** under preclinical conditions. **d**, Representative images of SA-β-Gal staining of cardiac tissue of untreated (naive) and WBI-treated mice subjected to vehicle or PCC1 treatment. Scale bar, 200 μm. **e**, Representative images of SA-β-Gal staining of pulmonary tissue of mice as described in **d**. Scale bar, 200 μm. **f**, Comparative statistics of SA-β-Gal staining of cardiac tissue of animals examined in **d**. **g**, Comparative statistics of SA-β-Gal staining of pulmonary tissue of animals examined in **e**. **h**, Quantitative measurement of SASP expression at the transcription level in tissues collected from animals treated under conditions described in **a**. **i**,**j**, Measurement of running distance on the treadmill (**i**) and grip strength (**j**) of experimental mice. For **f**–**j**, *P* values were calculated by two-sided *t*-tests. **k**, Kaplan–Meier survival analysis of C57BL/6J mice exposed to WBI and treated weekly with vehicle or PCC1, with naive mice as the untreated control. CI, confidence interval; HR, hazard ratio; DFI, disease-free interval. *P* values were calculated by two-sided log-rank (Mantel–Cox) tests. Data in bar graphs are shown as mean ± s.d. and are representative of three independent experiments. NS, *P* > 0.05; *P < 0.05; **P < 0.01; ***P < 0.001; ****P < 0.0001. Source data

Senolytics deplete senescent cells in diverse tissues and organs in various pathophysiological situations, most of which are correlated with ageing^53^. To further examine the effect of PCC1 on senescent cells in organisms and organismal ageing, we selected two independent animal models of in vivo senescence, including therapy-challenged mice and naturally ageing mice. First, we induced cellular senescence by exposing WT mice to whole-body irradiation (WBI) at a sublethal dose (5 Gy), a step followed by geroprotective treatment with PCC1 (20 mg per kg via i.p. injection) or vehicle (ethanol–polyethylene glycol 400–Phosal 50 PG at 10:30:60) (once per week) (Fig. 7a). Of note, animals that had undergone WBI manifested an abnormal body appearance, including markedly greyed hair, which, however, was largely reversed by PCC1 administration (Fig. 7b,c). SA-β-Gal-positive senescent cells were induced in vivo in these animals, as evidenced by increased staining positivity in cardiac and pulmonary tissues (Fig. 7d,e). However, when we treated with PCC1 by i.p. injection, the percentage of SA-β-Gal-positive cells in dissected tissues was significantly reduced, unlike that of vehicle-treated mice at the post-WBI stage (Fig. 7f,g). PCC1 treatment also decreased the expression of senescence markers and a subset of key SASP factors compared with vehicle treatment (Fig. 7h). In sum, the data suggest that PCC1 can effectively deplete SA-β-Gal-positive cells, control SASP expression and minimize senescent cell burden under in vivo conditions in mice.

We then assessed the impact of preclinical treatments on the physical parameters of mice. As expected, WBI significantly compromised exercise capacity and muscle strength as measured by treadmill and grip strength assays in the vehicle group (Fig. 7i,j). By contrast, PCC1 administration provided substantial benefit, restoring these capacities. More importantly, PCC1 treatment increased the survival rate (Fig. 7k). Our results indicate that PCC1-induced elimination of SA-β-Gal-positive senescent cells could be an effective strategy to alleviate senescence-related physical regression and reduce mortality in settings of premature ageing triggered by environmental stressors such as cytotoxic therapy.

**Fig. 8:**
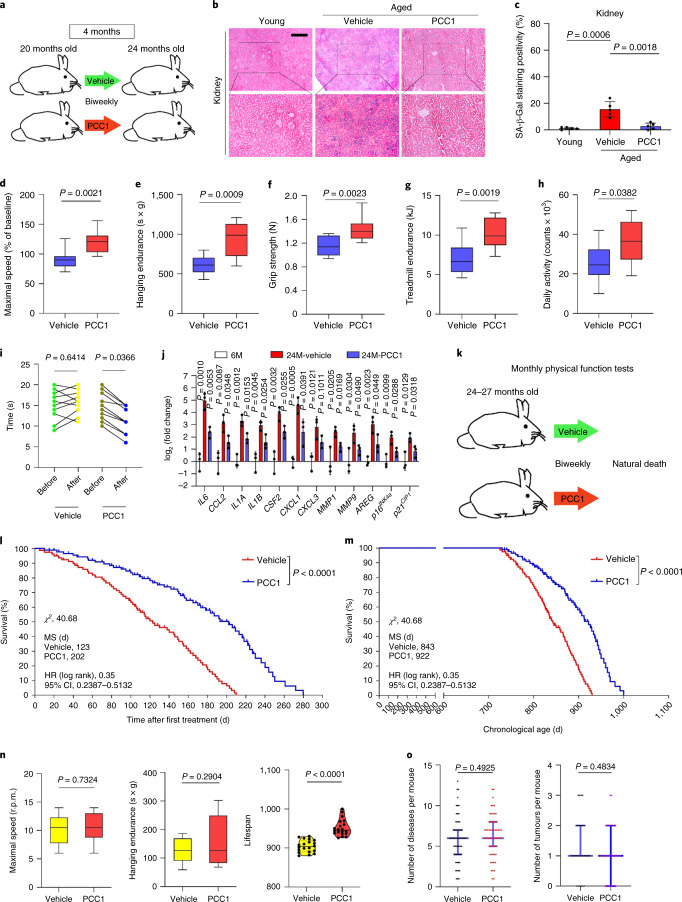
Intermittent PCC1 administration extends both healthspan and lifespan of aged mice. **a**, Schematic design for physical examination of 20-month-old C57BL/6J male mice treated with PCC1 once every 2 weeks (biweekly) for 4 months. **b**, Representative images of SA-β-Gal staining of kidneys from young and aged mice treated with vehicle or PCC1. Scale bar, 200 μm. **c**, Quantification of SA-β-Gal staining as described in **b**. Data represent mean ± s.d. **d**–**h**, Quantification of maximal walking speed (relative to baseline) (**d**), hanging endurance (**e**), grip strength (**f**), treadmill endurance (**g**) and daily activity (**h**) of 20-month-old C57BL/6J male mice after the 4-month treatment. **i**, Quantification of the time needed to cross the balance beam. Data points before and after treatment of each animal are connected to allow direct comparison of treatment effects. **j**, Quantitative transcript profiling of SASP expression in lung tissues collected from 6-month-old untreated (6M), 24-month-old vehicle-treated (24M-vehicle) and 24-month-old PCC1-treated mice (24M-PCC1). Data are shown as mean ± s.d. and were derived from three biological replicates (*n* = 3 independent assays). **k**, Schematic design for lifespan analyses of mice (both sexes) at 24–27 months of age. **l**,**m**, Post-treatment survival (**l**) and whole-life survival (**m**) curves of C57BL/6J animals treated biweekly with PCC1 (*n* = 91; 48 males, 43 females) or vehicle (*n* = 80; 42 males, 38 females) starting at 24–27 months of age. **n**, Maximal walking speed and hanging endurance averaged over the last 2 months of life (*n* = 10 mice per group) and lifespan for the longest-living mice (top 20) in both groups. **o**, Disease burden and tumour burden at death. For both sexes, *n* = 60 mice per arm. For males, *n* = 31 for PCC1 and *n* = 33 for vehicle. For females, *n* = 29 for PCC1 and *n* = 27 for vehicle. For **c**–**h**,**j**, *n* = 3 biologically independent assays. Data are displayed as box-and-whisker plots, in which a box extends from the 25th to the 75th percentile with the median shown as a line in the middle, and whiskers indicate smallest and largest values (**d**–**h**,**n**) or as mean ± s.d. (**o**). Unpaired two-tailed *t*-tests (**c**–**j**,**n**,**o**) and Cox proportional-hazard regression models (**l**,**m**) were used to determine statistical significance. Source data

**Extended Data Fig. 8:**
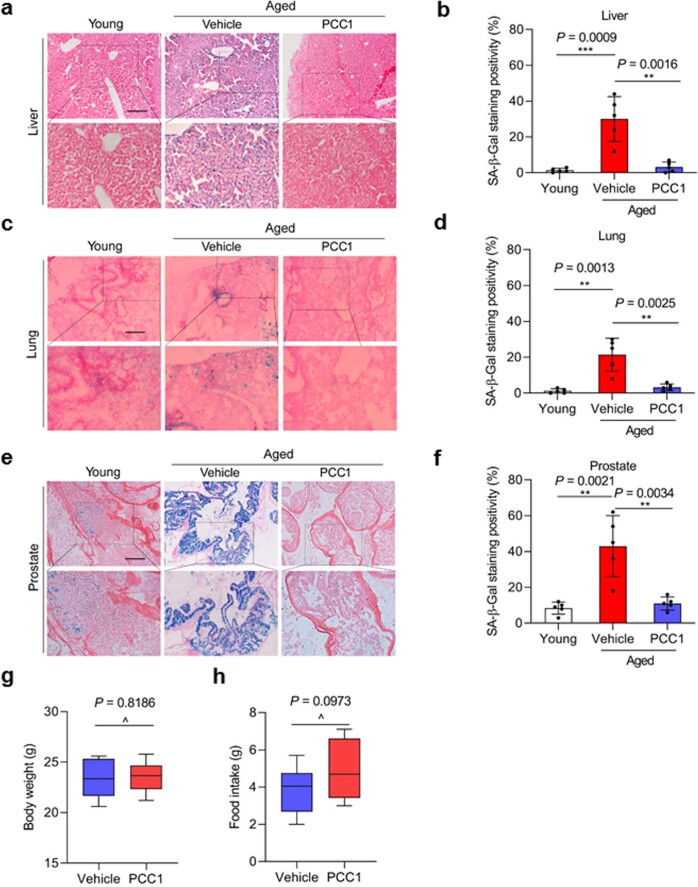
Intermittent administration of PCC1 alleviates physical dysfunction of aged mice. (**a**) Representative images of SA-β-Gal staining of liver tissues from young (6 months of age) and aged males treated with vehicle or PCC1. Scale bar, 200 μm. (**b**) Comparative statistics of SA-β-Gal staining positivity of samples assayed in (**a**). (**c**) Representative images of SA-β-Gal staining of lung tissues from young and aged mice treated with vehicle or PCC1. Scale bar, 200 μm. (**d**) Comparative statistics of SA-β-Gal staining positivity of samples examined in (**c**). (**e**) Representative images of SA-β-Gal staining of prostate tissues from young and aged mice treated with vehicle or PCC1. Scale bar, 200 μm. (**f**) Comparative statistics of SA-β-Gal staining positivity of samples examined in (**e**). (**g**) Quantification of body weight of animals as described in (**a**). (**h**) Measurement of food intake of animals as described in (**a**). Data in **g** and **h** are representative of 3 biological replicates (*n* = 10 animals *per* group). Datasets are displayed as box-and-whisker plots, where a box extends from the 25th to 75th percentile with the median shown as a line in the middle, with whiskers indicating smallest and largest values. Data in bar graphs (**b**, **d** and **f**) are shown as mean ± SD (*n* = 5 animals *per* group) and representative of 3 independent experiments. For **b**, **d**, **f**, **g** and **h**, *P* values were calculated by two-sided *t*-test. ^, *P* > 0.05. *, *P* < 0.05. **, *P* < 0.01. ***, *P* < 0.001. Source data

We next sought to define the impact of senescent cells on physical function in naturally ageing animals. For this purpose, we treated normal 20-month-old WT mice with vehicle (ethanol–polyethylene glycol 400–Phosal 50 PG at 10:30:60) or PCC1 (20 mg per kg via i.p. injection) (once every 2 weeks) for 4 months (Fig. 8a). Histological evaluation revealed a significantly elevated percentage of SA-β-Gal-positive senescent cells in the kidney, liver, lung and prostate of aged animals, which was reversed by PCC1 treatment (Fig. 8b,c and Extended Data Fig. 8a–f). Results from physical testing showed that PCC1 alleviated physical dysfunction by enhancing maximal walking speed, hanging endurance, grip strength, treadmill endurance, daily activity and beam balance performance of animals administered PCC1 compared to those treated with vehicle (Fig. 8d–i), Body weight and food intake remained largely unchanged in PCC1-treated mice (Extended Data Fig. 8g,h). Notably, expression of the SASP was significantly reduced in tissues such as the lungs of aged mice treated with PCC1 compared to that in the vehicle-treated group (Fig. 8j), a pattern consistent with lesser secretion of SASP factors by human stromal tissues treated with PCC1 (Fig. 5f).

To establish the potential of senescent cell elimination to extend the remaining lifespan of WT mice, we performed PCC1 treatment beginning at a very old age (Fig. 8k). Mice receiving PCC1 administration (once every 2 weeks or biweekly) starting at 24–27 months of age (roughly equivalent to an age of 75–90 years in humans) had a 64.2% longer median post-treatment lifespan (or 9.4% longer overall lifespan) and lower mortality hazard (65.0%, *P* < 0.0001) than the vehicle-treated group (Fig. 8l,m). These data indicate that PCC1 can significantly decrease the risk of age-associated mortality in old mice.

**Extended Data Fig. 9:**
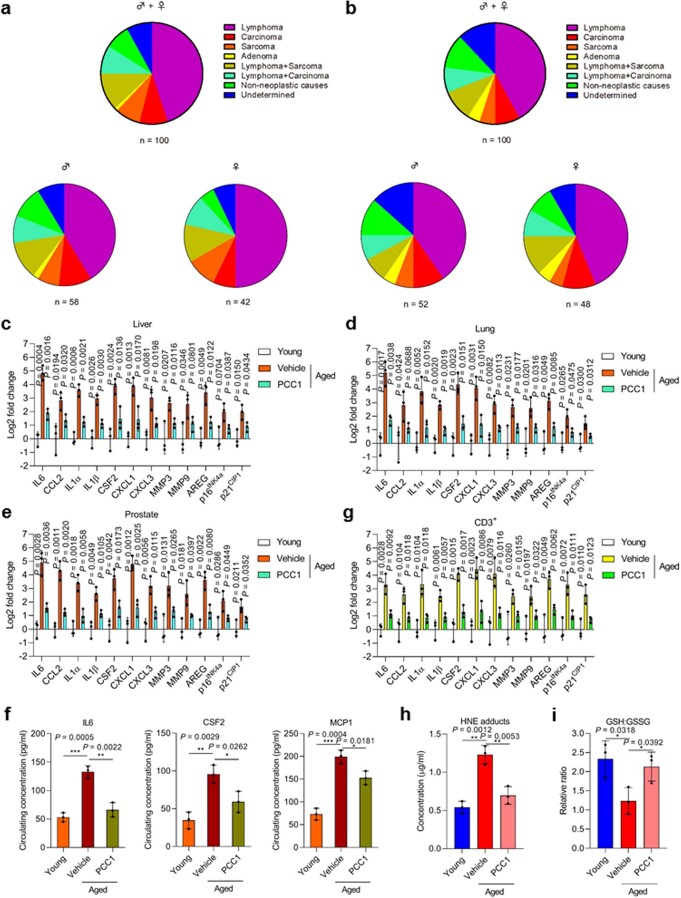
Late life intervention with PCC1 does not alter the cause of death, but restrains the SASP and reduces oxidative stress. (**a**-**b**) Pie charts depicting the ultimate causes of death of C57BL/6 J mice (males and females) that had undergone vehicle (**a**) or PCC1 (**b**) biweekly treatment starting from 24-27 months of age. Note there was no significant difference between vehicle- and PCC1-treated groups upon analysis using either Chi-square or Fisher’s exact tests. (**c**-**e**) qRT-PCR profiling of the SASP and senescence marker expression in tissues of solid organs, including liver (**c**), lung (**d**) and prostate (**e**) collected from young (6-month-old, untreated), aged (24-27-month-old) vehicle-treated and aged (24-27-month-old) PCC1-treated animals, respectively. (**f**) Measurement of circulating levels of hallmark SASP factors IL6, CSF2 and MCP-1 in mouse blood by ELISA assays. (**g**) Quantification of SASP and senescence marker expression in CD3^+^ peripheral T cells of experimental mice described in **c**-**f**. (**h**) Examination of 4-hydroxynonenal (HNE) adducts, a marker of lipid peroxidation and oxidative stress by ELISA measurement with tissue lysates of the liver. (**i**) Determination of the ratio of reduced (GSH) to oxidized (GSSG) glutathione measured as an index oxidative stress. Data are shown as mean ± SD and derive from 3 biological replicates (*n* = 3 independent assays for **c**-**i**). *P* values were calculated by two-sided *t*-test. ^, *P* > 0.05. *, *P* < 0.05. **, *P* < 0.01. ***, *P* < 0.001. Source data

We next queried whether the reduced death rate in aged animals came at a cost of increased late-life morbidity. We measured physical function in experimental mice treated with PCC1 or vehicle monthly until death. Despite the longer remaining lifespan in PCC1-treated mice, physical function in the last 2 months of life was not significantly lower than that in vehicle-treated mice (Fig. 8n). Upon autopsy, the incidence of several age-related pathologies, tumour burden and cause of death were not significantly different between PCC1-treated and vehicle-treated mice (Fig. 8o and Extended Data Fig. 9a,b). However, expression of the SASP was reduced in solid organs, which was largely compatible with the decline of circulating levels of interleukin (IL)-6, colony-stimulating factor (CSF)2 and monocyte chemoattractant protein (MCP)1, representative SASP markers in peripheral blood (Extended Data Fig. 9c–f). We also observed decreased expression of the SASP in CD3^+^ T cells in peripheral blood (Extended Data Fig. 9g), a cell lineage that exhibits a robust increase in p16^INK4a^ expression during human ageing^54^. Furthermore, PCC1 treatment reduced oxidative stress in liver tissues, as evidenced by a decrease in adducts of the lipid peroxidation product 4-hydroxynonenal (HNE) and an increase in the ratio of reduced to oxidized glutathione (Extended Data Fig. 9h,i), consistent with the general properties of flavonoids, which exert antioxidant activity by counteracting free radicals and engaging the antioxidant defence system^55,56^.

In sum, the senolytic agent PCC1, a phytochemical component derived from GSE (or alternatively, at lower abundance, from natural products such as extracts of cinnamon, cacao, apple peels and pine bark), can reduce the burden of senescent and possibly other cells developing a pro-inflammatory phenotype and inherently dependent on pro-survival senescence-associated anti-apoptotic pathways and increase post-treatment lifespan without causing elevated morbidity in mice. We hereby present proof-of-principle evidence that, even when administered in late life, such a therapeutic modality holds prominent potential to remarkably delay age-related dysfunction, reduce age-related diseases and enhance health conditions, thus providing a new avenue to improve healthspan and lifespan in future geriatric medicine.

## Discussion

Ageing is an essentially inevitable process that progressively causes functional decline in nearly all organisms. Cellular senescence, a state of permanent growth arrest, has recently emerged as both a hallmark and a driver of ageing^3,57^. Senescent cells accumulate in aged tissues over time and contribute to an increasing list of pathologies^58^. Clearance of senescent cells from progeroid or naturally aged mice extends healthspan, increases lifespan and restrains age-related disorders including but not limited to atherosclerosis, osteoarthritis and neurodegenerative diseases^59–62^. Recent advances in age-related studies prompted a search for drugs that can selectively target senescent cells, particularly a new class of geroprotective agents termed senolytics or, less aggressively, senomorphics. To date, a handful of senolytics have been reported, including dasatinib and quercetin, fisetin, piperlongumine, heat-shock protein (HSP)90 inhibitors and BCL-2 family inhibitors such as ABT-263 (navitoclax) and ABT-737 (refs. ^11–13,18,19,21,22^). Among them, BCL-2 inhibitors are the most widely used senolytics, although originally developed as therapies for lymphoma. ABT-737 targets BCL-2, BCL-xL and BCL-w but with low solubility and oral bioavailability. More effective for in vivo use, ABT-263 mainly inhibits BCL-2 and BCL-xL, whereas it frequently causes thrombocytopaenia. Given the marked side effects of some senolytic compounds, there is a need to identify new compounds with senolytic activity but reduced cytotoxicity. In this study, we screened a PDMA-based drug library composed mainly of natural products with an aim to identify new agent(s) that can widely target senescent cells with optimal in vivo efficacy and safety. As a result, we identified PCC1, a phytochemical agent derived from natural sources, as a broad-spectrum senolytic compound. As a special advantage, PCC1 can alternatively act as a senomorphic agent to minimize SASP expression when used at low concentrations. Such an advantageous feature of PCC1 indeed largely resembles that of GSE, which can generate both senomorphic and senolytic effects.

Genetic and pharmacological strategies demonstrated an array of benefits of eliminating senescent cells to delay ageing and control diseases. Cellular senescence can be triggered by a variety of stimuli ranging from oncogenic activation, genotoxic stress, to inflammatory response and replicative exhaustion. Several compounds are identified as broad-spectrum senolytics, while others are selective against only a certain type of senescent cell. Differences in specificity imply individual choices of senolytics, which mainly depend on their intended clinical use. A recent study revealed ouabain, a natural compound belonging to the cardiac glycoside family, as a senolytic agent that can be used for both senescent cell elimination and cancer therapy, the latter implemented through a dual mechanism of action^63^. In this work, we discovered PCC1 as another new, natural and potent senolytic, which selectively and specifically induces apoptosis of senescent cells but with limited cytotoxicity to proliferating cells^64^. Of note, at lower concentrations, PCC1 inhibits SASP expression, a property shared by some plant-derived flavonoids such as apigenin and kaempferol, which can act as senomorphics to limit the impact of senescent cells on age-related conditions^65,66^. Although few studies have disclosed such a dual mechanism of natural agents in targeting senescent cells, the recently synthesized quercetin surface functionalized Fe_3_O_4_ nanoparticles exhibited both senolytic and senomorphic potential in human fibroblasts by enhancing AMP-activated protein kinase (AMPK) activity^67^.

The mechanism by which PCC1 achieves senolytic effects appears complex and requires further study. Our data suggest that PCC1 impairs the functional integrity of mitochondria, compromising Δψm, leading to increased production of free radicals such as ROS and causing cytochrome c release in senescent cells but not in proliferating cells. A possible reason for this specificity is that senescent cells tend to develop a depolarized plasma membrane and have increased concentrations of H^+^ (ref. ^64^), a feature that might make them more susceptible to the action of PCC1. Of note, these alterations are accompanied by upregulated expression of pro-apoptotic factors, specifically NOXA and PUMA, events that also critically promote senescent cell apoptosis. Within the family of procyanidins, members of which are known to derive from the polymerisation of flavan-3-ol molecules and exist as oligomers or polymers^28^, PCC1 seems to be functionally unique. Our experimental data imply a noticeable difference between PCC1 (a trimer) and other procyanidins (most of which are indeed monomers or dimers, such as PCB2). Since we did not comprehensively assay procyanidin family members, whether the number of monomers in the molecule determines its anti-senescence potential remains an open but intriguing question, and the underlying mechanisms deserve continued studies in the future.

Cellular senescence per se is a highly heterogeneous process that depends on different cell origins and environmental stimuli^68^. One of the key features of PCC1 is its ability to efficiently clear senescent cells in a wide spectrum of cell types and stressors, including replication, oncogenes, irradiation and chemotherapy. In this study, we compared PCC1 with other reported senolytics for effects on human stromal cells, fibroblasts, HUVECs and MSCs, major cell types in the tissue microenvironment. As reported, ABT-263 eliminates senescent human embryonic fibroblasts (HEFs) and HUVECs but has little effect on human pre-adipocytes^12,18^. The combined use of dasatinib and quercetin can deplete all three types of senescent cells in a dose-dependent manner but is toxic to proliferating cells^11,69,70^. Fisetin, another natural flavonoid reported as a senolytic agent, displays modest effects on senescent HEFs and pre-adipocytes only at high concentrations^20,21^. By contrast, PCC1 has the potential to overcome these limitations, including cell type dependency, high toxicity in nonsenescent cells and low efficiency against senescent cells. Although, when used alone, quercetin (another flavonoid in GSE) per se displayed cytotoxicity against senescent stromal cells, its efficacy is generally lower than that of PCC1 (compare Fig. 2a,c and Supplementary Figs. 3n and 4n). Together, PCC1 has a superior senolytic activity with high specificity and efficiency for a wider range of cell types than many reported senolytics such as ABT-263, dasatinib, quercetin and fisetin and can target senescent cells generated by several major types of senescence inducers.

We found that PCC1 exerts apoptosis-inducing effect on senescent cells under in vivo conditions. PCC1 eliminated therapy-induced senescent cells effectively and reduced senescence markers in solid organs, highlighting its effectiveness in vivo. In this study, we also treated naturally aged mice with PCC1 and tested its effects on senescent cells, chronic inflammation and physical function. First, PCC1 treatment depleted senescent cells in multiple tissues and decreased SASP-associated signatures as shown by GSEA analysis. Second, PCC1 could suppress expression of SASP-associated genes in aged livers and kidneys and reduce chronic low-grade inflammation in the blood. Third, PCC1 alleviated impaired motor function, balance, exhausted exercise, muscle strength and spontaneous exploration in aged mice. Most importantly, performance on RotaRod and beam balance testing in the PCC1-treated group was improved compared with that in the initial pretreatment condition. Collectively, the phytochemical compound PCC1 selectively targets senescent cells in the tissue microenvironment and generates remarkable biological effects in naturally aged mice.

Similar to chemically synthesized counterparts, naturally derived procyanidins manifest anti-inflammatory, anti-arthritic, anti-allergic and anticancer activities, scavenge oxygen free radicals and suppress radiation-induced peroxidation activity^36,71^. As an epicatechin trimer isolated from plant material, most prominently from grape seeds, PCC1 was shown to provide health benefits in chronic pathological conditions^72^. However, thorough evaluation of the toxicological effects of PCC1 in vivo is crucial for a potential clinical application. Our data showed that high-concentration (20 mg per kg) and high-frequency PCC1 (biweekly) treatment had no apparent systemic toxicities. In summary, our study demonstrates the superiority and relative safety of a geroprotective strategy that selectively targets senescent cells in aged or treatment-damaged tissues across a broad spectrum of cell types. However, it is possible that PCC1 concentrations in vivo vary between organs and depend on the administered dose, pharmacodynamics and pharmacokinetics and that local concentrations are not high enough to achieve a senolytic effect in some tissue types. In this case, it seems likely that a combination of both senolytic and senomorphic effects underlies the outcomes that we observed in vivo.

Altogether, our study opens a new avenue for extending healthspan and prolonging lifespan and treating age-related pathologies with a senotherapeutic agent (with both senomorphic and senolytic potential), which is derived from natural sources and possesses pronounced efficacy. The potential anti-ageing effects of PCC1 demonstrated in our preclinical assays provide good support for further translational and clinical development of PCC1, with the overall aim of achieving a longer and healthier life.

## Methods

### Preclinical animal studies

All procedures with experimental animals were approved by the Institutional Animal Care and Use Committee (IACUC) at Shanghai Institute of Nutrition and Health, Chinese Academy of Sciences, with all animals handled in accordance with the guidelines for animal experiments defined by the institutional IACUC.

### Cell lines, in vitro culture and lentiviruses

The human primary prostate stromal cell line PSC27 was kindly provided by P. Nelson (Fred Hutchinson Cancer Research Center) and cultured in PSC complete medium (80% MCDB131 (Thermo Fisher Scientific) supplemented with 10% FCS, nonessential amino acids, insulin, dexamethasone, transferrin, selenium and 20% AmnioMAX (Thermo Fisher Scientific)) as described previously^15^. The human foetal lung fibroblast cell line WI38 and the HUVEC line were purchased from ATCC (CCL-75 and PCS-100-010, respectively) and maintained in DMEM and F-12K medium, respectively, as recommended by the provider. The MSC line was derived from human umbilical vein tissues and cultured in MSC complete medium with 10 μg ml^−1^ recombinant human insulin as reported^73^. All cell lines tested negative for microbial contamination and were routinely authenticated with STR assays.

Lentiviral particles were produced using Lipofectamine 2000 and a packaging kit (Thermal Scientific) based on the manufacturer’s instructions. PSC27 cells infected with viruses with the puromycin resistance gene were selected using puromycin (1 μg ml^−1^) for 3 d.

### Cell treatment and analyses

Stromal cells were grown until 60–80% confluent (control) and treated with bleomycin (50 μg ml^−1^, MedChemExpress) for 12 h. After treatment, cells were rinsed twice with PBS and maintained for 7–10 d in medium. Alternatively, cells were passaged consecutively to induce replicative exhaustion as RS or lentivirally infected with a construct encoding full length HRAS^G12V^ and selected with puromycin (1 μg ml^−1^) for 3 d and maintained for 7 d until senescence (OIS).

DNA damage was evaluated by immunostaining for γH2AX or p53-BP1 foci following a four-category counting strategy as previously reported^15^. Random fields were chosen to quantify DDR foci using CellProfiler (http://www.cellprofiler.org). For experiments with specific needs, HS-1793 (TargetMol), CCCP (TargetMol) and ruxotemitide (LTX325) (Selleckchem) were used at concentrations of 10 μM, 10 μM and 50 μg ml^−1^, respectively. For clonogenic assays, cells were seeded at 1 × 10^3^ cells per dish in 10-mm dishes for 24 h before being treated with chemicals. Cells were fixed in 2% paraformaldehyde 7–10 d after treatment, gently washed with PBS and stained instantly with 10% crystal violet in 50% methanol. Excess dye was removed with PBS, and plates were photographed. Colony formation was quantified by counting single colonies per dish.

### Cell viability assays

Senescent or control cells were seeded into wells of a 96-well plate before being treated with vehicle (0.1% DMSO or PBS) or natural product library agents (listed in Supplementary Table 5) with 5,000 cells per well at three concentrations (1, 5 and 10 μg ml^−1^ for agents of unknown molecular weight; or 1, 5 and 10 μM for agents of known molecular weight) for three consecutive days. Cultures were digested with 0.25% trypsin and 1 mM EDTA and collected in PBS containing 2% FBS, with survival measured by a cell-based MTS assay (Promega). The top candidate agents were further screened in a 30-d treatment at additional concentrations. MSCs (P5–P10) were seeded into six-well plates at a density of 30,000 cells per well. Culture medium with the different candidate agents was changed every other day. PD potential was quantified by determining the relative proliferative capacities of MSCs treated with agents of senotherapeutic capacity, and cells with potential to recover after withdrawal of bleomycin from the medium were allowed to expand for PD measurement.

Apoptosis was assessed using an annexin V–FITC cell apoptosis assay kit (Beyotime). Briefly, cells were sequentially incubated with annexin V–FITC and PI before being sorted using a Beckman flow cytometer (Beckman, CytoFLEX LX). Viable cells (PI^−^ cells) were analysed at a constant flow rate and calculated as a percentage of control cells treated with vehicle using the following formula: percentage of control = (*N*_drug_*N*_c_^−1^) × 100, where *N*_drug_ and *N*_c_ represent the absolute number of PI^−^ viable drug-treated or vehicle-treated cells, respectively. Dose–response curves were constructed for each tested agent, and half-maximal inhibitory concentrations were calculated using probit analysis^74^.

### Radiation of animals

Animals were housed in a specific pathogen-free animal facility at an ambient temperature of 22–25 °C with 50% humidity, under a 12-h light–dark cycle, with free access to food and were fed ad libitum. For induction of in vivo senescence, C57BL/6J mice (males, Nanjing Model Animal Center) at 8–12 weeks of age were exposed to a sublethal dose (5 Gy) of WBI. Eight weeks later, animals were injected with 20 mg per kg PCC1 i.p. or vehicle once per week for four consecutive weeks. Mice were killed 24 h after the last injection. Mouse tissues were collected for RNA extraction, paraffin embedded for immunohistology or frozen in OCT solution for cryosectioning and SA-β-Gal staining. Mice used for all experiments were randomly assigned to control or treatment groups. Both sexes were used throughout the study.

The experimental procedure for animal radiation was approved by the IACUC at Shanghai Institute of Nutrition and Health, Chinese Academy of Sciences, with all animals handled in accordance with the guidelines for animal experiments defined by the institutional IACUC.

### Chemotherapeutic regimens

NOD–SCID mice (males, Nanjing Model Animal Center) at 6–8 weeks of age were housed and maintained in accordance with the animal guidelines of Shanghai Institutes for Biological Sciences. For establishment of mouse tumour xenografts, human prostate stromal cells (PSC27 cells) and cancer epithelial cells (PC3 or PC3-Luc) were mixed at a ratio of 1:4, with each in vitro recombinant comprising 1.25 × 10^6^ total cells before subcutaneous implantation as described previously^14,47^. Two weeks later, mice were randomized into groups and subjected to preclinical treatments. Animals were treated with MIT (0.2 mg per kg) alone, PCC1 (20 mg per kg) alone or MIT with PCC1. MIT was delivered via i.p. injection biweekly starting from the beginning of the 3rd week, with a total of three 2-week cycles throughout the whole regimen as reported previously^16,17^. PCC1 was delivered via i.p. injection 2 weeks after the first dose of MIT and then given biweekly, with a total number of two doses throughout the regimen.

Mice were killed at the end of the 8th week after tumour xenografting. Primary tumours were measured once biweekly starting from the beginning of the 4th week and after animal dissection, and the approximate ellipsoid tumour volume (*V*) was measured and calculated from the tumour length (*l*) and width (*w*) using the following formula: *V* = (π × 6^−1^) × ((*l* + *w*) × 2^−1^)^3^. Excised tumours were either freshly snap-frozen or fixed in 10% buffered formalin and subsequently processed as formalin-fixed and paraffin-embedded sections for IHC staining. Animals were weighed weekly and checked for tumour growth semi-weekly. Bulky disease was considered present when the tumour burden was prominent at the hind flank (tumour volume ≥ 2,000 mm^3^). When at least five of the ten mice in a treatment group had developed bulky disease, the median survival duration for that group was considered to be reached. Throughout the regimen, tumours were not allowed to grow further if the maximal threshold was surpassed (any dimension ≥15 mm), and mice were immediately killed according to the animal care and use protocol.

Tumour growth and metastasis in mice were alternatively evaluated using bioluminescence emitted by PC3-Luc cells, which stably express firefly luciferase. A Xenogen IVIS Imager (Caliper Life Sciences) was used to document bioluminescence across the visible spectrum in isoflurane-anaesthetized animals, with the substrate d-luciferin (150 mg per kg, BioVision) injected subcutaneously and freshly each time for imaging-based tumour surveillance.

The chemotherapeutic procedure was approved by the IACUC at Shanghai Institute of Nutrition and Health, Chinese Academy of Sciences, with all animals treated in accordance with the guidelines for animal experiments defined by the institutional IACUC.

### Senescent cell clearance and lifespan studies

For age-related studies, WT C57BL/6J male mice were maintained in a specific pathogen-free facility at 22–25 °C under a 12-h–12-h light–dark cycle (lights on from 08:00 to 20:00) with free access to food (standard mouse diet, LabDiet 5053) and water provided ad libitum. The experimental procedure was approved by the IACUC at Shanghai Institute of Nutrition and Health, Chinese Academy of Sciences, with all animals treated in accordance with the guidelines for animal experiments defined by the IACUC.

Mice were sorted by body weight from low to high, and pairs of mice were selected according to similar body weights. Either control cell or senescent cell transplant treatments were assigned to every other mouse using a random number generator, with the intervening mice assigned to the other treatment to allow mice transplanted with control and senescent cells to be matched by weight. After acclimation in the animal facility at Shanghai Institute of Nutrition and Health, mice aged 5 months were subcutaneously transplanted with MEFs (0.5 × 10^6^ cells per side) and treated immediately with vehicle or PCC1 (prepared in ethanol–polyethylene glycol 400–Phosal 50 PG at 10:30:60, 20 mg per kg) for 1 week (i.p. injection once every 3 d) before bioluminescence imaging assays. Physical function tests were performed 1 month after bioluminescence imaging at the age of approximately 25 weeks. The first death occurred approximately 3 months after the last physical function test. For treatment-delayed mice that received cell transplantation, a first wave of physical function tests was conducted 5 weeks after cell transplantation. Animals were then subjected to treatment with vehicle for 5 d (for those with control cells as the first group) or vehicle or PCC1 for 5 d (for those with senescent cells, as the second and third groups, respectively) (i.p. injections were performed consecutively for each condition once every 5 d). Mice were maintained for 2 weeks, after which the second wave of physical function assays was conducted. For mid-age studies, 17-month-old C57BL/6J mice were implanted with control or senescent MEF cells and underwent treatment with vehicle or PCC1. Administration was performed once biweekly, and animals were measured for survival within a 1-year time frame after cell implantation.

For senolytic studies of natural ageing, 20-month-old WT C57BL/6J mice (males) without transplantation were used and were sorted according to their body weight and randomly assigned to vehicle or PCC1 treatment. Animals were treated once every 2 weeks in an intermittent manner for 4 months before physical tests were performed at 24 months of age. For senolytic trials pertaining to lifespan extension at advanced age, we used animals at a very old age. Starting at 24–27 months of age (equivalent to a human age of 75–90 years), mice (both sexes) were treated once every 2 weeks (biweekly) with vehicle or PCC1 by oral gavage (20 mg per kg) for three consecutive days. Some mice were moved from their original cages during the study to minimize single cage-housing stress. In each case, RotaRod (TSE Systems) and hanging tests were chosen for monthly measurement of maximal speed and hanging endurance, respectively, as these tests are considered sensitive and noninvasive. Mice were euthanized and scored as having died if they exhibited more than one of the following signs: (1) unable to drink or eat, (2) reluctant to move even with stimulus, (3) rapid weight loss, (4) severe balance disorder or (5) bleeding or ulcerated tumour. No mouse was lost due to fighting, accidental death or dermatitis. The Cox proportional-hazard model was used for survival analyses.

### Postmortem pathological examination

Animal cages were checked every day, and dead mice were removed from cages. Within 24 h, corpses were opened (abdominal cavity, thoracic cavity and skull) and preserved in 4% paraformaldehyde individually for at least 7 d, and decomposed or disrupted bodies were excluded. Preserved bodies were given to pathologists for blinded examination, following an assessment routine. Briefly, tumour burden (sum of different types of tumours in each mouse), disease burden (sum of different histopathological changes in major organs in each mouse), severity of each lesion and inflammation (lymphocytic infiltrate) were evaluated.

### Bioluminescence imaging

Experimental mice were injected i.p. with 3 mg d-luciferin (potassium salt, BioVision) in 200 µl PBS. Mice were anaesthetized by inhalation of isoflurane, and bioluminescence images were acquired using a Xenogen IVIS 200 System (Caliper Life Sciences) according to the manufacturer’s instructions.

### Physical function assessments

All physical tests were performed at least 5 d after the last dose of senolytic treatment. Maximal walking speed was measured using an accelerating RotaRod system (TSE Systems). Mice were trained on the RotaRod for 3 d at speeds of 4, 6 and 8 r.p.m. for 200 s on days 1, 2 and 3. On the test day, mice were placed onto the RotaRod, which was started at 4 r.p.m. The rotating speed was accelerated from 4 to 40 r.p.m. over a 5-min interval. The speed was recorded when the mouse dropped off the RotaRod, with results averaged from three or four trials and normalized to the baseline speed. Training was not repeated for mice that had been trained within the preceding 2 months. Forelimb grip strength (N) was determined using a Grip Strength Meter (Columbus Instruments), with results averaged over ten trials. To measure grip strength, the mouse is swung gently by the tail so that its forelimbs contact the bar. The mouse instinctively grips the bar and is pulled horizontally backwards, exerting tension. When the tension becomes too great, the mouse releases the bar. For the hanging test, mice were placed onto a metal wire (2 mm thick) that was 35 cm above a padded surface, while animals were allowed to grab the wire with their forelimbs only. Hanging time was normalized to body weight as hanging duration (s) × body weight (g), with results averaged from two or three trials for each mouse. A Comprehensive Laboratory Animal Monitoring System (CLAMS, Columbus Instruments) was used to monitor daily activity and food intake over a 24-h period (12 h of light and 12 h of dark). The CLAMS system was equipped with an Oxymax Open Circuit Calorimeter System (Columbus Instruments). For treadmill performance, mice were acclimated to a motorized treadmill at an incline of 5° (Columbus Instruments) over 3 d for 5 min each day, starting at a speed of 5 m min^−1^ for 2 min and progressing to 7 m min^−1^ for 2 min and then 9 m min^−1^ for 1 min. On the test day, mice ran on the treadmill at an initial speed of 5 m min^−1^ for 2 min, and then the speed was increased by 2 m min^−1^ every 2 min until the mice were exhausted. The speed at which the mouse dropped from the RotaRod was recorded, and results were averaged from three trials. Exhaustion was defined as the inability to return onto the treadmill despite a mild electrical shock stimulus and mechanical prodding. Distance was recorded, and total work (kJ) was calculated using the following formula: mass (kg) × *g* (9.8 m s^−2^) × distance (m) × sin (5°).

### Comprehensive laboratory animal-monitoring system

In a subset of 8–10 mice per group, habitual ambulatory, rearing and total activity, oxygen consumption (VO_2_) and carbon dioxide production (VCO_2_) of individual mice were monitored over a 24-h period (12 h of light and 12 h of dark) using a CLAMS equipped with an Oxymax Open Circuit Calorimeter System (Columbus Instruments). Ambulatory, rearing and total activities were summed and analysed for light and dark periods under fed conditions. VO_2_ and VCO_2_ values were used to calculate the respiratory exchange ratio and VO_2_. Respiratory exchange ratio values were used to determine the basal metabolic rate (in kcal h^−1^ per kg).

### SA-β-Gal assay of tissue and histological evaluation

For SA-β-Gal staining, frozen sections were dried at 37 °C for 20–30 min before being fixed for 15 min at room temperature. Frozen sections were washed three times with PBS and incubated with SA-β-Gal staining solution (Beyotime) overnight at 37 °C. After completion of SA-β-Gal staining, sections were stained with eosin for 1–2 min, rinsed under running water for 1 min, differentiated in 1% acid alcohol for 10–20 s and washed again under running water for 1 min. Sections were dehydrated in increasing concentrations of alcohol and cleared in xylene. After drying, samples were examined under a bright-field microscope.

Frozen prostate, lung, liver and kidney sections stained with SA-β-Gal were quantified using ImageJ software (NIH) to measure the SA-β-Gal^+^ area. The total area was quantified by the eosin-positive area, while relative quantities of SA-β-Gal^+^ cells were calculated using the SA-β-Gal^+^ area divided by the total area. For statistics of the SA-β-Gal^+^ area of the lung, regions of the lung were randomly selected to be photographed, avoiding analysis of larger pulmonary blood vessels and the trachea. For statistical analysis of the SA-β-Gal-positive area of the liver, regions were randomly selected to be photographed. For statistical analysis of the SA-β-Gal^+^ area of the kidney, regions of the renal cortex were randomly selected to be photographed. Each tissue was measured over 10–18 regions.

For endogenous acidic β-galactosidase (β-Gal) staining, frozen kidney and salivary gland sections were dried at 37 °C for 20–30 min and then fixed in β-Gal staining fixative solution for 15 min at room temperature. Frozen sections were washed three times with PBS and incubated with β-Gal staining solution (Beyotime) overnight at 37 °C. Subsequent steps were similar to those of the regular SA-β-Gal staining protocol.

For IHC assays, formalin-fixed paraffin-embedded tissue blocks were cut into sections (7 µm thick), which were subjected to deparaffinisation and hydration. Antigen was retrieved in citrate buffer (0.01 M, pH 6.0) at 95 °C for 10 min, and sections were treated with blocking buffer (goat serum, 1:60 in 0.1% BSA in PBS) for 60 min at room temperature. Samples were further incubated with Avidin–Biotin (Vector Laboratories) for 15 min at room temperature. Sections were stained with primary antibody at 4 °C overnight before being incubated with biotinylated secondary antibody (Vector Laboratories) for 30 min. Finally, fluorescein-labelled avidin DCS (Vector Laboratories) was applied for 20 min. IHC staining was performed by a pathologist using a BOND RX Fully Automated Research Stainer (Leica). Slides were retrieved for 20 min using Epitope Retrieval Solution 1 (citrate, Leica) and incubated in Protein Block (Dako) for 5 min. Primary antibodies were diluted in Background Reducing Diluent (Dako) as follows: anti-p16^INK4A^ (rabbit, monoclonal, Abcam, ab108349) at 1:600, anti-cleaved caspase 3 (rabbit, polyclonal, Cell Signaling, 9661L) at 1:200 and anti-F4/80 (rat, monoclonal, Abcam, ab90247) at 1:500, except for anti-CD68 antibody (mouse, monoclonal, Dako, M0876), which was diluted in Bond Diluent (Leica) at 1:200. All primary antibodies were diluted in Background Reducing Diluent (Dako) and incubated for 15 min, and the Polymer Refine Detection System (Leica) was used. This system includes a hydrogen peroxidase block, Post Primary and Polymer Reagent, DAB and haematoxylin steps. Visualisation of immunostaining was achieved by incubating the slides for 10 min in DAB and DAB buffer (1:19 mixture) from the Bond Polymer Refine Detection System. Slides were counterstained for 5 min with Schmidt haematoxylin, followed by several rinses with 1× Bond wash buffer and distilled water. Slides were subsequently dehydrated with increasing concentrations of ethanol and cleared with xylene before being mounted in xylene-based VECTASHIELD medium (Vector Laboratories). Imaging was performed with an LSM 780 Zeiss confocal microscope.

### In vivo cytotoxicity assessment using blood tests

For routine blood examination, 100 μl fresh blood was acquired from each animal and mixed with EDTA immediately. Blood samples were analysed with Celltac Alpha MEK-6400 series haematology analysers (Nihon Kohden). For serum biochemical analyses, blood samples were collected and clotted for 2 h at room temperature or overnight at 4 °C. Samples were then centrifuged (1,000*g*, 10 min) to obtain serum. An aliquot of approximately 50 μl serum was subjected to analysis for creatinine, urea, alkaline phosphatase and alanine transaminase with the Chemistry Analyzer (Mindray). Circulating levels of haemoglobin, white blood cells, lymphocytes and platelets were evaluated using dry-slide technology on a VetTest 8008 chemistry analyser (IDEXX) as reported previously^47^.

All animal experiments were conducted in compliance with the NIH Guide for the Care and Use of Laboratory Animals (National Academies Press, 2011) and the ARRIVE guidelines and were approved by the IACUC of Shanghai Institute of Nutrition and Health, Chinese Academy of Sciences. For each preclinical regimen, animals were monitored for conditions including hypersensitivity (changes in body temperature, altered breathing and ruffled fur), body weight, mortality and changes in behaviour (that is, loss of appetite and distress) and were disposed of appropriately according to the individual pathological severity as defined by relevant guidelines.

### Measurement of lipid peroxidation

Levels of HNE–protein adducts in liver lysates prepared in RIPA buffer were measured in livers of mice using the OxiSelect HNE Adduct Competitive ELISA kit (Cell Biolabs) as formerly described^75^.

### Examination of glutathione levels

Mouse livers fixed in 5% sulfosalicylic acid were prepared and analysed to determine the concentration of reduced (GSH) and oxidized (GSSG) glutathione using the Glutathione Assay kit (Cayman Chemical) as previously described^75^. Sample absorbance was measured at a wavelength of 405 nm using a plate reader, and the ratio of GSH:GSSG was obtained for each tested tissue sample.

### Statistical analysis

Unless otherwise specified, data in figures are presented as mean ± s.d., and statistical significance was determined by unpaired two-tailed Student’s *t*-test (comparing two groups), one-way ANOVA or two-way ANOVA (comparing more than two groups with Tukey’s post hoc comparison), Pearson’s correlation coefficient test, Kruskal–Wallis log-rank test, Wilcoxon–Mann–Whitney test or Fisher’s exact test with GraphPad Prism 8.3 primed with customized parameters. Cox proportional hazards regression model and multivariate Cox proportional hazards model analyses were performed with SPSS software. Investigators were blinded to allocation during most experiments and outcome assessments. We used baseline body weight to assign mice to experimental groups to achieve similar body weights between groups, so that only randomisation within groups matched by body weight was conducted.

To determine sample size, we set the values of type 1 error (*α*) and power (1 − *β*) to be statistically adequate: 0.05 and 0.80, respectively^76^. We then determined *n* on the basis of the smallest effect that we measured. If the required sample size was too large, we chose to reassess the objectives or to more tightly control experimental conditions to reduce variance. For all statistical tests, *P* values <0.05 were considered significant, with *P* values presented as follows throughout the article: NS, *P* > 0.05; *P < 0.05; **P < 0.01; ***P < 0.001; ****P < 0.0001.

### Reporting Summary

Further information on research design is available in the Nature Research Reporting Summary linked to this article.

## Supplementary information

Supplementary Information Supplementary Figs. 1–6 and Tables 1–6.

Reporting Summary.

## Source data

Source Data Fig. 1 Statistical source data.

Source Data Fig. 2 Statistical source data.

Source Data Fig. 3 Statistical source data.

Source Data Fig. 3 Unprocessed western blots.

Source Data Fig. 4 Statistical source data.

Source Data Fig. 4 Unprocessed western blots.

Source Data Fig. 5 Statistical source data.

Source Data Fig. 6 Statistical source data.

Source Data Fig. 7 Statistical source data.

Source Data Fig. 8 Statistical source data.

Source Data Extended Data Fig. 1 Statistical source data.

Source Data Extended Data Fig. 2 Statistical source data.

Source Data Extended Data Fig. 2 Unprocessed western blots.

Source Data Extended Data Fig. 4 Statistical source data.

Source Data Extended Data Fig. 5 Statistical source data.

Source Data Extended Data Fig. 5 Unprocessed western blots.

Source Data Extended Data Fig. 6 Statistical source data.

Source Data Extended Data Fig. 7 Statistical source data.

Source Data Extended Data Fig. 8 Statistical source data.

Source Data Extended Data Fig. 9 Statistical source data.

## Data Availability

Source data are provided with this paper. Data that support the plots in this paper and other findings of this study are available from the corresponding author upon reasonable request. RNA-seq data generated in the present study were deposited in the Gene Expression Omnibus database under accession codes GSE156301, GSE164012 and GSE178376, respectively.
