# Editorial Expression of Concern: The flavonoid procyanidin C1 has senotherapeutic activity and increases lifespan in mice

**DOI:** 10.1038/s42255-026-01487-y

**Published:** 2026-02-23

**Authors:** Qixia Xu, Qiang Fu, Zi Li, Hanxin Liu, Ying Wang, Xu Lin, Ruikun He, Xuguang Zhang, Zhenyu Ju, Judith Campisi, James L. Kirkland, Yu Sun

**Affiliations:** 1 CAS Key Laboratory of Tissue Microenvironment and Tumour, Shanghai Institute of Nutrition and Health, University of Chinese Academy of Sciences, Chinese Academy of Sciences, Shanghai, China; 2 Institute of Health Sciences, Shanghai Jiao Tong University School of Medicine & Shanghai Institutes for Biological Sciences, Chinese Academy of Sciences, Shanghai, China; 3 Department of Pharmacology, Institute of Aging Medicine, Binzhou Medical University, Yantai, China; 4 Shanghai Institute of Nutrition and Health, Chinese Academy of Sciences, Shanghai, China; 5 Science & Technology Centre, By-Health Corp. Ltd., Guangzhou, China; 6 Key Laboratory of Regenerative Medicine of Ministry of Education, Guangzhou Regenerative Medicine and Health Guangdong Laboratory, Institute of Aging and Regenerative Medicine, Jinan University, Guangzhou, China; 7 Buck Institute for Research on Aging, Novato, CA USA; 8 Life Sciences Division, Lawrence Berkeley National Laboratory, Berkeley, CA USA; 9 Robert and Arlene Kogod Center on Aging, Mayo Clinic, Rochester, MN USA; 10 Department of Medicine and VAPSHCS, University of Washington, Seattle, WA USA

Addendum to: *Nature Metabolism* 10.1038/s42255-021-00491-8, published online 6 December 2021.

The Editor would like to alert readers that concerns were raised regarding Fig. 6b, where the CTRL Vehicle mouse appears highly similar to mouse #2 in Fig. 8c of ref. ^1^. The authors requested a correction to address this but have been unable to retrieve the underlying raw data. Readers are therefore advised to interpret these data with caution.

Qixia Xu and Yu Sun agree to this Editorial Expression of Concern. The other authors have not responded to any correspondence from the editor or publisher about this Editorial Expression of Concern.
