# Supplementary material for: The flavonoid procyanidin C1 has senotherapeutic activity and increases lifespan in mice
Source: Nat Metab. 2021 Dec 6;3(12):1706–26. doi: 10.1038/s42255-021-00491-8 (PMC8688144; doi:10.1038/s42255-021-00491-8)
Supplement: Supplementary file 1 — Supplementary Figs. 1–6 and Tables 1–6. [file 42255_2021_491_MOESM1_ESM.pdf]

nature portfolio

https://doi.org/10.1038/s42255-021-00491-8

Supplementary information

The flavonoid procyanidin C1 has senotherapeutic activity and increases lifespan in mice

In the format provided by the authors and unedited

Supplementary Information for

The flavonoid procyanidin C1 has senotherapeutic activity and increases lifespan in mice

Qixia Xu, Qiang Fu, Zi Li, Hanxin Liu, Ying Wang, Xu Lin, Ruikun He, Xuguang Zhang, Zhenyu Ju, Judith Campisi, James L. Kirkland and Yu Sun

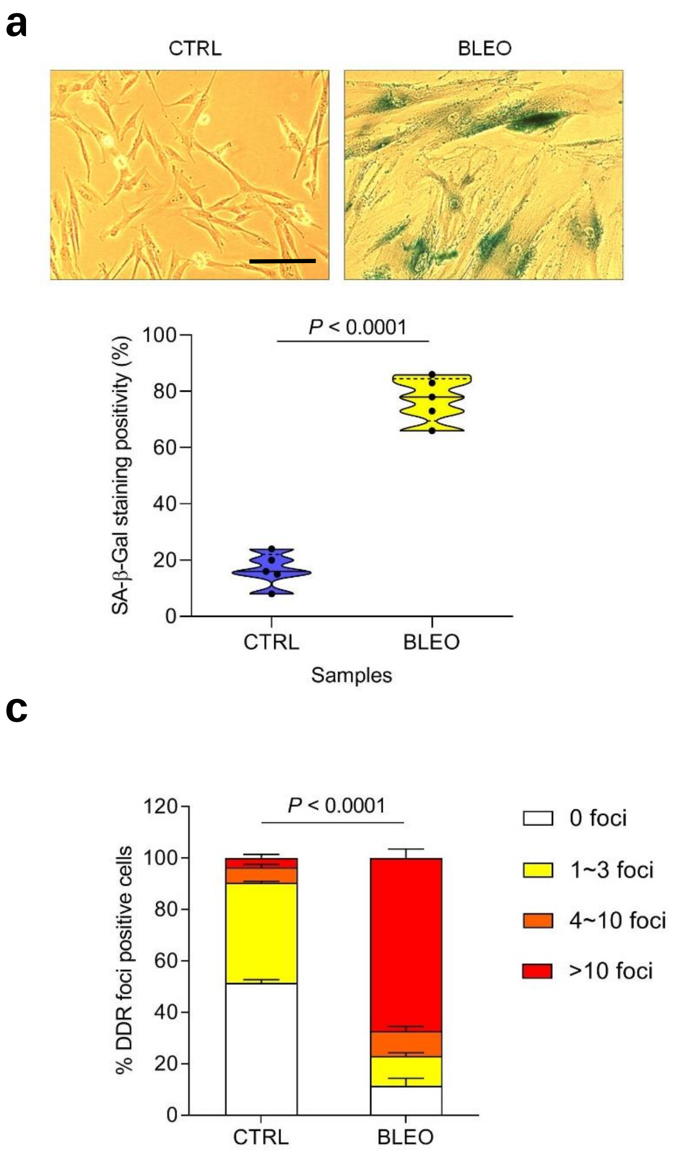

Supplementary Fig. 1. Characterization of cellular senescence induced by a chemotherapeutic agent.

(**a**) SA-β-Gal staining of PSC27 cells upon bleomycin (BLEO)-induced senescence, a form of TIS (therapy-induced senescence). Upper, representative images. Scale bar, 20 μm. Lower, statistics. (**b**) BrdU staining of PSC27 cells upon BLEO-induced TIS. Upper, representative images. Scale bar, 20 μm. Lower, statistics. (**c**) Comparative statistics of DDR foci. According to the number of DDR foci *per* cells (γH2AX staining), data were organized into 4 individual categories. Data in **a**-**c** are representative of 3 independent biological replicates, with *P* values calculated by two-sided *t*-test (**a** and **b** bottom) or two-way ANOVA with Tukey's multiple-comparison test (**c**).

Supplementary Fig. 2

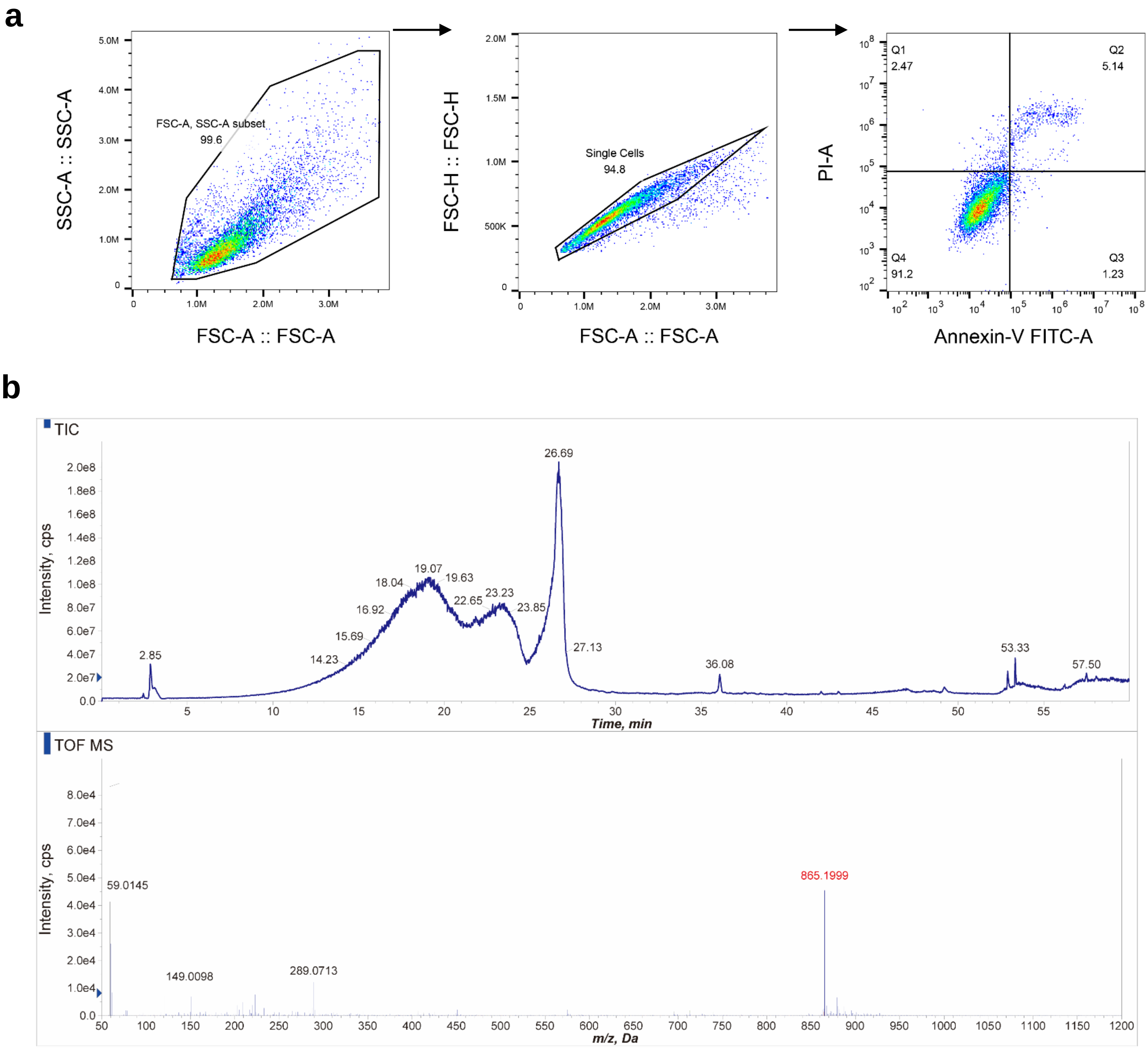

Supplementary Fig. 2. Gating strategy for analysis of senescent cell apoptosis and high resolution mass spectra for commercial PCC1.
(**a**) Scheme of gating strategy for FACS-sorting of primary human prostate stromal cells (PSC27) treated under different conditions. Total cells were initially gated to exclude debris, with FSC-A versus SSC-A applied to the gate for lymphocytes (interpreted as main cells as target). Cells were gated on single cells using FSC-A versus FSC-H, and the resulting data were used for PI and Annexin V-FITC sorting to make the subsequent gates. All samples in the same experiments and comparisons were gated with the same parameters. Viability was calculated as the percentage of PI and Annexin V double-negative cells (G4). Apoptotic cells were evaluated as Annexin V-positive cells (G2 and G3). (**b**) High resolution mass spectra showing the total ion chromatogram (TIC, upper) and base peak chromatogram (BPC, lower) of a chemically pure sample procyanidin C1 (PCC1) after performance of HPLC-ESI-QTOF-MS.

Supplementary Fig. 3

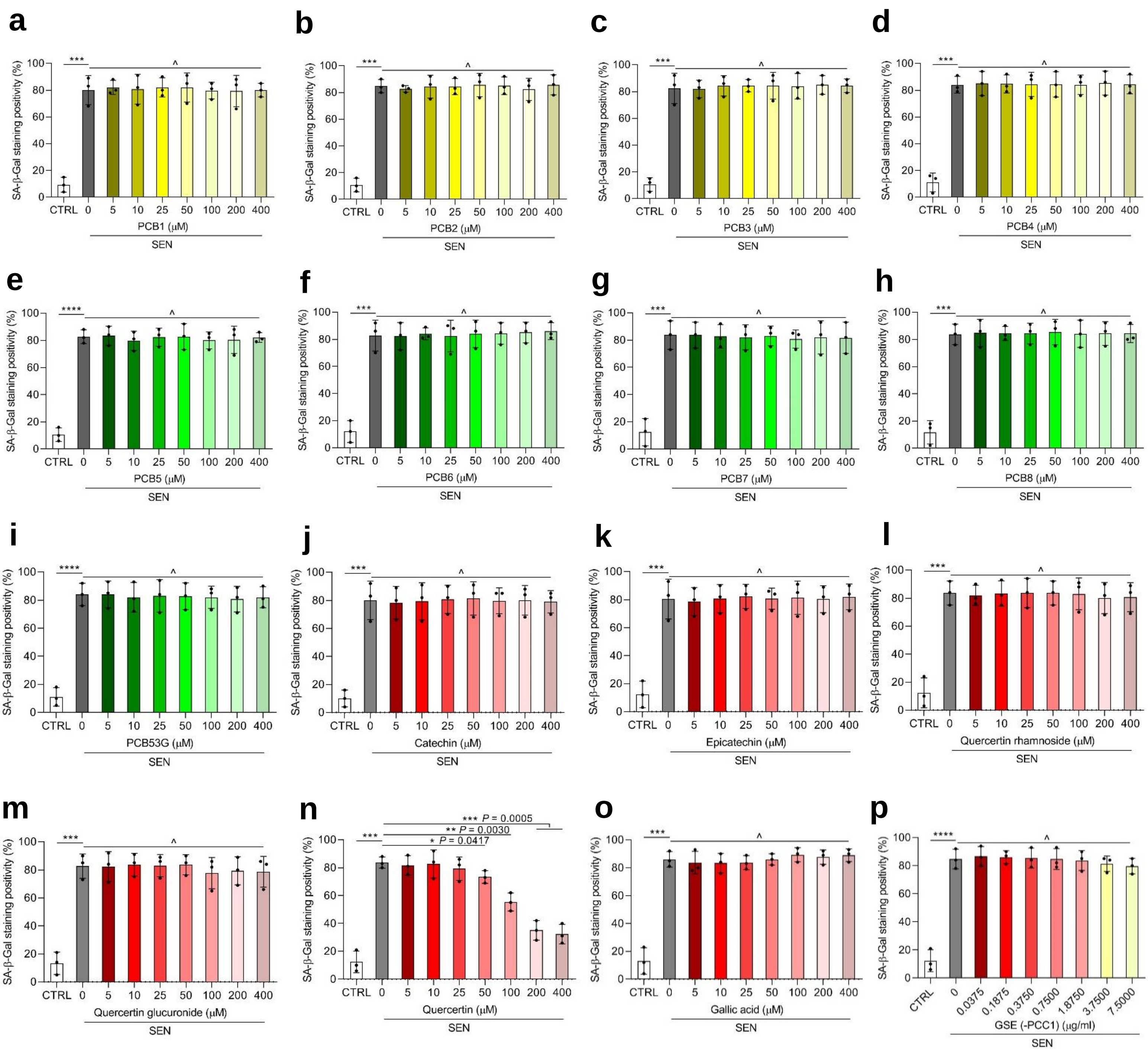

Supplementary Fig. 3. Profiling of senescent cell survival upon treatment by individual components of natural GSE.

(**a**) Quantification of senescent PSC27 cell survival by SA-β-Gal staining positivity. Procyanidin B1 (PCB1) was applied to media at increasing concentrations (up to 400 μM). CTRL, control (proliferating) cells. SEN, senescent cells. (**b**-**o**) Similar assays for procyanidin B2 (PCB2) (**b**), procyanidin B3 (PCB3) (**c**), procyanidin B4 (PCB4) (**d**), procyanidin B5 (PCB5) (**e**), procyanidin B6 (PCB6) (**f**), procyanidin B7 (PCB7) (**g**), procyanidin B8 (PCB8) (**h**), procyanidin B5-3'-gallate (PCB53G) (**i**), catechin (**j**), epicatechin (**k**), quercertin rhamnoside (**l**), quercertin glucuronide (**m**), quercertin (**n**), gallic acid (**o**). (**p**) Experimental assay for a mixture of the vast majority of GSE components by admixing individual phytochemical constituents (without PCC1) according to their weight percentage (w/w) in the natural GSE sample. Data are shown as mean $\pm$ SD and derive from 3 biological replicates (n = 3 independent assays). For all datasets, P values were calculated by two-sided t-test (between CTRL and 0 μM) or one-way ANOVA with Tukey's multiple-comparison test (between 0 μM and other concentrations *per* agent). ^, $P > 0.05$. *, $P < 0.05$. **, $P < 0.01$. ***, $P < 0.001$. ****, $P < 0.0001$.

Supplementary Fig. 4

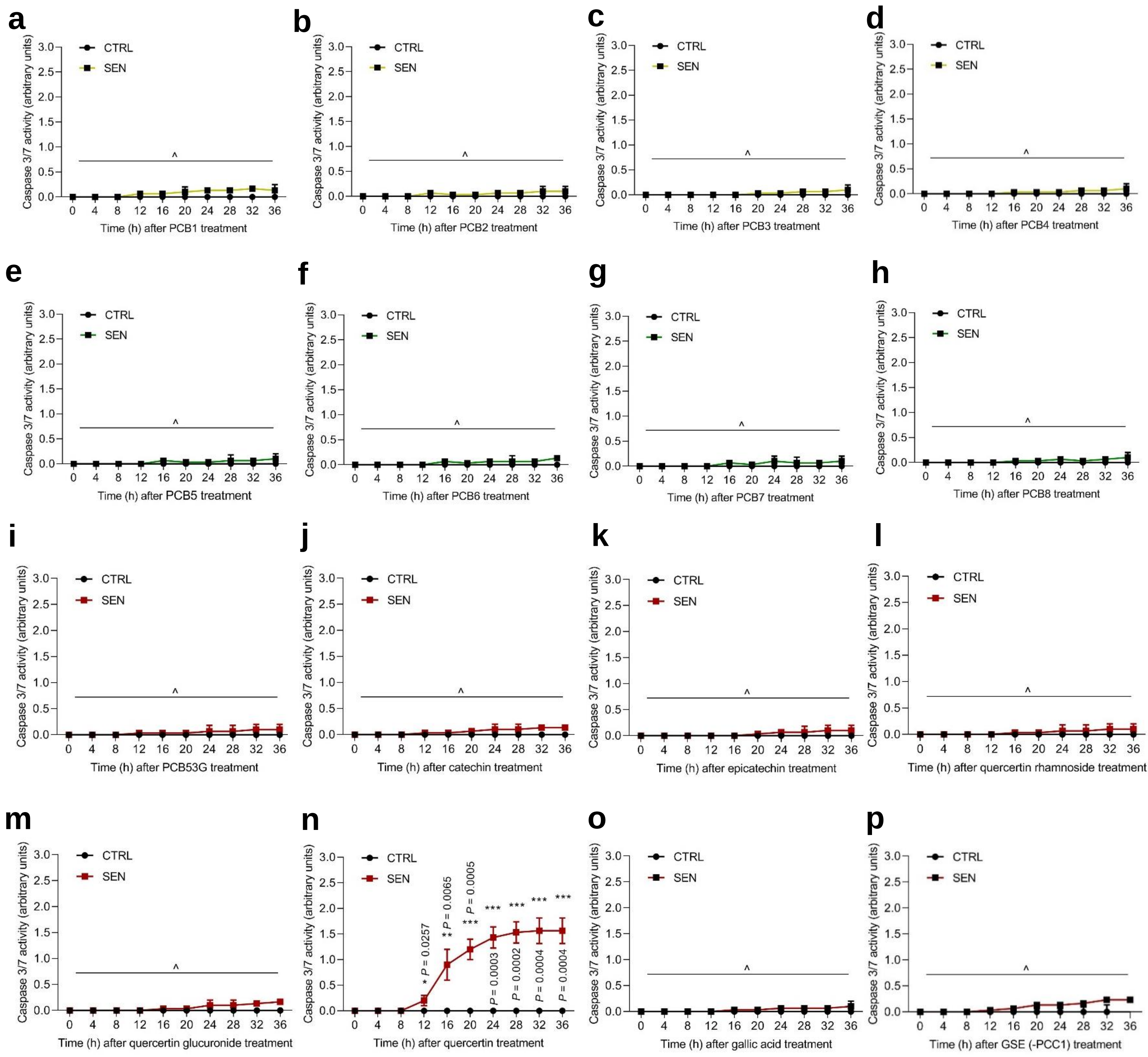

Supplementary Fig. 4. Apoptosis evaluation of cells exposed to individual components of natural GSE.

(**a**) Apoptotic assay of PSC27 cells treated by PCB1 via examination of caspase 3/7 activity. (**b**-**o**) Similar assays for procyanidin B2 (PCB2) (**b**), procyanidin B3 (PCB3) **(c)**, procyanidin B4 (PCB4) (**d**), procyanidin B5 (PCB5) (**e**), procyanidin B6 (PCB6) (**f**), procyanidin B7 (PCB7) (**g**), procyanidin B8 (PCB8) (**h**), procyanidin B5-3'-gallate (PCB53G) (**i**), catechin (**j**), epicatechin (**k**), quercertin rhamnoside (**l**), quercertin glucuronide (**m**), quercertin (**n**), gallic acid (**o**). (**p**) A similar analysis for a mixture of the vast majority of GSE components by admixing individual phytochemical constituents (without PCC1) according to their weight percentage (w/w) in the natural GSE sample. Data are shown as mean ± SD and derive from 3 biological replicates (*n* = 3 independent assays). For all datasets, *P* values were calculated by one-way ANOVA with Tukey's multiple-comparison test or calculated by two-sided *t*-test (quercetin). ^, $P > 0.05$. *, $P < 0.05$. **, $P < 0.01$. ***, $P < 0.001$.

a

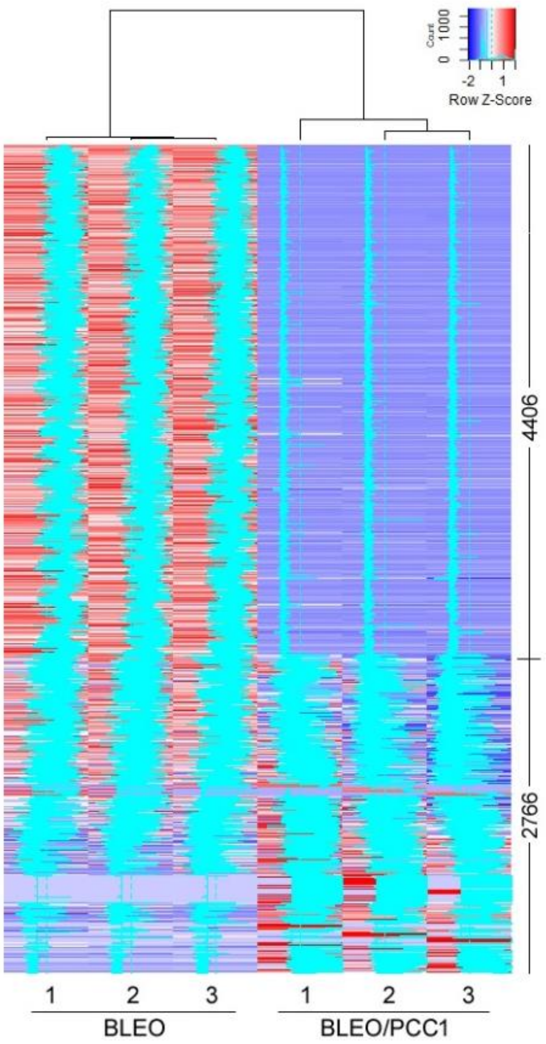

Supplementary Fig. 5. Bioinformatics depicting the expression profile of senescent cells exposed to a sublethal dose of PCC1

(**a**) Heatmap displaying human genes, the expression of which was significantly upregulated (4406) or downregulated (2766) upon treatment of senescent PSC27 cells by PCC1 (25 μM). Data derived from triplicate samples *per* group.

Supplementary Fig. 6

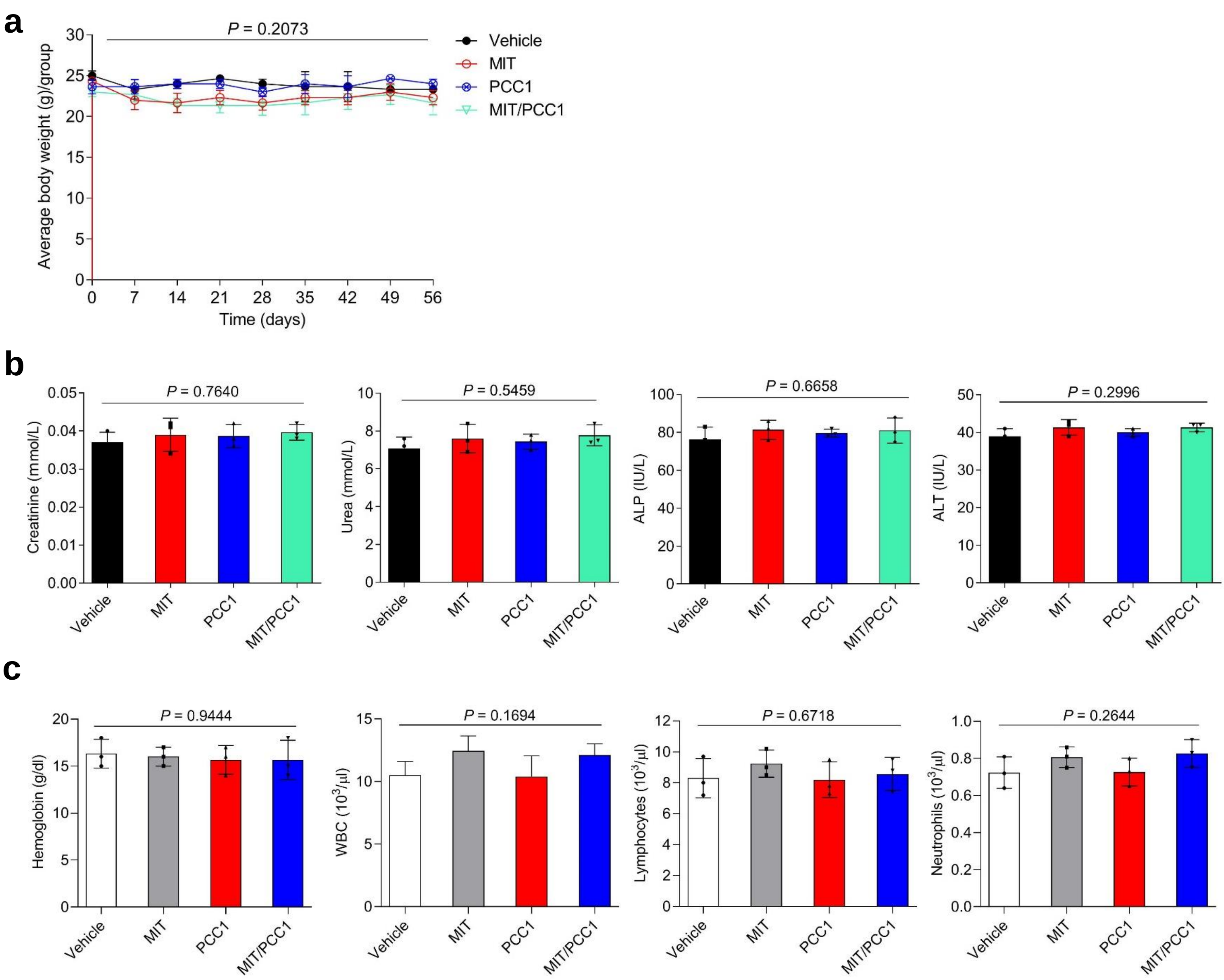

Supplementary Fig. 6. Pathophysiological assessment of treatment effects on immunocompetent mice (males).

(**a**) Animal body weight determination performed on a weekly basis for immunocompetent C57BL/6J mice. (**b**) Serum measurement of creatinine, urea, alkaline phosphatase (ALP), and alanine aminotransferase (ALT) with terminal bleeds (cardiac punctures) taken at the end of therapeutic regimens. (**c**) Routine analysis of peripheral blood. The circulating levels of hemoglobin, white blood cells, lymphocytes and platelets at the end of each therapeutic regimen were assessed. Data are shown as mean ± SD and representative of 3 independent experiments. MIT, mitoxantrone. WBC, white blood count. For all datasets, n = 3 *per* treatment arm. For all datasets, *P* values were calculated by one-way ANOVA with Tukey's multiple-comparison test.

Supplementary Table 1. Phytochemical composition of individual polyphenols and other compounds identified in GSE

Peak No.	*Compound*	*Percentage (w/w)*	*Molecular Formula*
1	Procyanidin B3	3.2	$C_{30}H_{26}O_{12}$
2	Procyanidin B1	0.8	$C_{30}H_{26}O_{12}$
3	Catechin	4.9	$C_{15}H_{14}O_{6}$
4	Procyanidin B4	1.9	$C_{30}H_{26}O_{12}$
5	Procyanidin B2	3.4	$C_{30}H_{26}O_{12}$
6	Epicatechin	6.1	$C_{15}H_{14}O_{6}$
7	Procyanidin C1	6.3	$C_{45}H_{38}O_{18}$
8	Procyanidin B5-3'-gallate	14.2	$C_{37}H_{30}O_{16}$
9	Procyanidin B5	18.4	$C_{30}H_{26}O_{12}$
10	Procyanidin B6	2.6	$C_{30}H_{26}O_{12}$
11	Procyanidin B7	2.1	$C_{30}H_{26}O_{12}$
12	Procyanidin B8	3.3	$C_{30}H_{26}O_{12}$
13	Quercertin hexoside	0.4	$C_{21}H_{20}O_{12}$
14	Quercertin rhamnoside	0.8	$C_{21}H_{20}O_{11}$
15	Quercertin glucuronide	2.2	$C_{21}H_{18}O_{13}$
16	Quercertin	0.9	$C_{15}H_{10}O_{7}$
17	Gallic acid	3.0	$C_{7}H_{6}O_{5}$
18	Ellagic acid	0.2	$C_{14}H_{6}O_{8}$
19	Phloretin	0.3	$C_{15}H_{14}O_{5}$
20	Sucrose	1.2	$C_{12}H_{22}O_{11}$

Supplementary Table 2. The Significance analysis related to data in Fig. 2b and Extended Data Fig. 4b,d.

Student's *t*-test (unpaired two-tailed)	Summary	Adjusted *P* Value
PCC1 - 0 μM		
CTRL vs. Bleomycin-induced senescence (TIS)	^	0.7599
CTRL vs. Replication-induced senescence (RS)	^	> 0.9999
CTRL vs. Oncogene-induced senescence (OIS)	^	0.9996
PCC1 - 10 μM		
CTRL vs. Bleomycin-induced senescence (TIS)	^	0.1175
CTRL vs. Replication-induced senescence (RS)	^	0.5367
CTRL vs. Oncogene-induced senescence (OIS)	^	0.6604
PCC1 - 25 μM		
CTRL vs. Bleomycin-induced senescence (TIS)	^	0.1125
CTRL vs. Replication-induced senescence (RS)	^	0.0913
CTRL vs. Oncogene-induced senescence (OIS)	^	0.4631
PCC1 - 50 μM		
CTRL vs. Bleomycin-induced senescence (TIS)	**	0.0079
CTRL vs. Replication-induced senescence (RS)	**	0.0012
CTRL vs. Oncogene-induced senescence (OIS)	**	0.0023
PCC1 - 100 μM		
CTRL vs. Bleomycin-induced senescence (TIS)	****	< 0.0001
CTRL vs. Replication-induced senescence (RS)	****	< 0.0001
CTRL vs. Oncogene-induced senescence (OIS)	****	< 0.0001
PCC1 - 200 μM		
CTRL vs. Bleomycin-induced senescence (TIS)	****	< 0.0001
CTRL vs. Replication-induced senescence (RS)	****	< 0.0001
CTRL vs. Oncogene-induced senescence (OIS)	****	< 0.0001

Supplementary Table 3. The Significance analysis by two-way ANOVA (comparing more than two groups) related to data of Extended Data Fig. 4f-h.

Supplementary Table 3. WI38 + PCC1

Two-way ANOVA	Ordinary
Alpha	0.05

Tukey's multiple comparisons test	Summary	Adjusted P Value
PCC1 - 0 μM		
CTRL vs. TIS	^	0.9989
CTRL vs. RS	^	> 0.9999
CTRL vs. OIS	^	0.9992
PCC1 - 10 μM		
CTRL vs. TIS	^	0.3739
CTRL vs. RS	^	0.4685
CTRL vs. OIS	^	0.0559
PCC1 - 25 μM		
CTRL vs. TIS	^	0.8474
CTRL vs. RS	^	0.5462
CTRL vs. OIS	^	0.4342
PCC1 - 50 μM		
CTRL vs. TIS	^	0.0935
CTRL vs. RS	*	0.0414
CTRL vs. OIS	^	0.0242
PCC1 - 75 μM		
CTRL vs. TIS	*	0.0216
CTRL vs. RS	**	0.007
CTRL vs. OIS	**	0.006
PCC1 - 100 μM		
CTRL vs. TIS	****	< 0.0001
CTRL vs. RS	***	0.0003
CTRL vs. OIS	***	0.0009
PCC1 - 125 μM		
CTRL vs. TIS	***	0.0004
CTRL vs. RS	****	< 0.0001
CTRL vs. OIS	**	0.0013
PCC1 - 150 μM		
CTRL vs. TIS	***	0.0006
CTRL vs. RS	****	< 0.0001
CTRL vs. OIS	**	0.0011
PCC1 - 175 μM		
CTRL vs. TIS	***	0.0004
CTRL vs. RS	****	< 0.0001
CTRL vs. OIS	***	0.0008
PCC1 - 200 μM		
CTRL vs. TIS	***	0.0004
CTRL vs. RS	****	< 0.0001
CTRL vs. OIS	***	0.0005

Supplementary Table 3. HUVEC + PCC1

Two-way ANOVA	Ordinary
Alpha	0.05

Tukey's multiple comparisons test	Summary	Adjusted P Value
PCC1 - 0 µM		
CTRL vs. TIS	*	> 0.9999
CTRL vs. RS	*	> 0.9999
CTRL vs. OIS	*	> 0.9999
PCC1 - 10 µM		
CTRL vs. TIS	*	0.8662
CTRL vs. RS	*	0.6291
CTRL vs. OIS	*	0.3552
PCC1 - 25 µM		
CTRL vs. TIS	*	0.8722
CTRL vs. RS	*	0.4147
CTRL vs. OIS	*	0.4194
PCC1 - 50 µM		
CTRL vs. TIS	*	0.0301
CTRL vs. RS	^	0.0563
CTRL vs. OIS	*	0.0202
PCC1 - 75 µM		
CTRL vs. TIS	*	0.0232
CTRL vs. RS	**	0.0093
CTRL vs. OIS	**	0.0062
PCC1 - 100 µM		
CTRL vs. TIS	***	0.0003
CTRL vs. RS	***	0.0002
CTRL vs. OIS	***	0.0008
PCC1 - 125 µM		
CTRL vs. TIS	***	0.0006
CTRL vs. RS	****	< 0.0001
CTRL vs. OIS	**	0.0011
PCC1 - 150 µM		
CTRL vs. TIS	***	0.0006
CTRL vs. RS	****	< 0.0001
CTRL vs. OIS	**	0.0011
PCC1 - 175 µM		
CTRL vs. TIS	***	0.0006
CTRL vs. RS	****	< 0.0001
CTRL vs. OIS	**	0.0011
PCC1 - 200 µM		
CTRL vs. TIS	***	0.0006
CTRL vs. RS	****	< 0.0001
CTRL vs. OIS	**	0.0011

Supplementary Table 3. MSC + PCC1

Two-way ANOVA	Ordinary
Alpha	0.05

Tukey's multiple comparisons test	Summary	Adjusted P Value
PCC1 - 0 µM		
CTRL vs. TIS	*	> 0.9999
CTRL vs. RS	*	> 0.9999
CTRL vs. OIS	*	> 0.9999
PCC1 - 10 µM		
CTRL vs. TIS	*	0.3465
CTRL vs. RS	*	0.1633
CTRL vs. OIS	*	0.1795
PCC1 - 25 µM		
CTRL vs. TIS	*	0.6702
CTRL vs. RS	*	0.2879
CTRL vs. OIS	*	0.1583
PCC1 - 50 µM		
CTRL vs. TIS	*	0.0445
CTRL vs. RS	*	0.0283
CTRL vs. OIS	*	0.0489
PCC1 - 75 µM		
CTRL vs. TIS	*	0.0149
CTRL vs. RS	**	0.0098
CTRL vs. OIS	**	0.0064
PCC1 - 100 µM		
CTRL vs. TIS	****	< 0.0001
CTRL vs. RS	***	0.0003
CTRL vs. OIS	***	0.0008
PCC1 - 125 µM		
CTRL vs. TIS	***	0.0004
CTRL vs. RS	****	< 0.0001
CTRL vs. OIS	**	0.0011
PCC1 - 150 µM		
CTRL vs. TIS	***	0.0002
CTRL vs. RS	****	< 0.0001
CTRL vs. OIS	**	0.001
PCC1 - 175 µM		
CTRL vs. TIS	***	0.0005
CTRL vs. RS	****	< 0.0001
CTRL vs. OIS	***	0.0005
PCC1 - 200 µM		
CTRL vs. TIS	***	0.0005
CTRL vs. RS	****	< 0.0001
CTRL vs. OIS	***	0.0007

Supplementary Table 4. List of DNA oligos for generation of constructs encoding gene-specific shRNAs.

Target name		*Primers*	*Sequences (5'-3')*
NOXA	shRNA #1	F	CCGGTGGAAGTCGAGTGTGCTACTCCTCGAG GAGTAGCACACTCGACTTCCATTTTTG
		R	AATTCAAAAATGGAAGTCGAGTGTGCTACTCC TCGAGGAGTAGCACACTCGACTTCCA
	shRNA #2	F	CCGGCAGGAACCTGACTGCATCAAACTCGAG TTTGATGCAGTCAGGTTCCTGTTTTTG
		R	AATTCAAAAACAGGAACCTGACTGCATCAAAC TCGAGTTTGATGCAGTCAGGTTCCTG
	shRNA #3	F	CCGGGCTTCTGTTCAGATGATCTTTCTCGAGA AAGATCATCTGAACAGAAGCTTTTTG
		R	AATTCAAAAAGCTTCTGTTCAGATGATCTTTCT CGAGAAAGATCATCTGAACAGAAGC
HRK	shRNA #1	F	CCGGCAAGGCGCTAGGCGACGAGCTCTCGA GAGCTCGTCGCCTAGCGCCTTGTTTTTG
		R	AATTCAAAAACAAGGCGCTAGGCGACGAGCT CTCGAGAGCTCGTCGCCTAGCGCCTTG
	shRNA #2	F	CCGGCGAGCTGCACCAGCGCACCATCTCGA GATGGTGCGCTGGTGCAGCTCGTTTTTG
		R	AATTCAAAAACGAGCTGCACCAGCGCACCAT CTCGAGATGGTGCGCTGGTGCAGCTCG
	shRNA #3	F	CCGGGCTAGGCGACGAGCTGCACCACTCGA GTGGTGCAGCTCGTCGCCTAGCTTTTTG
		R	AATTCAAAAAGCTAGGCGACGAGCTGCACCA CTCGAGTGGTGCAGCTCGTCGCCTAGC
PUMA	shRNA #1	F	CCGGGAGGGTCCTGTACAATCTCATCTCGAG ATGAGATTGTACAGGACCCTCTTTTTG
		R	AATTCAAAAAGAGGGTCCTGTACAATCTCATC TCGAGATGAGATTGTACAGGACCCTC
	shRNA #2	F	CCGGCGTGAAGAGCAAATGAGCCAACTCGAG TTGGCTCATTTGCTCTTCACGTTTTTG
		R	AATTCAAAAACGTGAAGAGCAAATGAGCCAAC TCGAGTTGGCTCATTTGCTCTTCACG
	(hRNA #3	F	CCGGGTACAATCTCATCATGGGACTCTCGAG AGTCCCATGATGAGATTGTACTTTTTG
		R	AATTCAAAAAGTACAATCTCATCATGGGACTC TCGAGAGTCCCATGATGAGATTGTAC
	shRNA #1	F	CCGGCGAAAGCTTCAGTGCATTGCACTCGAG TGCAATGCACTGAAGCTTTCGTTTTTG
		R	AATTCAAAAACGAAAGCTTCAGTGCATTGCAC TCGAGTGCAATGCACTGAAGCTTTCG

BMF	shRNA #2	F	CCGGCCTTGCTTTGAATGGAGAAGACTCGAGTCTTCTCCATTCAAAGCAAGGTTTTTG
		R	AATTCAAAAACCTTGCTTTGAATGGAGAAGACTCGAGTCTTCTCCATTCAAAGCAAGG
	shRNA #3	R	CCGGCCAGAGGAACTCAGTTAAGAACTCGAGTTCTTAACTGAGTTCCTCTGGTTTTTG
		R	AATTCAAAAACCAGAGGAACTCAGTTAAGAACTCGAGTTCTTAACTGAGTTCCTCTGG
Scramble		F	CCGGCAACAAGATGAAGAGCACCAACTCGAGTTGGTGCTCTTCATCTTGTTGTTTTTG
		R	AATTCAAAAACAACAAGATGAAGAGCACCAACTCGAGTTGGTGCTCTTCATCTTGTTG

Supplementary Table 5. A list of agents in the natural product library screened in this study.

No.	***Agent name***
1	NADH
2	NMN
3	Coenzyme Q10
4	Pyrroloquinoline quinone
5	Pine bark extract
6	Snow lotus herb
7	D-ribose
8	L-carnitine
9	Resveratrol
10	Alpha-lipoic acid (thiotic acid)
11	Olive fruit extract
12	Green tea extract
13	Cinnamon extract
14	Curcumin
15	Astaxanthin
16	Astragalus extract
17	Hesperidin
18	Grape seed extract
19	Lycopene
20	Melatonin
21	Danshen (Salvianolic acid A)
22	Quercetin
23	Ganoderma lucidum
24	Ginkgo biloba extract (GBE)
25	Chinese wolfberry
26	Centella asiatica
27	Taurine
28	Vaccinium extract
29	Broccoli seed extract
30	Sialic acid

31	Osmanthus fragrans powder
32	Cordyceps militaris powder
33	Black wolfberry frozen dried powder
34	Rattan tea instant powder
35	Olive leaf extract
36	Orange olive leaf extract
37	Green ma dai tea extract
38	Carob bean extract
39	Pomegranate powder extract
40	Sea cucumber powder
41	Crystal tomato
42	α-ketoglutaric acid
43	Fisetin
44	Baole fruit powder
45	D-(-)-3-Hydroxybutyrate sodium
46	Piperlongumine

Supplementary Table 6. A list of primer sequences for qRT-PCR assays.

Target name	***Forward (5'-3')***	***Reverse (5'-3')***
IL6	TACCCCCAGGAGAAGATTCC	TTTTCTGCCAGTGCCTCTTT
CXCL8	GTGCAGTTTTGCCAAGGAGT	CTCTGCACCCAGTTTTCCTT
IL1α	AATGACGCCCTCAATCAAAG	TGGGTATCTCAGGCATCTCC
IL1β	GGGCCTCAAGGAAAAGAATC	TTCTGCTTGAGAGGTGCTGA
CXCL1	AGGGAATTCACCCCAAGAAC	TGGATTTGTCACTGTTCAGCA
CXCL3	GCAGGGAATTCACCTCAAGA	GGTGCTCCCCTTGTTCAGTA
MMP3	GCAGTTTGCTCAGCCTATCC	GAGTGTCGGAGTCCAGCTTC
GM-CSF	CCCCAGTCACCTGCTGTTAT	TGGAATCCTGAACCCACTTC
BCL-2	GAGGATTGTGGCCTTCTTTG	ACAGTTCCACAAAGGCATCC
BCL-XL	TCGTCCATTCGTCTATGACGC	TGGATGTAGTTCCGGAAGG
BCL-W	GAGCCATATAGTTCCTTGGGA	TAGAATAAGTGGGGAGTGGGA
BAK	TGAGTACTTCACCAAGATTCA	AGTCAGGCCATGCTGGTAGAC
BAX	TTTGCTTCAGGGTTTCATCC	CAGTTGAAGTTGCCGTCAGA
BIM	GCCGCCACTACCACCACTT	AACCGAATACCGCGATGATG
NOXA	ACCAAGCCGGATTTGCGATT	ACTTGCACTTGTTCCTCGTGG
PUMA	GACGACCTCAACGCACAGTA	CACCTAATTGGGCTCCATCT
HRK	AGCAACAGGTTGGTGAAAACC	AGCATTGGGGTGTCTGTTTCT
BMF	CCCTCCTTCCCAATCGAGTC	CAGTAGGCTCTGGGCAAACA
RPL13A	GTACGCTGTGAAGGCATCAA	CGCTTTTTCTTGTCGTAGGG
p16 (Mm)	TCAACTACGGTGCAGATTCG	TCGCACGATGTCTTGATGTC
p21 (Mm)	CGGTGGAACTTTGACTTCGT	CAGGGCAGAGGAAGTACTGG
Il6 (Mm)	AGTTGCCTTCTTGGGACTGA	TCCACGATTTCCCAGAGAAC
Il1α (Mm)	AAGTCTCCAGGGCAGAGAGG	CTGATTCAGAGAGAGATGGTCA A
Il1β (Mm)	GCCCATCCTCTGTGACTCAT	AGGCCACAGGTATTTTGTCG
Mmp3 (Mm)	CAGACTTGTCCCGTTTCCAT	GGTGCTGACTGCATCAAAGA
Mmp9 (Mm)	CGTCGTGATCCCCACTTACT	AACACACAGGGTTTGCCTTC
Csf2 (Mm)	GGCCTTGGAAGCATGTAGAG	CCGTAGACCCTGCTCGAATA
Cxcl1 (Mm)	GCTGGGATTCACCTCAAGAA	TGGGGACACCTTTTAGCATC
Cxcl3 (Mm)	ATCCAGAGCTTGACGGTGAC	GGACTTGCCGCTCTTCAGTA
Ccl3 (Mm)	ATGAAGGTCTCCACCACTGC	CCCAGGTCTCTTTGGAGTCA
Ccl2 (Mm)	AGGTCCCTGTCATGCTTCTG	TCTGGACCCATTCCTTCTTG
Areg (Mm)	CTGGCAGTGAACTCTCCACA	GGCAGTGCATGGATTCTTTT
Rps16	GGCAATGGTCTCATCAAGGT	TCTCCTTCTTGGAAGCCTCA

(Mm)		
Gapdh (Mm)	AACTTTGGCATTGTGGAAGG	ACACATTGGGGGTAGGAACA

Supplementary Methods

Vector construction.

The pLKO.1-Puro plasmid was purchased from Addgene. Small hairpin RNAs were cloned for generation of knockdown constructs (shRNAs) (oligos listed in Supplementary Table 4).

SA-β-galactosidase staining, BrdU incorporation and apoptosis assays.

SA-β-Gal staining assay was conducted with a SA-β-Gal staining kit (Beyotime) according to the manufacturer's instructions. Alternatively, cells were incubated with X-Gal staining solution (1 mg/ml X-Gal, Thermo Scientific) with 5 mM $K_3[Fe(CN)_6]$ and 5 mM $K_4[Fe(CN)_6]$ for 12-16 h at 37°C. The percentage of SA-β-Gal positive cells was estimated by counting at least 100 cells per replicate sample facilitated by the "point picker" tool in ImageJ software (NIH). Flow SA-β-Gal staining assay was performed by flow cytometry using an ImaGene Green C_{12}FDG lacZ gene expression kit from Molecular Probes (Life Technologies), according to the manufacturer's instructions.

To examine the proliferative or senescence status of cells, a BrdU incorporation assay (10 μM, Yeasen) was performed for 16-18 h before cells were detected with an anti-BrdU antibody (1:400 dilution, Cell Signaling, cat no. 5292), counterstained with DAPI and assessed by immunofluorescence and high content analysis microscopy. For crystal violet staining, cells were seeded at low density in 6-well dishes and fixed at the end of the treatment with 0.5% glutaraldehyde (w/v). The plates were then stained with 0.2% crystal violet (w/v).

For apoptosis assays, target cells (proliferating or senescent) were plated in 12-well plates at 1.2×10^5 cells/well. Twenty-four h after seeding, cells were treated with specific concentrations of geroprotective agents (ABT-263 at 1.25 μM, ABT-

737 at 10 μM, Selleckchem; GSE at 0.75 μg/ml, BY-HEALTH; or PCC1 *at 100* μM, TargetMol or MedChemExpress) for 72 h. Survival was determined based on the percentage of remaining adherent cells using PrestoBlue reagent (A13262, Life Technologies) relative to control (DMSO-treated) cells.

To counteract apoptotic activity, a potent pan-caspase inhibitor QVD-OPh (MedChemExpress) was added in culture media at concentration of 1 μM before addition of geroprotective agents.

HPLC-QTOF-MS/MS analysis.

HPLC-QTOF-MS/MS (high performance liquid chromatograph conjugated with quadrupole time-of-flight-tandem mass spectrometer) analysis was performed on Nexera X2 LC-40 (SHIMADZU) coupled to AB SCIEX TripleTOF 6600 LC/MS/MS system (SCIEX), data processed with Analyst TF (v 1.7.1). Briefly, the chromatographic separation was carried out on an Accucore C30 column (2.6 um, 250 × 2.1 mm, Thermo) at room temperature. The mobile phase solvent comprises 1% acetic acid in water (solvent A) and 100% methanol (solvent B). The multistep linear gradient solvent system started with 5% B and increased to 15% B (5 min), 35% B (30 min), 70% B (45 min), 70% B (49 min) and 100% B (50 min), held at 100% B for 10 min, and decreasing to 5% B (60 min). At the initial and last gradient step, the column was equilibrated and maintained or washed for 10 min with 5% B. The flow rate was 0.2 ml/min, while the injection volume of samples was 10 μl. Detection was performed using a TripleTOF 6600 qTOF mass spectrometer (AB SCIEX) equipped with an ESI interface in negative ion mode. Operation conditions of the ESI source were as follows: capillary voltage, -4000 V; drying gas, 60 (arbitrary units); nebulization gas pressure, 60 psi; capillary temperature, 650℃; and collision energy, 30. The mass spectra were scanned from 50 to 1200 m/z, at an acquisition rate 3 spectra per second. Data acquisition and analysis were performed using Analyst TF (AB SCIEX, v 1.7.1) software.

IncuCyte analysis.

PSC27 cells were plated in 96-well dishes and induced to undergo senescence using BLEO treatment at 50 μg/ml. GSE and PCC1 were added at 0.75 μg/ml and 100 μM, respectively. Cell culture medium was supplemented with IncuCyte NucLight Rapid Red reagent for cell labelling (Essen Bioscience) and IncuCyte caspase-3/7 reagent for apoptosis (Essen Bioscience). Four images per well were collected every 4 h for 3 d.

Mitochondrial isolation.

Stromal cells cultured under regular conditions or exposed to drugs were subjected to lysis and acquisition of mitochondria using a Cell Mitochondria Isolation Kit (Beyotime) following the manufacturer's protocol. Briefly, 1×10^8 cells were harvested and washed twice with ice-cold PBS, the pellets were resuspended in 600 μl ice-cold buffer A (20 mM HEPES [pH 7.5], 1.5 mM $MgCl_2$, 10 mM KCl, 1 mM EDTA, 1 mM ethylene glycol bis (2-aminoethyl ether) tetraacetic acid, 1 mM dithiothreitol, 0.1 mM phenylmethanesulfonyl fluoride, and 1% protease inhibitor cocktail) for 10 mins and then 200 μl 1 M sucrose were added to achieve a final concentration of 250 mM. Thereafter, the cell suspension was passed five times through a 26-gauge needle fitted to a syringe to quickly crush cells, breaking at least 80% of the cells. Large plasma membrane pieces, nuclei, and unbroken cells were removed by centrifuging at 1,000 × *g* at 4°C for 10 mins. The supernatant was centrifuged at 10,000 × *g* at 4°C for 20 mins. The pellet (mitochondria) was rinsed with the above buffer A containing sucrose. The supernatant was re-centrifuged at 100,000 × *g* at 4°C for 1 h to generate cytosol. Pellets of mitochondria and cytosol were kept at -80°C until later use.

Intracellular reactive oxygen species measurement.

Intracellular reactive oxygen species (ROS) levels were determined using a ROS

Assay Kit (Beyotime) which uses dichloro-dihydro-fluorescein diacetate (DCFH-DA) as a probe. Briefly, cells were cultured in 6-well plates for 24 h at 37°C and were then washed twice with serum-free medium. Medium containing 10 µM DCFH-DA was added. Cells were then incubated for 20 min at 37°C, with light avoided during incubation. After incubation, the cells were washed thrice with serum-free medium, then observed and photographed using a fluorescence microscope (Nikon). The fluorescence intensity was measured using ImageJ software (v1.51, NIH).

Mitochondrial membrane potential (ΔΨm) analysis.

JC-1 probe was used to measure apoptosis through mitochondrial depolarization. Briefly, cells (80% confluent) were cultured in 96-well plates after treatment with 50 µM NPPB at 37°C for 24 h. Carbonyl cyanide 3-chlorophenylhydrazone (CCCP) was used as a control. CCCP is a chemical inhibitor of oxidative phosphorylation and decreases the potential of mitochondrial membranes. Cells were washed thrice with PBS, then 50 µl medium and 50 µl JC-1 working solution were added. The cells were incubated at 37°C for 20 min and washed thrice with pre-cooled JC-1 solution. ΔΨm was measured by the intensity of red and green fluorescence using a fluorescence microscope (Nikon), with green fluorescence indicative of JC-1 monomers. JC-1 monomers appears in cytosol after mitochondrial membrane depolarization, indicating early stage apoptosis. Red fluorescence suggests JC-1 aggregation and is located in the mitochondria. Mitochondrial depolarization implies apoptosis, as reflected by an increase in the green/red fluorescence intensity ratio. Fluorescence intensity was measured using ImageJ (v1.51, NIH).

Immunoblotting analysis.

Cells or tissues were homogenized in lysis buffer (Cell Signaling) supplemented with protease inhibitors (Sigma), with resultant lysates cleared by centrifugation (16,000 × *g* for 10 min at 4 °C). Total protein content was determined using

Coomassie Brilliant Blue G-250 Plus reagent (Pierce). Proteins were loaded to each lane on a 4-20% gradient SDS/PAGE gel and transferred to nitrocellulose membranes (Bio-Rad). Signals were developed with HRP-conjugated secondary antibodies (goat polyclonal to rabbit (or mouse)) (abcam) and SuperSignal West Pico Chemiluminescent Substrate (Pierce). Antibodies were purchased from commercial sources (see Reporting Summary).

Immunofluorescence staining.

Cells were fixed in 4% (v/v) paraformaldehyde (PFA) for 10 min and permeabilized in PBS containing 0.25% Triton X-100 for 10 min. After PBS washes, cells were blocked with PBS and Tween-20 (PBST) containing 5% BSA for 30 min. Cells were incubated with primary antibodies in 5% BSA in PBST overnight at 4 °C, then with the goat polyclonal to rabbit (or mouse) Alexa Fluor 488 or 594-conjugated secondary fluorescent antibodies (abcam) in the same buffer for 1 h in the dark. DAPI (1 µg/ml, Life Technologies) was used to counterstain nuclei. Slides were mounted with Vectashield medium. Fluorescence imaging was performed on a fluorescence microscope (Nikon Eclipse Ti S). Captured images were analyzed and processed using a Nikon DS-Ri2 fluorescence workstation and processed with NIS-Elements F4.30.01. Alternatively, a confocal microscope (Zeiss LSM 780) was used to acquire confocal images (antibodies used for this study listed in Reporting Summary).

Histology and immunohistochemistry.

Mouse tissue specimens were fixed overnight in 10% neutral-buffered formalin and processed for paraffin embedding. Standard staining with hematoxylin/eosin was performed on sections of 5-8 µm thickness cut from each specimen block. For immunohistochemistry, tissue sections were de-paraffinized and incubated in citrate buffer at 95 °C for 40 min for antigen retrieval before being incubated with primary antibodies (*e.g.*, anti-cleaved Caspase 3, 1:1000) overnight at 4 °C. After

3 washes with PBS, tissue sections were incubated with biotinylated secondary antibody (1:200 dilution, Vector Laboratories) for 1 h at room temperature then washed thrice, after which streptavidin-horseradish peroxidase conjugates (Vector Laboratories) were added and the slides incubated for 45 min. DAB solution (Vector Laboratories) was then added and the slides were counterstained with haematoxylin.

Determining senolytic activity.

Cells were fixed during days 8-10 after senescence induction and stained with 1 µg/ml DAPI for 15 min to assess cell numbers using automated microscopy. Different models of senescence were used to test senolytic compound activity in cell culture in a uniform fashion. For all senescence types induced, a 3-day course of senolytics was applied, with the percentage of cell survival being calculated by dividing the number of cells after senolytic treatment by the number of cells treated with vehicle. Alternatively, quantification of cell viability was achieved by collecting all cells and determining the percentage of viable cells by trypan blue exclusion.

Gene expression analysis by real-time PCR.

RNA from cells or tissues was extracted using TRIzol (Life Technologies) and reverse transcribed to cDNA using a MMLV Reverse Transcriptase kit (Life Technologies) following the manufacturer's instructions. Real-time PCR was performed using Platinum SYBR Green qPCR SuperMix (Life Technologies) in a StepOnePlus Real-Time PCR System (Applied Biosystems). The probes and primers were purchased from Life Technologies. RPL13A was used as an internal control, and primer sequences for relevant genes are listed in Supplementary Table 6.

Transcriptomic profiling through RNA-seq.

Total RNA was prepared from cells using a Direct-zol RNA MiniPrep Kit (Zymo Research). RNA sequencing libraries were generated using the NEBNext Ultra RNA Library Prep Kit from Illumina (NEB England BioLabs). Fragmented and randomly primed 2 × 150 bp paired-end libraries were sequenced using Illumina HiSeq X10.

Sequencing data quality were validated by FastQC (version 0.11.8, http://www.bioinformatics.babraham.ac.uk/projects/fastqc/), before being aligned to the human genome hg38 (or mouse genome mm10) reference genome by TopHat2. For differential gene expression analysis, read counts of each gene were obtained using HTSeq (version 0.11.1, https://htseq.readthedocs.io/en/release_0.11.1/). Differentially expressed genes (DEGs) between conditions were identified using the DESeq2 R package and genes with Benjamini-Hochberg corrected p-values < 0.05 were defined as differentially expressed. For PCA and unsupervised clustering, the read counts were normalized using rlog from DESeq2 and subjected to analysis by the Ingenuity Pathways Analysis (IPA) program (http://www.ingenuity.com/index.html). Heatmaps were generated using heatmap.2 function available in the gplots R package. Raw data were preliminarily analyzed on the free online Majorbio I-Sanger Cloud Platform (www.i-sanger.com), and subsequently deposited in the NCBI Gene Expression Omnibus (GEO) database under accession codes GSE156301 and GSE164012 and GSE178376.

The leading log-fold-change of different conditions calculated by R package edgeR (version 0.38.0, https://bioconductor.org/packages/release/bioc/html/edgeR.html) was used as input for gene set enrichment analysis. The gene sets of mouse molecular signature were obtained through msigdbr (version 7.0.1, https://cran.r-project.org/web/packages/msigdbr/index.html). In this package, the original human genes of the Molecular Signatures Database (MSigDB v7.0,

http://software.broadinstitute.org/gsea/msigdb/index.jsp) were converted to non-human model organism homologous genes. GSEA was performed using GSEA desktop application version 2.2.7 with Molecular Signature Database (version 3.1), and by clusterProfiler (version 3.14.0) (https://bioconductor.org/packages/release/bioc/html/clusterProfiler.html). R (version 3.6.0, https://cran.r-project.org/) was used for gene expression analysis.

Gene ontology (GO) term enrichment analysis was performed using the DAVID 6.8 functional annotation tool. Gene lists were selected by comparing gene expression between control and senescent cells or vehicle-treated and drug-treated senescent cells using *t*-tests: fold changes > 2 and P values < 0.05. The top GO terms of GO-Biological Progress (BP) and GO-Cellular Component (CC) were selected for presentation.

Protein-protein interaction (PPI) analysis was performed with STRING 11.0 (https://string-db.org). The specific proteins meeting the criteria, were imported to NetworkAnalyst (http://www.networkanalyst.ca). A minimum interaction network was chosen for further hub and module analysis.
