# Supplementary material for: The flavonoid procyanidin C1 has senotherapeutic activity and increases lifespan in mice
Source: Nat Metab. 2021 Dec 6;3(12):1706–26. doi: 10.1038/s42255-021-00491-8 (PMC8688144; doi:10.1038/s42255-021-00491-8)
Supplement: Supplementary file 2 — Reporting Summary. [file 42255_2021_491_MOESM2_ESM.pdf]

nature research

Corresponding author(s): Yu Sun

Last updated by author(s): Oct 2, 2021

Reporting Summary

Nature Research wishes to improve the reproducibility of the work that we publish. This form provides structure for consistency and transparency in reporting. For further information on Nature Research policies, see our Editorial Policies and the Editorial Policy Checklist.

Statistics

For all statistical analyses, confirm that the following items are present in the figure legend, table legend, main text, or Methods section.

n/a	Confirmed	
☐	☒	The exact sample size (*n*) for each experimental group/condition, given as a discrete number and unit of measurement
☐	☒	A statement on whether measurements were taken from distinct samples or whether the same sample was measured repeatedly
☐	☒	The statistical test(s) used AND whether they are one- or two-sided<br>*Only common tests should be described solely by name; describe more complex techniques in the Methods section.*
☒	☐	A description of all covariates tested
☐	☒	A description of any assumptions or corrections, such as tests of normality and adjustment for multiple comparisons
☐	☒	A full description of the statistical parameters including central tendency (e.g. means) or other basic estimates (e.g. regression coefficient) AND variation (e.g. standard deviation) or associated estimates of uncertainty (e.g. confidence intervals)
☐	☒	For null hypothesis testing, the test statistic (e.g. *F*, *t*, *r*) with confidence intervals, effect sizes, degrees of freedom and *P* value noted<br>*Give P values as exact values whenever suitable.*
☒	☐	For Bayesian analysis, information on the choice of priors and Markov chain Monte Carlo settings
☒	☐	For hierarchical and complex designs, identification of the appropriate level for tests and full reporting of outcomes
☒	☐	Estimates of effect sizes (e.g. Cohen's *d*, Pearson's *r*), indicating how they were calculated

Our web collection on statistics for biologists contains articles on many of the points above.

Software and code

Policy information about availability of computer code

Data collection	IN Cell Analyzer 2500HS GE Healthcare<br>QuantStudio Real-Time PCR Software v1.1 Applied Biosystems<br>Flow cytometry: Beckman CytoFLEX LX, CytExpert (v 1.2)<br>Mass spectrometry: Nexera X2 LC-40 (SHIMADZU) coupled to AB SCIEX TripleTOF 6600 LC/MS/MS system (SCIEX) , Analyst TF (v 1.7.1)<br>Hematology assay: Celltac Alpha MEK-6400 series hematology analyzers (Nihon Kohden)<br>Microsoft Excel 2016 (version 16.16.13)
Data analysis	GraphPad Prism 8 (version 8.3.0)<br>FlowJo (v 10.6.2)<br>Image J (v 1.52p)<br>FASTQC (v 0.11.5)<br>GSEA (v 4.1.0)<br>MSigDB (v 7.4)<br>Tophat (v 2.0.11)<br>Ensembl (v 72) gene annotation<br>NetworkAnalyst (v 3.0)<br>STRING (v 11.0)<br>FUNRICH (v 3)<br>Ingenuity Pathway Analysis (v 2020.12)<br>PANTHER (v 16.0)<br><br>R packages (v 4.0.3) from CRAN and Bioconductor:<br>custom R v 4.0 scripts<br>annotate (v 1.56.2)

ensembldb (v 2.2.2)
edgeR (v 3.22.3)
fdrtool (v 1.2.16)
Bedtools2 (v 2.26.0)
Bowtie (v 2.2.2)
DESeq2 (v 1.18.1)
ggplot2 (v 3.1.0)
ggrepel (v 0.8.0)
gplots (v 3.0.1)
graph (v 1.56.0)
gridExtra (v 2.3)

pd.hta.2.0 (v 3.12.2)
pheatmap (v 1.0.10)
plotly (v 4.8.0)
plotrix (v 3.7.4)
plyr (v 1.8.4)
preseqR (v 3.1.2)

For manuscripts utilizing custom algorithms or software that are central to the research but not yet described in published literature, software must be made available to editors and reviewers. We strongly encourage code deposition in a community repository (e.g. GitHub). See the Nature Research guidelines for submitting code & software for further information.

Data

Policy information about availability of data

All manuscripts must include a data availability statement. This statement should provide the following information, where applicable:

- Accession codes, unique identifiers, or web links for publicly available datasets
- A list of figures that have associated raw data
- A description of any restrictions on data availability

Statistical source data underlying some key experiments are provided in separate Excel files. Unprocessed immunoblots are provided as source data. All data supporting the findings of the study are available from the corresponding author upon reasonable request.
Mass spectrometry data associated with this study will be submitted to the public depository (the Mass Spectrometry Interactive Virtual Environment at https://massive.ucsd.edu, which serves as a data hub for computational mass spectrometry with open mzTab format data conversion function currently in construction).
The RNA-seq data are deposited in publicly accessible sources and available at the GEO database with accession codes GSE156301, GSE164012 and GSE178376.
Fig. 3, Extended Data Fig. 2 and 6, and Supplementary Fig. 5 are associated with the RNA-seq data (raw and processed data accessible through GEO).
Extended Data Fig. 4f-h are associated with complete analysis results presented in Supplementary Table 3.
Other source data and statistics for corresponding figures are provided in Supplementary Information or Source Data sections.

Field-specific reporting

Please select the one below that is the best fit for your research. If you are not sure, read the appropriate sections before making your selection.

☒ Life sciences ☐ Behavioural & social sciences ☐ Ecological, evolutionary & environmental sciences

For a reference copy of the document with all sections, see nature.com/documents/nr-reporting-summary-flat.pdf

Life sciences study design

All studies must disclose on these points even when the disclosure is negative.

Sample size	Sample size was not determined by specific statistical methods a priori, but were based on variability of associated assays. The sample sizes are similar to those reported in previous publications with comparable experiments (Chang et al., Nature Medicine. 2016; Zhang et al., Nature Communications. 2018; Xu et al., Nature Medicine. 2018; Guerrero et al., Nature Metabolism. 2019). We did not focus on a specific effect-size and performed a discovery study, including all available samples that passed QC into analysis. To our knowledge, a comparative senescence-cell targeting senolytics investigation of plant-derived procyanidins has not been previously undertaken.
Data exclusions	All data were included, without specific exclusions.
Replication	All experiments were reproducible. Every figure states how many times the related experiment was performed with similar results. All data presented were from independent biological replicates or independent experiments. All attempts at replication were successful.
Randomization	For high-throughput data acquisition and database generation, samples were randomized between batches to account for possible batch-effect. Stringent inclusion criteria were set to account for other possible confounding variables. For preclinical experiments, animals were randomly assigned to each individual groups.
Blinding	The investigators were not blinded to sample group allocations due to the fact that the genotypes of human primary cells, needed to be carefully documented by the investigators, so blinding was not always possible during experimental setup. When feasible, data analysis was performed blind, including RNA and protein preparation, q-PCR and immunoblots, immunofluorescence staining, RNA-seq library preparation, bioinformatics profiling, evaluation of histological sections from preclinical biospecimens, for which all data acquisition was performed blinded

to types of individual samples.

Reporting for specific materials, systems and methods

We require information from authors about some types of materials, experimental systems and methods used in many studies. Here, indicate whether each material, system or method listed is relevant to your study. If you are not sure if a list item applies to your research, read the appropriate section before selecting a response.

Materials & experimental systems

n/a	Involved in the study
☐	☒ Antibodies
☐	☒ Eukaryotic cell lines
☒	☐ Palaeontology and archaeology
☐	☒ Animals and other organisms
☒	☐ Human research participants
☒	☐ Clinical data
☒	☐ Dual use research of concern

Methods

n/a	Involved in the study
☒	☐ ChIP-seq
☐	☒ Flow cytometry
☒	☐ MRI-based neuroimaging

Antibodies

Antibodies used

The following antibodies were purchased from the indicated suppliers and used for immunoblotting (if not stated; or otherwise, immunofluorescence staining or immunohistochemistry staining, as stated separately) at indicated concentrations: rabbit monoclonal anti-p-ATM (Abways cat. no. CY5111, no clone name), 1:1000; rabbit monoclonal anti-ATM (Abways cat. no. CY5207, no clone name), 1:1000; rabbit monoclonal anti-γH2AX (Cell Signaling cat. no. 9718, clone 20E3), 1:500; rabbit monoclonal anti-H2AX (Cell Signaling, cat. no. 7631, clone D17A3), 1:1000; rabbit polyclonal anti-CXCL8 (Proteintech cat. no. 27095-1-AP), 1:500; mouse monoclonal anti-p53 (Cell Signaling cat. no. 2524, clone 1C12), 1:500 (for both immunoblotting and immunofluorescence staining); rabbit polyclonal anti-Lamin A/C (Proteintech cat. no. 10298-1-AP), 1:1000; mouse monoclonal anti-BrdU (Cell Signaling cat. no. 5292, clone Bu20a), 1:1000; rabbit polyclonal anti-p-p38 (R&D cat. no. AF869), 1:2000; rabbit polyclonal anti-p38 (Cell signaling cat. no. 9212), 1:1000; rabbit polyclonal anti-p-AKT (Abcam cat. no. ab8932), 1:1000; rabbit polyclonal anti-AKT (Proteintech cat. no. 10176-1-AP), 1:500; mouse monoclonal anti-NF-κB p65 (Santa Cruz cat. no. sc-8008, clone F-6), 1:1000; rabbit polyclonal anti-NOXA (Abways cat. no. CY6774), 1:1000; rabbit polyclonal anti-PUMA (Abways cat. no. CY5460), 1:1000; rabbit polyclonal anti-Bcl-2 (Abways cat. no. CY6717), 1:1000; rabbit monoclonal anti-Bcl-xL (Cell Signaling cat. no. 2764, clone 54H6), 1:1000; rabbit polyclonal anti-Bax (Cell Signaling cat. no. 2772), 1:1000; rabbit polyclonal anti-Cytochrome c (Proteintech cat. no. 10993-1-AP), 1:1000; rabbit polyclonal anti-COX IV (ABclonal cat. no. A6564), 1:500 (for both immunoblotting and immunofluorescence staining); rabbit monoclonal anti-Caspase 3 (cleaved) (Cell Signaling cat. no. 9661, clone Asp175), 1:1000 (or 1:250 for immunohistochemistry staining); rabbit monoclonal anti-Caspase 3 (total) (Cell Signaling cat. no. 9662, no clone name), 1:1000; mouse monoclonal anti-p16 (BD Biosciences cat. no. 554079, clone G175-1239), 1:500 (for immunofluorescence staining); goat polyclonal anti-rabbit IgG H&L (HRP) (abcam cat. no. ab6721), 1:500 or goat polyclonal anti-mouse IgG H&L (HRP) (abcam cat. no. ab6789), 1:500; goat polyclonal to rabbit (or mouse) IgG Alexa Fluor 488 or 594-conjugated secondary (abcam cat. no. ab150077, ab150080, ab150113, ab150116), 1:400 (for immunofluorescence staining); mouse monoclonal anti-β-actin (Proteintech cat. no. 66009-1-Ig, clone 2D4H5), 1:4000; rabbit monoclonal anti-GAPDH (Abways cat. no. AB0037, no clone name), 1:2000.

Validation

Antibody validations were performed by antibody suppliers per quality assurance literature provided by each supplier for applications used in this study (see links below).
rabbit monoclonal anti-p-ATM (Abways cat. no. CY5111), immunoblotting
http://www.abways.com/showproduct.asp?cid=CY5111
rabbit monoclonal anti-ATM (Abways cat. no. CY5207), immunoblotting
http://www.abways.com/showproduct.asp?cid=CY5207
rabbit monoclonal anti-γH2AX (Cell Signaling cat. no. 9718), immunoblotting
https://www.cellsignal.cn/products/primary-antibodies/phospho-histone-h2a-x-ser139-20e3-rabbit-mab/9718?site-search-type=Products&N=4294956287&Ntt=9718&fromPage=plp&_requestid=6426762
rabbit monoclonal anti-H2AX (Cell Signaling cat. no. 7631), immunoblotting
https://www.cellsignal.cn/products/primary-antibodies/histone-h2a-x-d17a3-xp-rabbit-mab/7631?site-search-type=Products&N=4294956287&Ntt=7631&fromPage=plp&_requestid=2864687
rabbit polyclonal anti-CXCL8 (Proteintech cat. no. 27095-1-AP), immunoblotting
https://www.ptgcn.com/products/CXCL8-IL8-Antibody-27095-1-AP.htm
mouse monoclonal anti-p53 (Cell Signaling cat. no. 2524), immunoblotting and immunofluorescence staining
https://www.cellsignal.cn/products/primary-antibodies/p53-1c12-mouse-mab/2524?site-search-type=Products&N=4294956287&Ntt=2524&fromPage=plp&_requestid=2865145
rabbit polyclonal anti-Lamin A/C (Proteintech cat. no. 10298-1-AP), immunoblotting
http://www.ptgcn.com/products/lamin-A-Antibody-10298-1-AP.htm
mouse monoclonal anti-BrdU (Cell Signaling cat. no. 5292), immunoblotting
https://www.cellsignal.com//products/primary-antibodies/brdu-bu20a-mouse-mab/5292
rabbit polyclonal anti-p-p38 (R&D cat. no. AF869), immunoblotting
https://www.rndsystems.com/cn/products/human-mouse-rat-phospho-p38-map-kinase-t180-y182-antibody_af869
rabbit polyclonal anti-p38 (Cell Signaling cat. no. 9212), immunoblotting
https://www.cellsignal.com//products/primary-antibodies/p38-mapk-antibody/9212
rabbit polyclonal anti-p-AKT (Abcam cat. no. ab8932), immunoblotting
https://www.abcam.cn/akt1-phospho-s473-antibody-ab8932.html
rabbit polyclonal anti-AKT (Proteintech cat. no. 10176-1-AP), immunoblotting

http://www.ptgcn.com/products/AKT-Antibody-10176-2-AP.htm
mouse monoclonal anti-NF-κB p65(Santa Cruz cat. no. sc-8008), immunoblotting
https://www.scbt.com/p/nfkappab-p65-antibody-f-6?requestFrom=search
rabbit polyclonal anti-NOXA (Abways cat. no. CY6774), immunoblotting
http://www.abways.com/showproduct.asp?cid=CY6774
rabbit polyclonal anti-PUMA (Abways cat. no. CY5460), immunoblotting
http://www.abways.com/showproduct.asp?cid=CY5460
rabbit polyclonal anti-Bcl-2 (Abways cat. no. CY6717), immunoblotting
http://www.abways.com/showproduct.asp?cid=CY6717
rabbit monoclonal anti-Bcl-xL (Cell Signaling cat. no. 2764), immunoblotting
https://www.cellsignal.com//products/primary-antibodies/bcl-xl-54h6-rabbit-mab/2764
rabbit polyclonal anti-Bax (Cell Signaling cat. no. 2772), immunoblotting
https://www.cellsignal.com//products/primary-antibodies/bax-antibody/2772
rabbit polyclonal anti-Cytochrome c (Proteintech cat. no. 10993-1-AP), immunoblotting
https://www.ptglab.com/products/CYCS-Antibody-10993-1-AP.htm
rabbit polyclonal anti-COX IV (ABclonal cat. no. A6564), immunoblotting and immunofluorescence staining
https://abclonal.com.cn/catalog/A6564
rabbit monoclonal cleaved-Caspase 3 (Cell Signaling cat. no. 9661), immunoblotting and immunohistochemistry staining
https://www.cellsignal.cn/products/primary-antibodies/cleaved-caspase-3-asp175-antibody/9661
rabbit monoclonal Caspase 3 (Cell Signaling cat no. 9662), immunoblotting
https://www.cellsignal.cn/products/primary-antibodies/caspase-3-antibody/9662?site-search-type=Products&N=4294956287&Ntt=9662&fromPage=plp
mouse monoclonal anti-p16 (BD Biosciences cat. no. 554079), immunofluorescence staining
https://www.citeab.com/antibodies/2411943-554079-bd-pharmingen-purified-mouse-anti-human-p16
goat polyclonal anti-rabbit IgG H&L (HRP) (abcam cat. no. ab6721), immunoblotting
https://www.abcam.com/goat-rabbit-igg-hl-hrp-ab6721.html
goat polyclonal anti-mouse IgG H&L (HRP) (abcam cat. no. ab6789), immunoblotting
https://www.abcam.com/goat-mouse-igg-hl-hrp-ab6789.html
goat polyclonal to rabbit (or mouse) IgG Alexa Fluor 488 or 594-conjugated secondary (abcam cat. no. ab150077, ab150080, ab150113, ab150116), immunofluorescence staining
https://www.abcam.com/goat-rabbit-igg-hl-alexa-fluor-488-ab150077.html
https://www.abcam.com/goat-rabbit-igg-hl-alexa-fluor-594-ab150080.html
https://www.abcam.com/goat-mouse-igg-hl-alexa-fluor-488-ab150113.html
https://www.abcam.com/goat-mouse-igg-hl-alexa-fluor-594-ab150116.html
mouse monoclonal anti-β-actin (Proteintech cat. no. 66009-1-Ig), immunoblotting
https://www.ptgcn.com/products/Pan-Actin-Antibody-66009-1-Ig.htm
rabbit monoclonal anti-GAPDH (Abways cat. no. AB0037), immunoblotting
http://www.abways.com/showproduct.asp?cid=AB0037

Anti-p-ATM rabbit monoclonal, Abways, cat. no. CY5111. Validated by the company and the following publication: Boyi Zhang, Da Fu, Qixia Xu, et al. The senescence-associated secretory phenotype is potentiated by feedforward regulatory mechanisms involving Zscan4 and TAK1. Nat Commun. 2018 Apr 30;9(1):1723. doi: 10.1038/s41467-018-04010-4.

Anti-ATM rabbit monoclonal, Abways, cat. no. CY5207. Validated by the company and the following publication: Boyi Zhang, Da Fu, Qixia Xu, et al. The senescence-associated secretory phenotype is potentiated by feedforward regulatory mechanisms involving Zscan4 and TAK1. Nat Commun. 2018 Apr 30;9(1):1723. doi: 10.1038/s41467-018-04010-4.

Anti-γH2AX rabbit monoclonal, Cell Signaling, cat. no. 9718. Validated by the company and the following publication: Chan EM, Shibue T, McFarland JM, et al. WRN helicase is a synthetic lethal target in microsatellite unstable cancers. Nature. 2019 Apr;568(7753):551-556. doi: 10.1038/s41586-019-1102-x.

Anti-H2AX rabbit monoclonal, Cell Signaling, cat. no. 7631. Validated by the company and the following publication: Nilay S Sethi, Osamu Kikuchi, Gina N Duronio, et al. Early TP53 alterations engage environmental exposures to promote gastric premalignancy in an integrative mouse model. Nat Genet. 2020 Feb;52(2):219-230. doi: 10.1038/s41588-019-0574-9.

Anti-CXCL8 rabbit polyclonal, Proteintech, cat. no. 27095-1-AP. Validated by the company and the following publication: Zheng T, Ma G, Tang M, et al. IL-8 secreted from M2 macrophages promoted prostate tumorigenesis via STAT3/MALAT1 pathway. Int J Mol Sci. 2018 Dec 27;20(1):98. doi: 10.3390/ijms20010098.

Anti-p53 mouse monoclonal, Cell Signaling, cat. no. 2524. Validated by the company and the following publication: Hao Guo, Wei-Chun Chou, Yunjia Lai, et al. Multi-omics analyses of radiation survivors identify radioprotective microbes and metabolites. Science. 2020 Oct 30;370(6516):eaay9097. doi: 10.1126/science.aay9097.

Anti-Lamin A/C rabbit polyclonal, Proteintech cat. no. 10298-1-AP. Validated by the company and the following publication: Jiao Yang, Xiaoman Zhang, Zheling Chen, et al. Angiomotin-p130 inhibits β-catenin stability by competing with Axin for binding to tankyrase in breast cancer. Cell Death Dis. 2019 Feb 21;10(3):179. doi: 10.1038/s41419-019-1427-2.

Anti-BrdU mouse monoclonal, Cell Signaling, cat. no. 5292. Validated by the company and the following publication: Boyi Zhang, Da Fu, Qixia Xu, et al. The senescence-associated secretory phenotype is potentiated by feedforward regulatory mechanisms involving Zscan4 and TAK1. Nat Commun. 2018 Apr 30;9(1):1723. doi: 10.1038/s41467-018-04010-4.

Anti- p-p38 rabbit polyclonal, R&D cat. no. AF869. Validated by the company and the following publication: Uwe Schlomann, Garrit Koller, Catharina Conrad, et al. ADAM8 as a drug target in pancreatic cancer. Nat Commun. 2015 Jan 28;6:6175. doi: 10.1038/ncomms7175.

Anti-p38 rabbit polyclonal, Cell Signaling, cat. no. 9212. Validated by the company and the following publication: Cuicui Wang, Jie

Shen, Jun Ying, et al. FoxO1 is a crucial mediator of TGF-β/TAK1 signaling and protects against osteoarthritis by maintaining articular cartilage homeostasis. Proc Natl Acad Sci U S A. 2020 Dec 1;117(48):30488-30497. doi: 10.1073/pnas.2017056117.

Anti-p-AKT rabbit polyclonal, Abcam cat. no. ab8932. Validated by the company and the following publication: Huaying Dong, Jianguo Hu, Kejian Zou, et al. Activation of LncRNA TINCR by H3K27 acetylation promotes Trastuzumab resistance and epithelial-mesenchymal transition by targeting MicroRNA-125b in breast Cancer. Mol Cancer. 2019 Jan 8;18(1):3. doi: 10.1186/s12943-018-0931-9.

Anti-AKT rabbit polyclonal, Proteintech cat. no. 10176-1-AP. Validated by the company and the following publication: Dalong Cao, Zihao Qi, Yangyang Pang, et al. Retinoic Acid-Related Orphan Receptor C Regulates Proliferation, Glycolysis, and Chemoresistance via the PD-L1/ITGB6/STAT3 Signaling Axis in Bladder Cancer. Cancer Res. 2019 May 15;79(10):2604-2618. doi: 10.1158/0008-5472.CAN-18-3842.

Anti-NF-κB p65 mouse monoclonal, Santa Cruz cat. no. sc-8008. Validated by the company and the following publication: Jinlin Zhao, Xin Wang, Zeyun Mi, et al. STAT3/miR-135b/NF-κB axis confers aggressiveness and unfavorable prognosis in non-small-cell lung cancer. Cell Death Dis. 2021 May 14;12(5):493. doi: 10.1038/s41419-021-03773-x.

Anti-NOXA rabbit monoclonal, Abways cat. no. CY6774. Validated by the company.

Anti-PUMA rabbit monoclonal, Abways cat. no. CY5460. Validated by the company.

Anti-Bcl-2 rabbit monoclonal, Abways cat. no. CY6717. Validated by the company and the following publication: Jianing Li, Liangliang Zheng, Xue Wang, et al. Taurine protects INS-1 cells from apoptosis induced by Di(2-ethylhexyl) phthalate via reducing oxidative stress and autophagy. Toxicol Mech Methods. 2019 Jul;29(6):445-456. doi: 10.1080/15376516.2019.1588931.

Anti-Bcl-xL rabbit monoclonal, Cell Signaling cat. no. 2764. Validated by the company and the following publication: Yan Zhou, Tao Tao, Guangjie Liu, et al. TRAF3 mediates neuronal apoptosis in early brain injury following subarachnoid hemorrhage via targeting TAK1-dependent MAPKs and NF-κB pathways. Cell Death Dis. 2021 Jan 7;12(1):10. doi: 10.1038/s41419-020-03278-z.

Anti-Bax rabbit polyclonal, Cell Signaling cat. no. 2772. Validated by the company and the following publication: Valentina Montagnani, Luisa Maresca, Alessandro Apollo, et al. E3 ubiquitin ligase PARK2, an inhibitor of melanoma cell growth, is repressed by the oncogenic ERK1/2-ELK1 transcriptional axis. J Biol Chem. 2020 Nov 20;295(47):16058-16071. doi: 10.1074/jbc.RA120.014615.

Anti-Cytochrome c rabbit polyclonal, Proteintech cat. no. 10993-1-AP. Validated by the company and the following publication: Xi-Gong Li, Jun-Hua Du, Yang Lu, et al. Neuroprotective effects of rapamycin on spinal cord injury in rats by increasing autophagy and Akt signaling. Neural Regen Res. 2019 Apr;14(4):721-727. doi: 10.4103/1673-5374.247476.

Anti-COX IV rabbit polyclonal, ABclonal cat. no. A6564. Validated by the company and the following publication: Huazhang Zhu, Weizhen Zhang, Yingying Zhao, et al. GSK3β-mediated tau hyperphosphorylation triggers diabetic retinal neurodegeneration by disrupting synaptic and mitochondrial functions. Mol Neurodegener. 2018 Nov 22;13(1):62. doi: 10.1186/s13024-018-0295-z.

Anti-Caspase 3 (cleaved) rabbit monoclonal, Cell Signaling, cat. no. 9661. Validated by the company and the following publication: Zhou W, Chen C, Shi Y, et al. Targeting Glioma Stem Cell-Derived Pericytes Disrupts the Blood-Tumor Barrier and Improves Chemotherapeutic Efficacy. Cell Stem Cell. 2017 Nov 2;21(5):591-603.e4. doi: 10.1016/j.stem.

Anti-Caspase 3 (total) rabbit monoclonal, Cell Signaling, cat. no. 9662. Validated by the company and the following publication: Donato V, Bonora M, Simoneschi D, et al. The TDH-GCN5L1-Fbxo15-KBP axis limits mitochondrial biogenesis in mouse embryonic stem cells. Nat Cell Biol. 2017 Apr;19(4):341-351. doi: 10.1038/ncb3491.

Anti-p16 mouse monoclonal, BD Biosciences cat. no. 554079. Validated by the company and following publication: Yann Deleye, Alexia Karen Cotte, Sarah Anissa Hannou, et al. CDKN2A/p16INK4a suppresses hepatic fatty acid oxidation through the AMPKα2-SIRT1-PPARα signaling pathway. J Biol Chem. 2020 Dec 11;295(50):17310-17322. doi: 10.1074/jbc.RA120.012543.

Goat polyclonal anti-rabbit IgG H&L (HRP) (abcam cat. no. ab6721). Validated by the company and following publication:
Tian Y, Zhong L, Gao S, et al. LncRNA LINC00974 downregulates miR-122 to upregulate RhoA in oral squamous cell carcinoma. Cancer Biother Radiopharm. 2021 Feb;36(1):18-22. doi: 10.1089/cbr.2019.2907.

Goat polyclonal anti-mouse IgG H&L (HRP) (abcam cat. no. ab6789). Validated by the company and following publication:
Jin K, Wen Z, Wu B, et al. NOTCH-induced rerouting of endosomal trafficking disables regulatory T cells in vasculitis. J Clin Invest. 2021 Jan 4;131(1):e136042. doi: 10.1172/JCI136042.

Goat polyclonal to rabbit (or mouse) IgG Alexa Fluor 488 or 594-conjugated secondary, abcam cat. no. ab150077, ab150080, ab150113, ab150116. Validated by the company and the following publication: Boyi Zhang, Qilai Long, Shanshan Wu, et al. KDM4 orchestrates epigenomic remodeling of senescent cells and potentiates the senescence-associated secretory phenotype. Nat Aging. 2021 May;1(5):454-472. doi: 10.1038/s43587-021-00063-1.

Anti-β-actin mouse monoclonal, Proteintech cat. no. 66009-1-Ig. Validated by the company and the following publication: Damaris N Lorenzo and Vann Bennett. Cell-autonomous adiposity through increased cell surface GLUT4 due to ankyrin-B deficiency. Proc Natl Acad Sci U S A. 2017 Nov 28;114(48):12743-12748. doi: 10.1073/pnas.1708865114.

Anti-GAPDH rabbit monoclonal, Abways cat. no. AB0037. Validated by the company and the following publication: Boyi Zhang, Qilai Long, Shanshan Wu, et al. KDM4 Orchestrates Epigenomic Remodeling of Senescent Cells and Potentiates the Senescence-Associated Secretory Phenotype. Nat Aging. 2021 May;1(5):454-472. doi: 10.1038/s43587-021-00063-1.

Eukaryotic cell lines

Policy information about cell lines

Cell line source(s)	Primary normal human stromal cell line PSC27 was a kind gift (no commercial yet) of Dr. Peter Nelson (FHCRC) as described in references 14 and 15. Primary human fetal lung fibroblast line WI38 and primary human umbilical vein endothelial cell line HUVEC were purchased from ATCC, and maintained in DMEM and F-12K Medium, respectively, as recommended by the provider. Primary mesenchymal stem cell line MSC was isolated from human umbilical vein tissues and cultured in MSC complete media with 10 µg/ml recombinant human insulin as described in reference 73.
Authentication	All human cell lines were authenticated by genomic DNA profiling assays (STR) performed by XP Biomed.
Mycoplasma contamination	All cell lines in this study were tested negative for mycoplasma contamination.
Commonly misidentified lines (See ICLAC register)	None of the cell lines used in this study is present in the database of commonly misidentified cell lines.

Animals and other organisms

Policy information about studies involving animals; ARRIVE guidelines recommended for reporting animal research

Laboratory animals	Nod-obese diabetic and severe combined immunodeficiency (NOD-SCID) male mice and wild type C57BL/6J male mice (NanJing Model Animal Centry, China) of 6-8 weeks old were housed and maintained in accordance with animal guidelines of Shanghai Institute of Nutrition and Health. All experimental mice were used and housed (22-25 C, 30% humidity) under 12 h light/12 h dark cycle (6 am-6 pm) with a standard rodent chow diet (5LOD, PicoLab).
Wild animals	No wild animals were used in this study.
Field-collected samples	This study did not involve field-collected samples.
Ethics oversight	All animal experiments were conducted in compliance with NIH Guide for the Care and Use of Laboratory Animals (National Academies Press, 2011) and the ARRIVE guidelines, and were approved by the Institutional Animal Care and Use Committee (IACUC) of the University of Washington and an equivalent committee of Shanghai Institute of Nutrition and Health, Chinese Academy of Sciences.

Note that full information on the approval of the study protocol must also be provided in the manuscript.

Flow Cytometry

Plots

Confirm that:

☒ The axis labels state the marker and fluorochrome used (e.g. CD4-FITC).

☒ The axis scales are clearly visible. Include numbers along axes only for bottom left plot of group (a 'group' is an analysis of identical markers).

☒ All plots are contour plots with outliers or pseudocolor plots.

☒ A numerical value for number of cells or percentage (with statistics) is provided.

Methodology

Sample preparation	Control and/or drug-treated cells were first trypsinized from culture flasks or dishes, simply rinsed and resuspended in PBS before counted by trypan blue dye-exclusion method. Briefly, cells were resuspended in Annexin V-FITC (195 µl) to thoroughly disperse, then treated with Annexin V-FITC (5 µl) and Propidium Iodize (PI, 10 µl) to stain at room temperature, with light-shielded for 10-20 min, then incubated on ice. Samples were then sent to flow device for analysis. Annexin V-FITC gives green fluorescence, while PI emits as red signals.
Instrument	Beckman Gallios flow cytometer (Beckman, CytoFLEX LX)
Software	CytExpert (v 1.2) and FlowJo software (FlowJo 10.6.2)
Cell population abundance	Totally 5-6 million cells were used for sorting per sample.
Gating strategy	FSC-A versus SSC-A was initially applied to the gate for lymphocytes (main targets). Cells were gated on single cells using FSC-A versus FSC-H and then used for PI and Annexin V-FITC to make subsequent gates. Cells were correspondingly gated for FITC fluorescence against PI fluorescence signals. Live cells were gated as FITC-PI-(Q4), apoptotic cells were gated as FITC+PI+ (Q2) or FITC+PI-(Q3).

☒ Tick this box to confirm that a figure exemplifying the gating strategy is provided in the Supplementary Information.
