# Supplementary figures and images for: The flavonoid procyanidin C1 has senotherapeutic activity and increases lifespan in mice
Source: Nat Metab. 2021 Dec 6;3(12):1706–26. doi: 10.1038/s42255-021-00491-8 (PMC8688144; doi:10.1038/s42255-021-00491-8)

**Fig. 3g**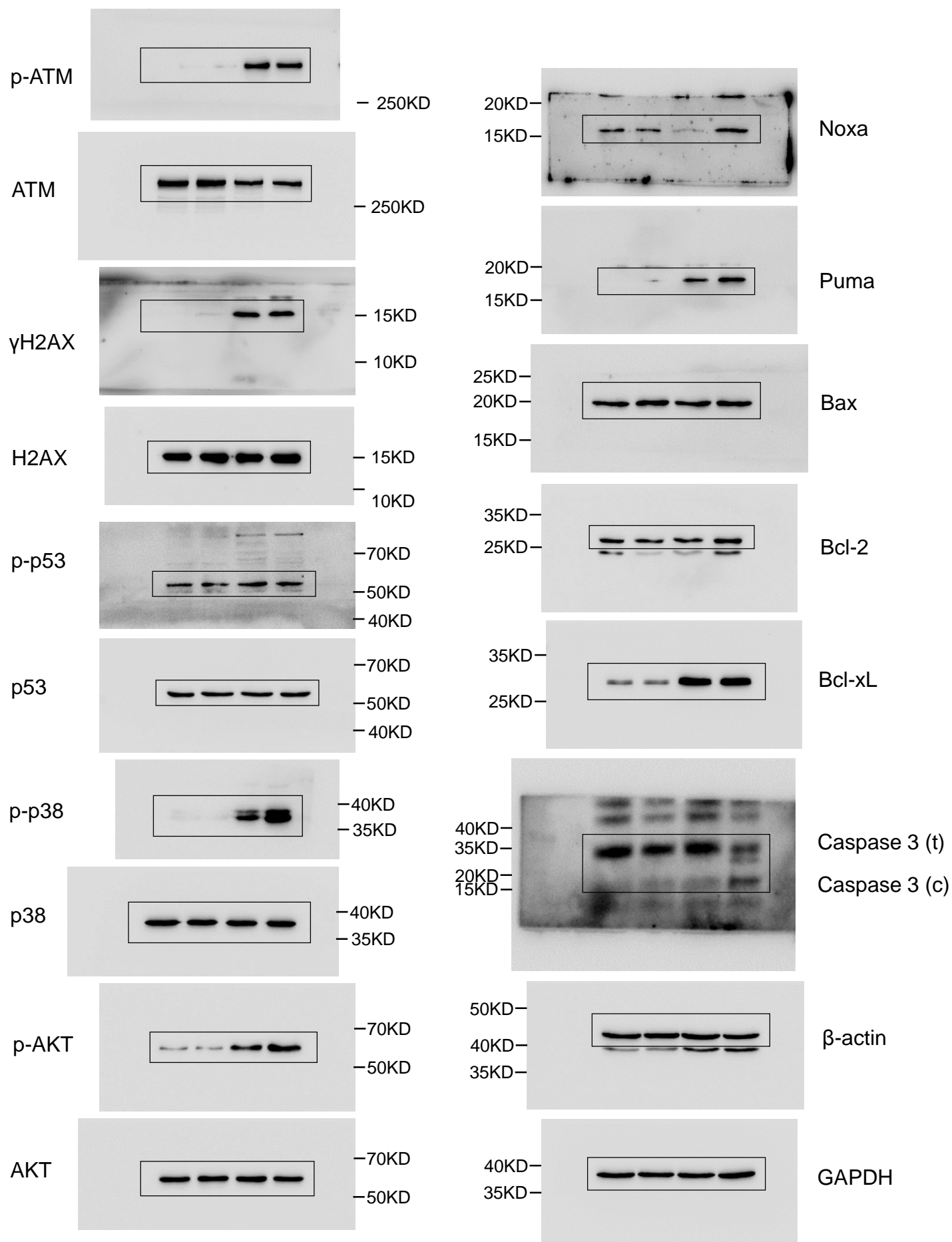

Supplement: Supplementary file 6 — Unprocessed western blots. [file 42255_2021_491_MOESM6_ESM.pdf]

**Fig. 4b**

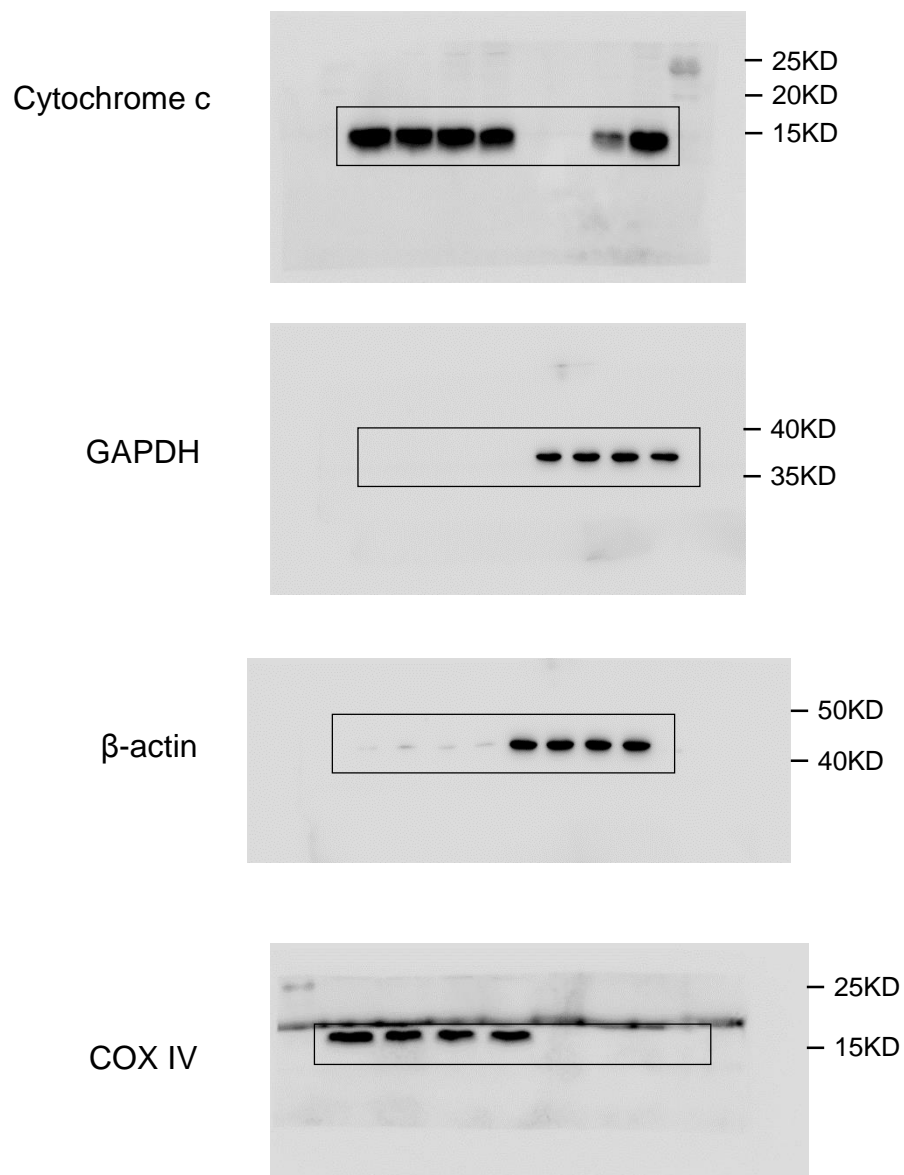

**Fig. 4e**

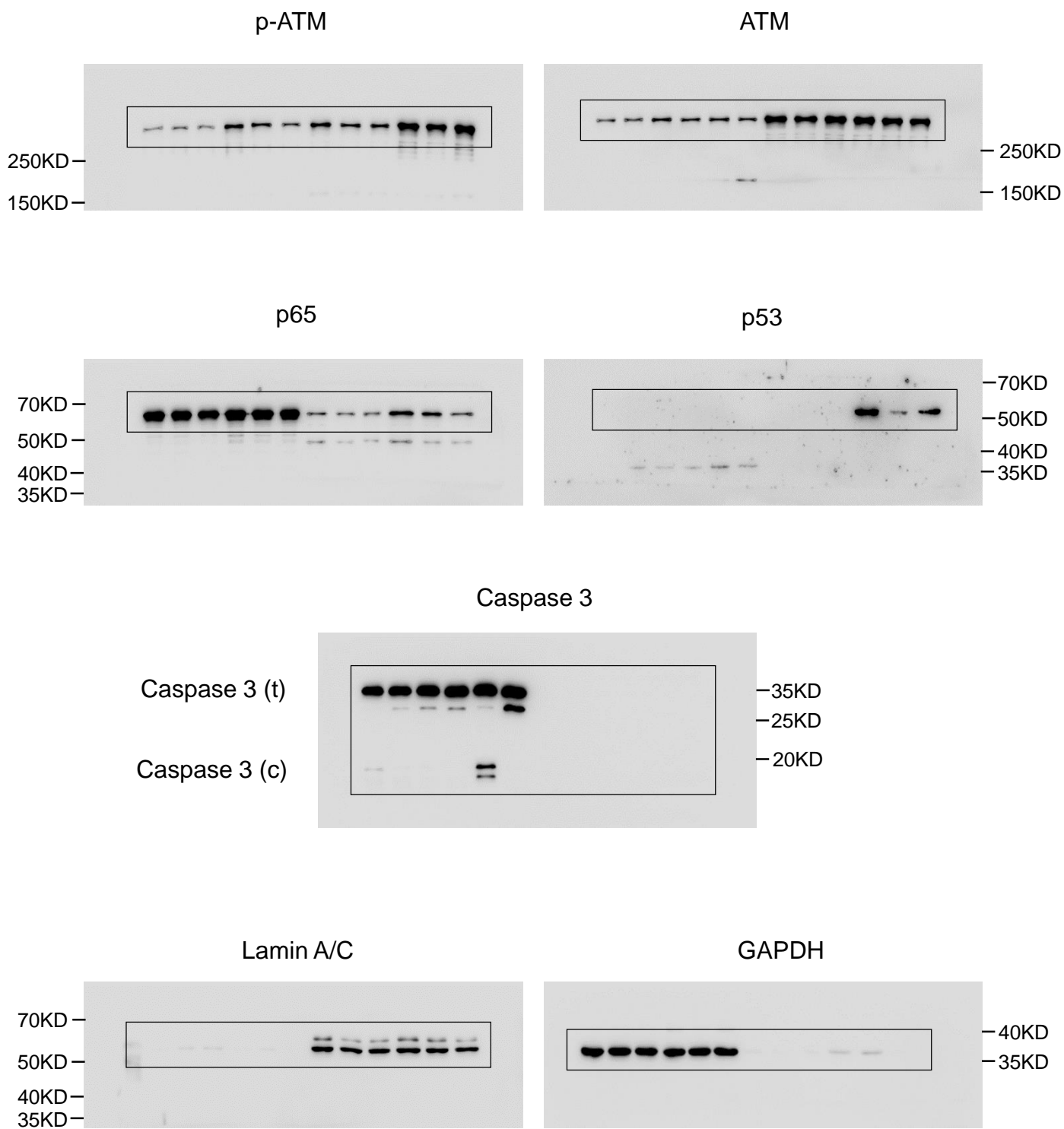

**Fig. 4g**

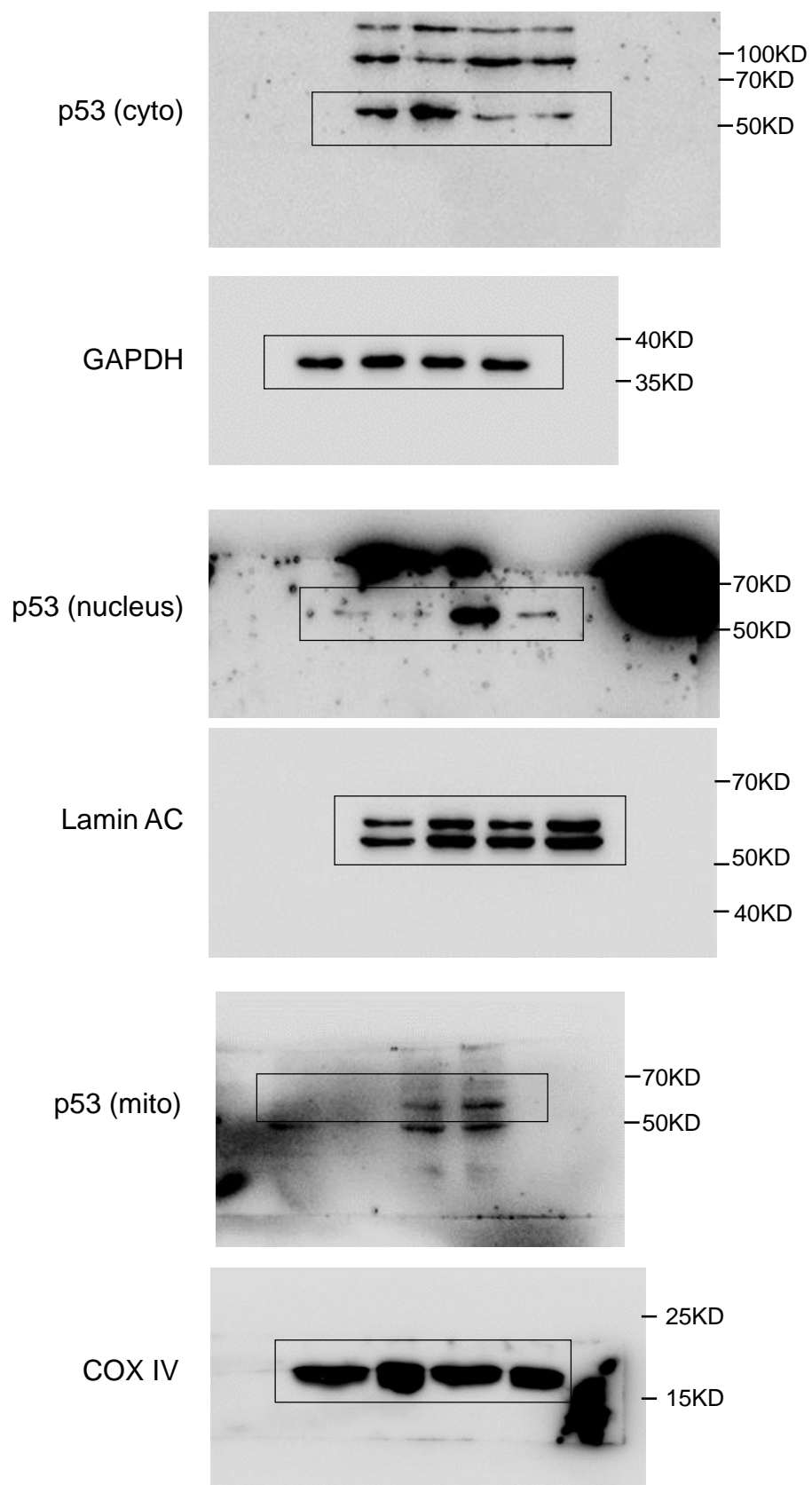

Supplement: Supplementary file 8 — Unprocessed western blots. [file 42255_2021_491_MOESM8_ESM.pdf]

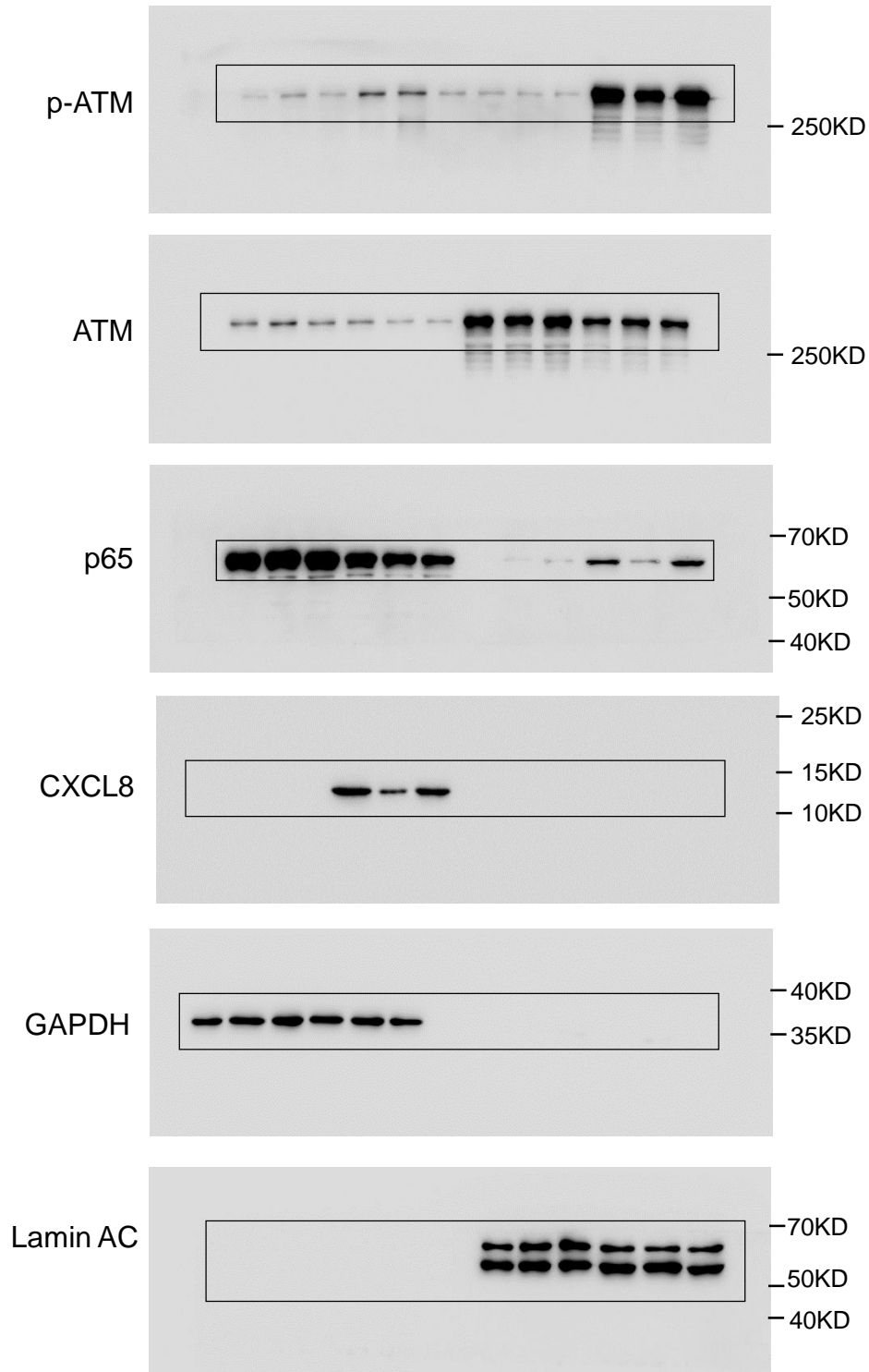

Supplement: Supplementary file 15 — Unprocessed western blots. [file 42255_2021_491_MOESM15_ESM.pdf]

**Extended Data Fig. 5b**

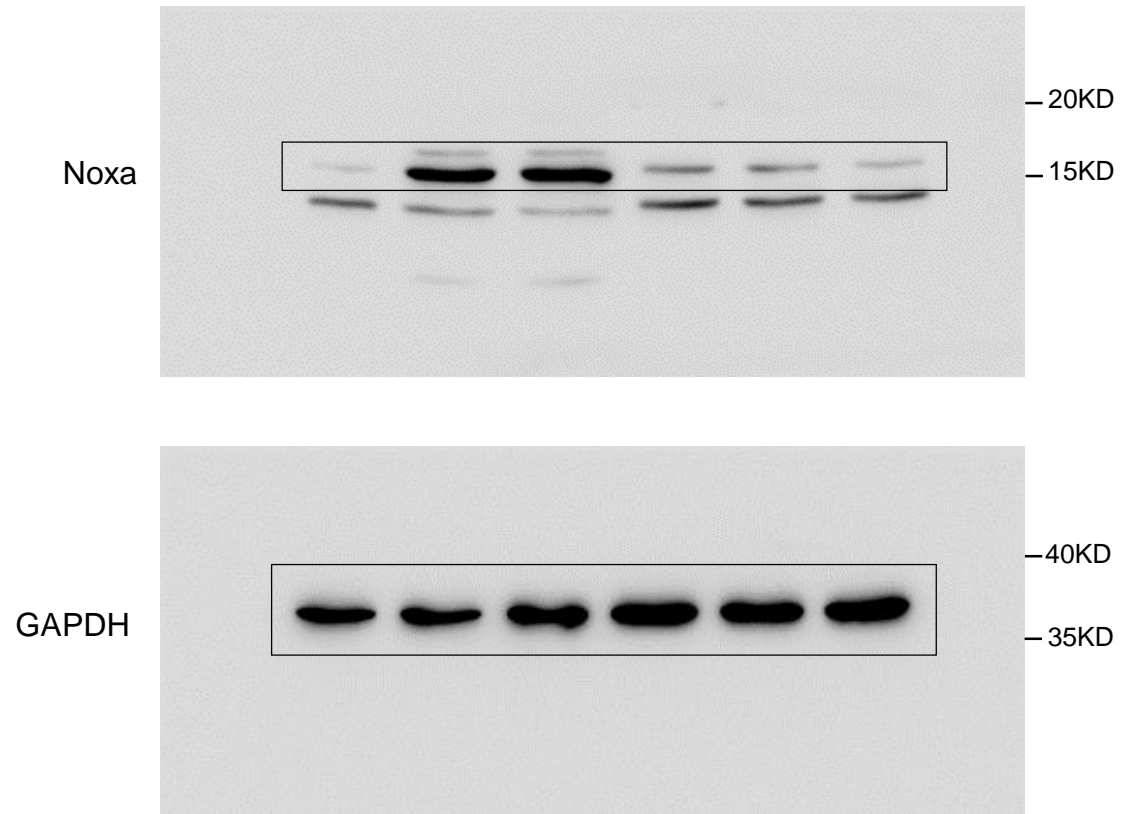

Puma

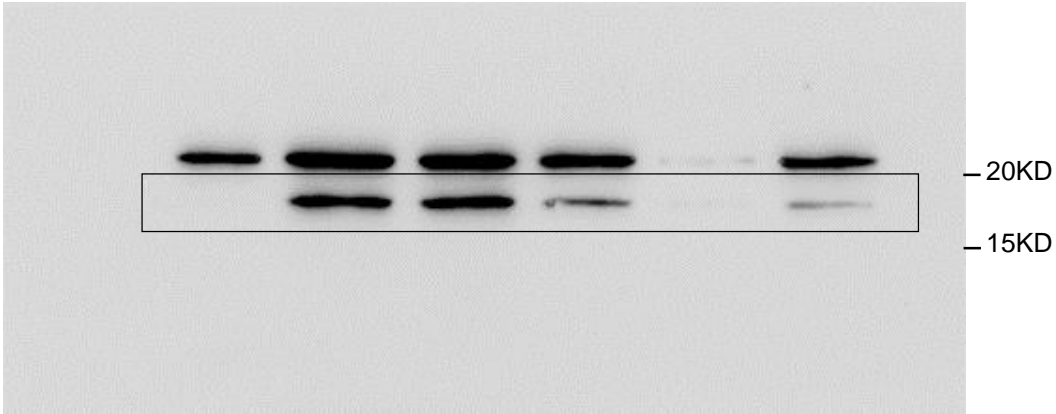

GAPDH

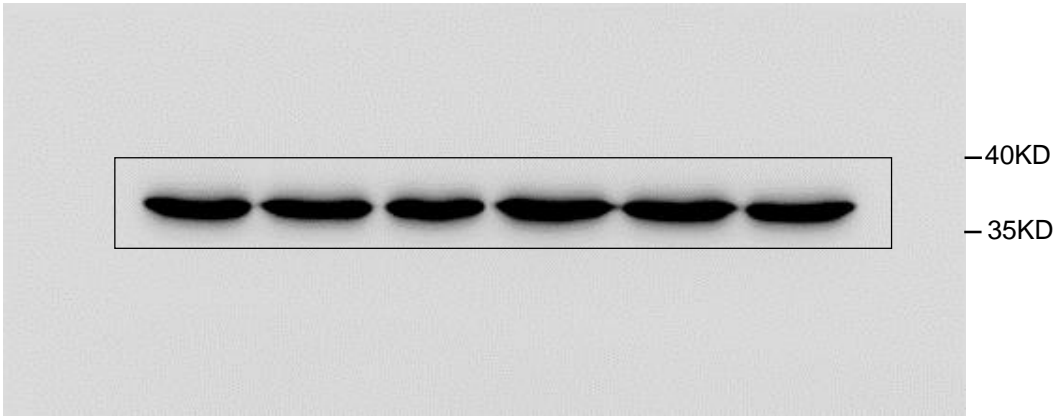

Cytochrome c

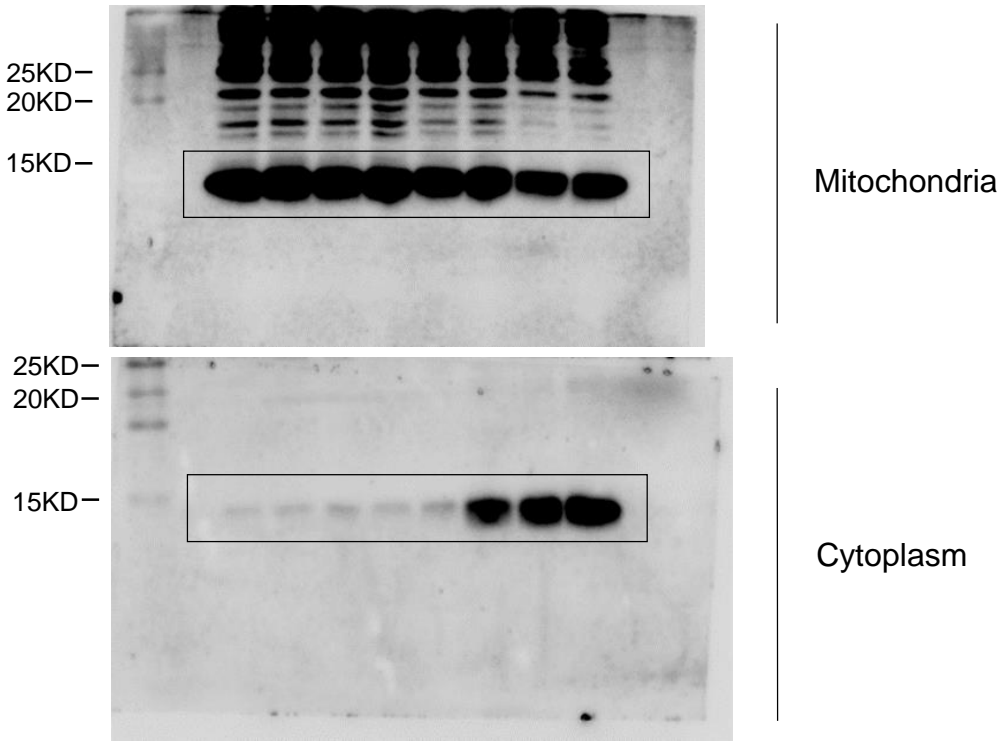

GAPDH

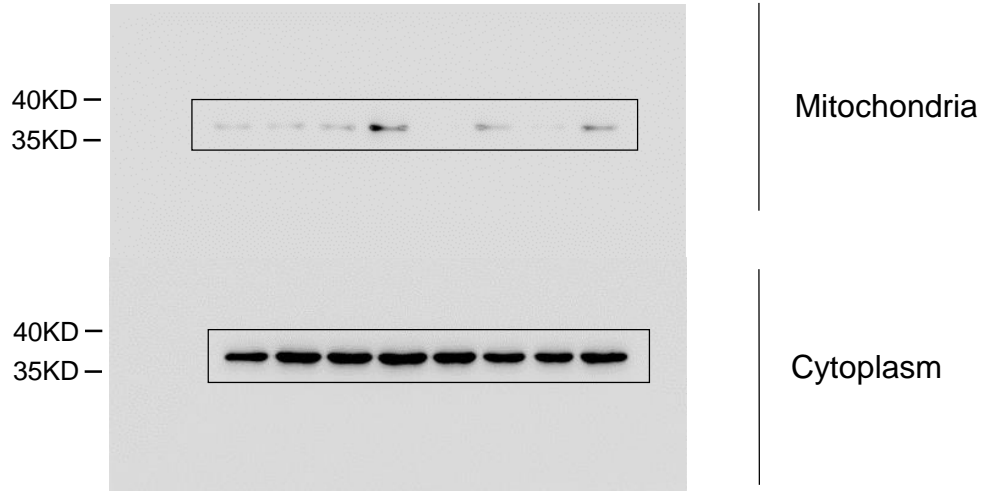

COX IV

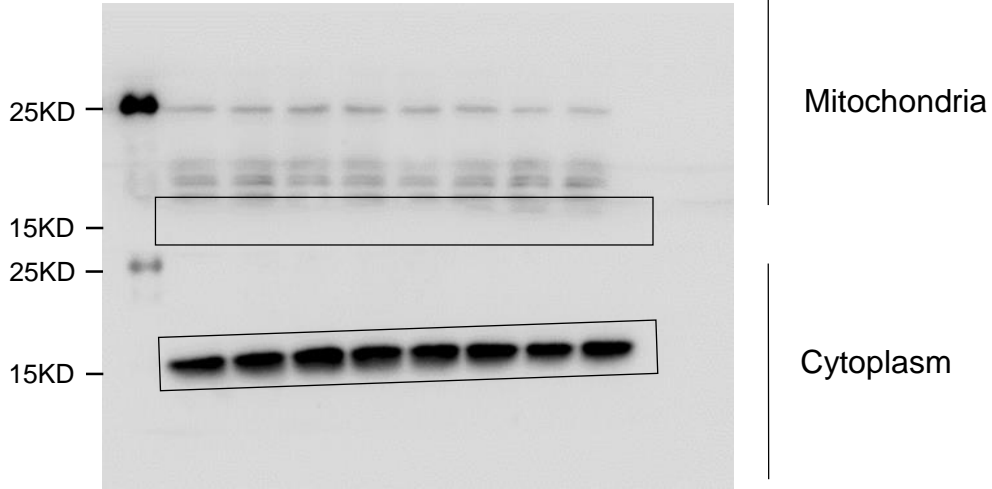

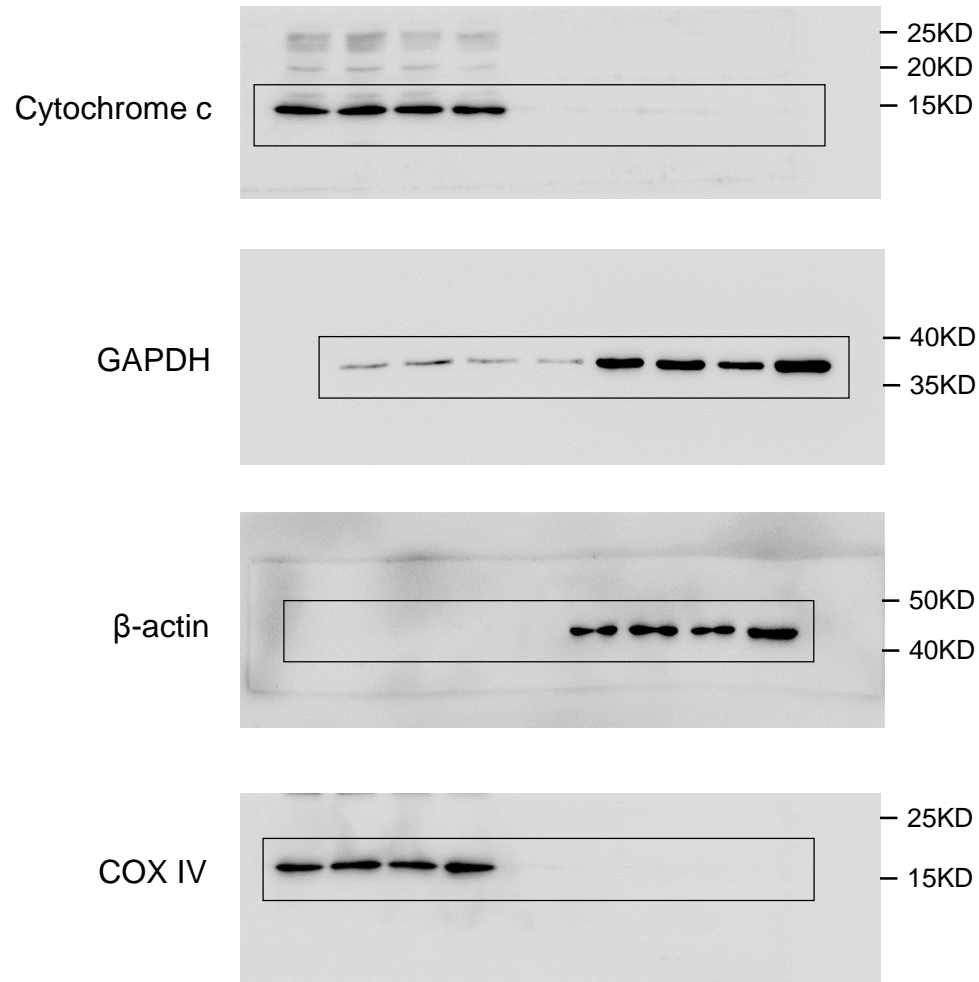

Supplement: Supplementary file 18 — Unprocessed western blots. [file 42255_2021_491_MOESM18_ESM.pdf]
